# Gut microbiota interspecies interactions shape the response of *Clostridioides difficile* to clinically relevant antibiotics

**DOI:** 10.1371/journal.pbio.3002100

**Published:** 2023-05-11

**Authors:** Susan Hromada, Ophelia S. Venturelli

**Affiliations:** 1 Department of Biochemistry, University of Wisconsin-Madison, Madison, Wisconsin, United States of America; 2 Microbiology Doctoral Training Program, University of Wisconsin-Madison, Madison, Wisconsin, United States of America; 3 Department of Bacteriology, University of Wisconsin-Madison, Madison, Wisconsin, United States of America; 4 Department of Chemical and Biological Engineering, University of Wisconsin-Madison, Madison, Wisconsin, United States of America; Georgia Institute of Technology, UNITED STATES

## Abstract

In the human gut, the growth of the pathogen *Clostridioides difficile* is impacted by a complex web of interspecies interactions with members of human gut microbiota. We investigate the contribution of interspecies interactions on the antibiotic response of *C*. *difficile* to clinically relevant antibiotics using bottom-up assembly of human gut communities. We identify 2 classes of microbial interactions that alter *C*. *difficile*’s antibiotic susceptibility: interactions resulting in increased ability of *C*. *difficile* to grow at high antibiotic concentrations (rare) and interactions resulting in *C*. *difficile* growth enhancement at low antibiotic concentrations (common). Based on genome-wide transcriptional profiling data, we demonstrate that metal sequestration due to hydrogen sulfide production by the prevalent gut species *Desulfovibrio piger* increases the minimum inhibitory concentration (MIC) of metronidazole for *C*. *difficile*. Competition with species that display higher sensitivity to the antibiotic than *C*. *difficile* leads to enhanced growth of *C*. *difficile* at low antibiotic concentrations due to competitive release. A dynamic computational model identifies the ecological principles driving this effect. Our results provide a deeper understanding of ecological and molecular principles shaping *C*. *difficile*’s response to antibiotics, which could inform therapeutic interventions.

## Abstract 2

This study examines how interactions between constituent members of the human gut microbiome influence the pathogen C. difficile’s response to clinically relevant antibiotics, providing key insights into ecological principles and molecular mechanisms that influence antibiotic susceptibility in this health-relevant system.

## Introduction

The bacterial pathogen *Clostridioides difficile* can infect the human gastrointestinal tract, an environment teeming with a dense microbiota. Gut microbiota can inhibit *C*. *difficile*’s growth and ability to persist over time in the human gut, a phenomenon known as colonization resistance [1]. The key role of colonization resistance is illustrated by the increased risk of *C*. *difficile* infection after treatment with antibiotics that decimate the microbiota [2] and by the efficacy of fecal microbiota transplants from healthy human donors in eliminating recurrent *C*. *difficile* infections [3]. Previous studies have provided a deeper understanding of interactions between gut microbiota and *C*. *difficile*. For example, interspecies interactions between individual gut microbes and *C*. *difficile* have been studied in vitro and analyses of human microbiome data have identified gut microbes whose presence or absence is associated with altered outcomes of *C*. *difficile* infection [4–6]. Multiple mechanisms of interaction have been determined, such as inhibition of *C*. *difficile* germination by *Clostridium scindens* via production of secondary bile acids [6] and promotion of *C*. *difficile* growth by *Bacteroides thetaiotaomicron* via succinate cross-feeding [7]. While much is known about how the microbiota impacts *C*. *difficile* growth, how the microbiota impacts *C*. *difficile* antibiotic susceptibility is largely unknown.

Similar to other pathogens, *C*. *difficile* antibiotic susceptibility has been studied using in vitro experiments of monoculture growth. However, monoculture experiments do not consider how interactions with resident community members can modify the antibiotic susceptibility of a pathogen. For example, monospecies antibiotic susceptibility of the pathogen *Pseudomonas aeruginosa* did not always correlate with the efficacy of treatment for polymicrobial infections [8]. If microbial interactions substantially decrease the pathogen’s antibiotic susceptibility, treatments based on monoculture susceptibility to antibiotics may not be effective in eradicating the pathogen. Alternatively, if communities increase the pathogen’s antibiotic susceptibility, the standard antibiotic dosage may exceed the dose needed to eradicate the pathogen, yielding unnecessary and avoidable disruption to the native microbiota. Understanding how constituent members of microbiota alter a pathogen’s susceptibility to an antibiotic could be used to guide the design of treatments to eradicate the pathogen.

Previous studies have shown that interspecies interactions can alter a given microbe’s response to antibiotics by increasing or decreasing susceptibility compared to monoculture [9]. One example is exposure protection, where susceptible microbes are protected from an antibiotic by species that degrade the antibiotic [10]. In addition, a previous study showed that an increase in antibiotic susceptibility occurred when the growth of a resistant microbe depended on cross-feeding with another organism that was susceptible to the antibiotic [11].

While previous studies have identified specific types of interspecies interactions that impact antibiotic susceptibility, the prevalence of susceptibility-altering microbial interactions across different microbial communities is not well understood. In a human urinary tract infection community, around a third of total interactions between species were estimated to yield a change in the susceptibility to 2 different antibiotics based on spent media experiments [12]. Studies of a fruit fly microbiome, a multispecies wound infection biofilm, and a multispecies brewery biofilm each identified a change in antibiotic susceptibility of a given species in the community compared to monospecies [13–15]. By contrast, no significant changes in antibiotic susceptibility were observed for 15 characterized species in a community of human gut microbes or an *Escherichia coli* pathogen introduced into a porcine microbiome [16,17]. Based on this observed variation in the contribution of interspecies interactions to antibiotic susceptibility across different systems, it is not known whether microbial interactions can impact *C*. *difficile*’s antibiotic susceptibility.

We used a diverse human gut community [4] to study the impact of microbial interactions on *C*. *difficile’s* antibiotic susceptibility. We focused on 2 antibiotics, vancomycin and metronidazole, that are used to treat *C*. *difficile* infections. Vancomycin is a glycopeptide that inhibits cell wall synthesis whose activity is specific to gram-positive bacteria [18]. Metronidazole is a DNA-damaging agent that is effective against both gram-positive and gram-negative anaerobic bacteria. Metronidazole is a prodrug which is inactive until its nitro group is reduced to nitroso radicals in the cytoplasm of anaerobic bacteria [19]. The proposed mechanism of metronidazole reduction is due to the cofactors ferredoxin and/or flavodoxin in reactions catalyzed by multiple enzymes including reductases, hydrogenases, and pyruvate ferredoxin/flavodoxin oxidoreductase (PFOR) [20–22]. Metronidazole, previously a recommended first-line treatment, is now only recommended in rare cases due to an observed decrease in its clinical effectiveness [23,24].

Using a bottom-up approach, we perform a detailed and quantitative characterization of how human gut microbes impact *C*. *difficile’*s response to these antibiotics. We show that gut microbes infrequently alter *C*. *difficile*’s minimum inhibitory concentration (MIC), but gut microbes frequently impact *C*. *difficile*’s response to subinhibitory concentrations of antibiotics. In communities with antibiotic-sensitive species that also compete with *C*. *difficile*, we observe that *C*. *difficile’s* growth is enhanced in the presence of low concentrations of antibiotics due to competitive release. A dynamic ecological model representing the antibiotic recapitulates these trends. In addition, we demonstrate that *Desulfovibrio piger* substantially increases *C*. *difficile*’s MIC for metronidazole. We investigate the mechanism determining the increased MIC of *C*. *difficile* using transcriptional profiling and media perturbations. Our data suggest *D*. *piger*’s impact on the environment induces a metal starvation transcriptional response in *C*. *difficile*, leading to down-regulation of enzymes required to reduce metronidazole to its active form. In sum, biotic interactions shape *C*. *difficile* antibiotic susceptibility at both subinhibitory and minimal inhibitory concentration regimes via distinct mechanisms. These results highlight the need to consider biotic interactions in the design of future therapeutic treatments to eradicate pathogens.

## Results

### The minimum inhibitory concentration of *C*. *difficile* to metronidazole or vancomycin is modified by a subset of gut microbes in pairwise communities

**Fig 1:**
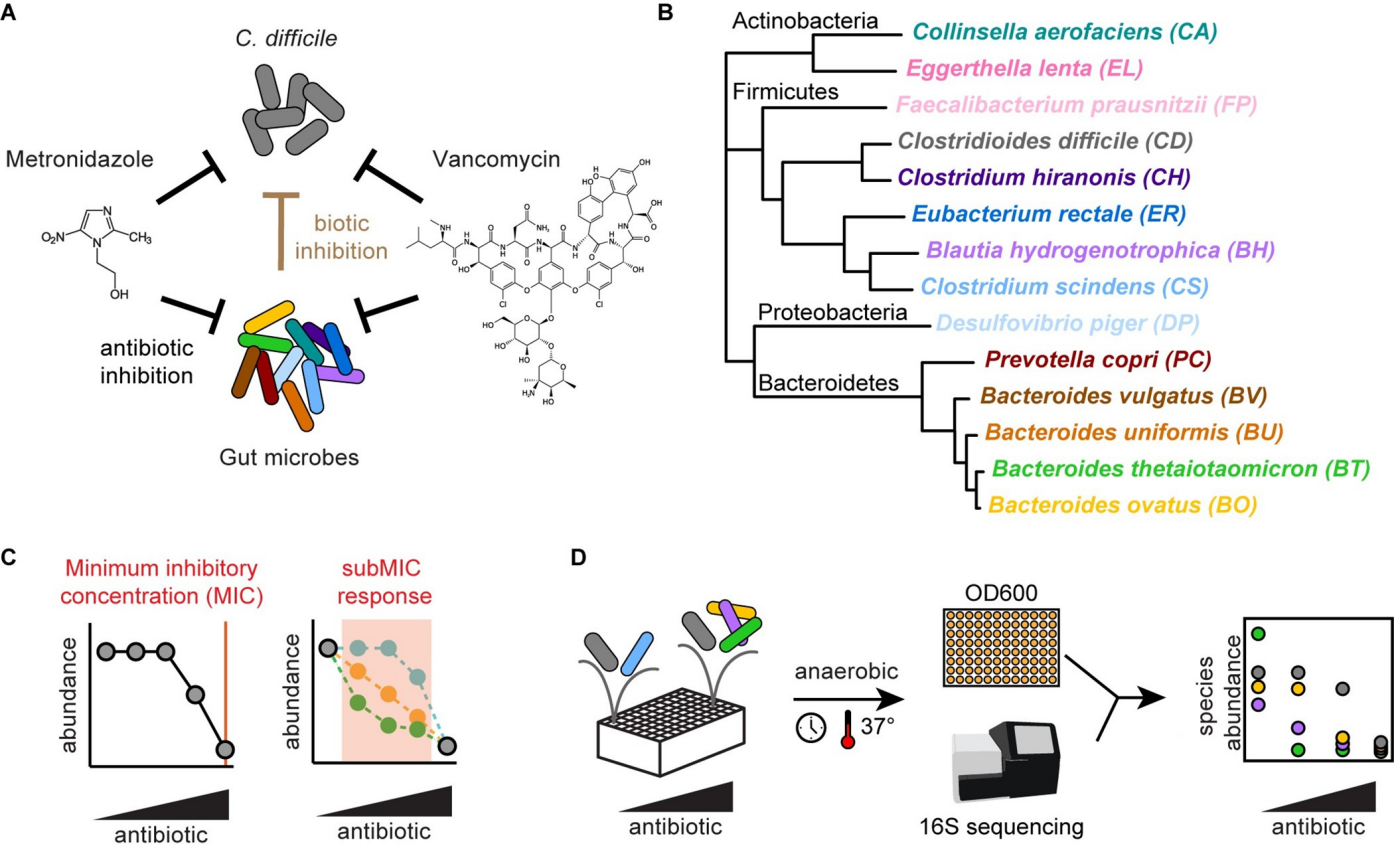
Investigating the effects of interspecies interactions on the clinically relevant antibiotic response of *C*. *difficile* in human gut communities. ** (A)** Schematic of factors affecting *C*. *difficile* growth in synthetic gut communities in the presence of antibiotics metronidazole or vancomycin. The DNA-damaging antibiotic metronidazole and the cell wall inhibitor vancomycin can inhibit *C*. *difficile* and gut microbes (black arrows). Biotic inhibition by gut microbes (brown arrow) inhibits *C*. *difficile*. **(B)** Phylogenetic tree of 14-member human gut community, spanning 4 major phyla of gut microbiota and pathogen *C*. *difficile*. Black text indicates phylum name. Colored text indicates species name. Phylogeny based on a concatenated alignment of 37 marker genes [25]. **(C)** Schematic of 2 aspects of the response of a microbial population to antibiotics: the MIC and the subMIC response. **(D)** Schematic of methods to determine the antibiotic susceptibility of multispecies communities. Communities are incubated anaerobically in microtiter plates. Absolute abundance is determined by multiplying optical density (OD600) by relative abundance based on 16S rRNA gene sequencing. MIC, minimum inhibitory concentration; subMIC, sub-minimum inhibitory concentration.

*C*. *difficile* infections occur in the context of complex resident gut communities. However, antibiotic treatments to eliminate *C*. *difficile* infection are designed based on the susceptibility of *C*. *difficile* in monoculture, which neglect the role of interspecies interactions in shaping antibiotic susceptibility. Understanding how the gut microbiota alters *C*. *difficile* antibiotic susceptibility could inform the treatment of *C*. *difficile* by antibiotics. To investigate this question, we evaluated *C*. *difficile’s* response to antibiotics in the presence of synthetic communities of gut microbes (**Fig 1A**). The 13-member human gut community was designed to span the phylogenetic diversity of the human gut microbiome. The interactions between these gut microbes and *C*. *difficile* have been deciphered in the absence of antibiotics [4] (**Fig 1B**). We selected the antibiotics metronidazole and vancomycin for their clinical relevance in the treatment of *C*. *difficile* infections and for the differences in their activity spectra. Metronidazole has broad spectrum activity against the 13 gut microbes, whereas the activity of vancomycin is specific for gram-positive species. To determine how *C*. *difficile* would respond to both low and high antibiotic concentrations in the human gut, we characterized *C*. *difficile*’s MIC and its growth in the presence of subinhibitory concentrations across a range of ecological contexts (**Fig 1C**).

To determine the antibiotic susceptibility of *C*. *difficile* in communities, we used a broth dilution method based on the clinical method detailed by the Clinical and Laboratory Standards Institute [26] (Methods). We inoculated communities in liquid culture containing a 2-fold dilution series of antibiotics. After incubation, we determined the absolute abundance of each species in the communities at a fixed time point (48 h) by multiplying the community optical density at 600 nm (OD600) by its relative abundance determined via 16S rRNA gene sequencing (**Fig 1D**). The MIC for each species in the community was determined as the lowest antibiotic concentration where growth at 48 h was below a threshold (Methods).

**Fig 2:**
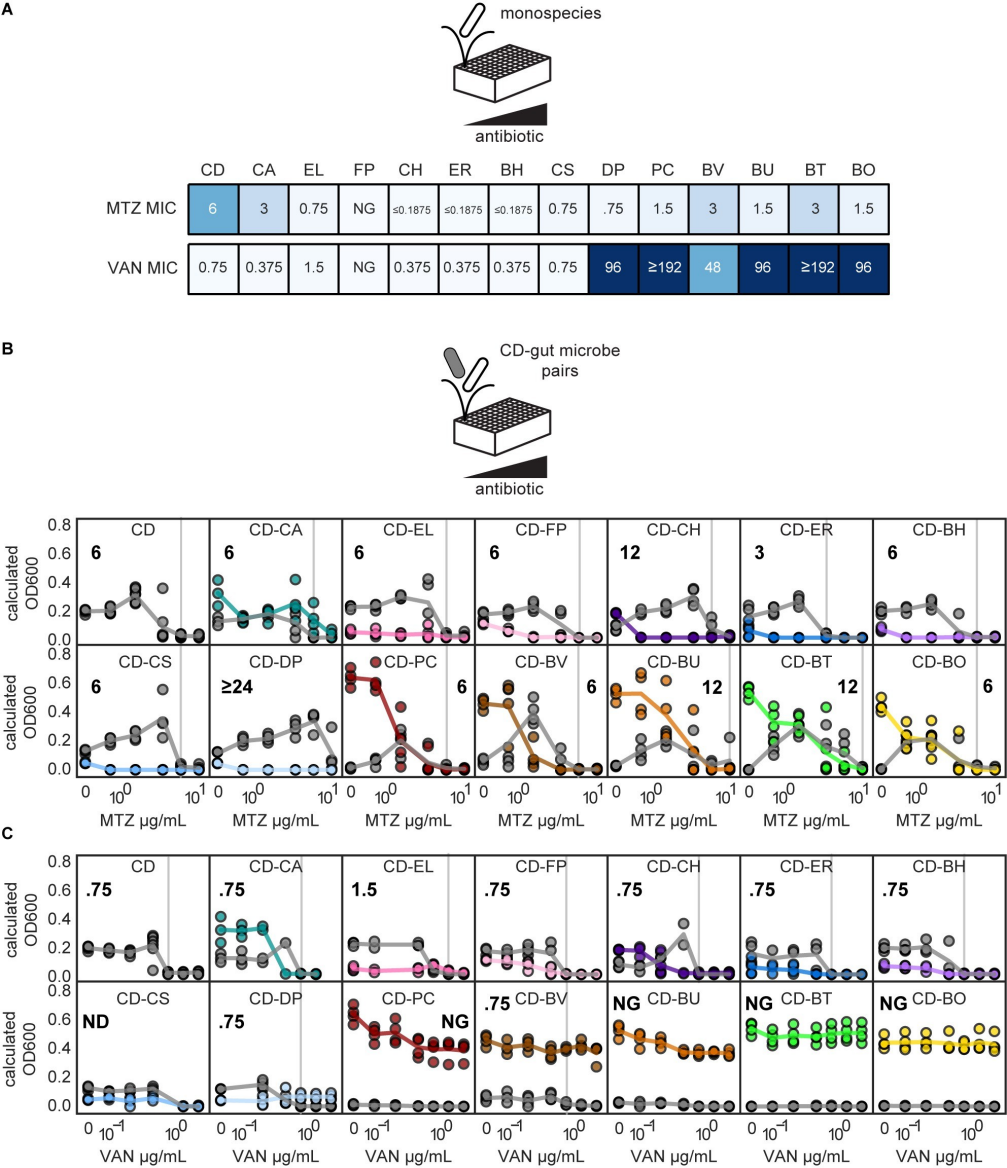
*C*. *difficile* response to metronidazole or vancomycin is modified in specific pairwise communities. **(A)** Heatmaps of MIC of monocultures in the presence of metronidazole (MTZ) or vancomycin (VAN). Species indicated by 2-letter species code (Fig 1B). **(B, C)** Line plots of species absolute abundance at 48 h as a function of antibiotic concentration for *C*. *difficile* in monoculture or *C*. *difficile* in pairwise communities. Each x-axis is semi-log scale. The values on the y-axis are calculated OD600 (OD600 multiplied by relative abundance from 16S rRNA gene sequencing). Data points indicate individual biological replicates. Lines indicate the average of *n* = 1 to *n* = 8 biological replicates. Vertical gray line and bold number indicate MIC of *C*. *difficile* in each condition. Color indicates species (Fig 1B). The data underlying all panels in this figure can be found in DOI: 10.5281/zenodo.7626486. MIC, minimum inhibitory concentration.

Using this method, we determined the MIC of each species in monoculture (**Fig 2A**) and in each pairwise community containing *C*. *difficile* (**Fig 2B and 2C**). Consistent with the mechanism of the antibiotics, all species were susceptible to metronidazole in the range of antibiotics tested, whereas gram-negative species (Bacteroidetes and Proteobacteria phyla, **Fig 1D**) were not susceptible to vancomycin (**Figs 2A** and **S1A and S1B**).

In most pairwise communities, the metronidazole and vancomycin MICs of *C*. *difficile* were unchanged compared to monoculture. Across metronidazole and vancomycin conditions, the MIC of *C*. *difficile* was unchanged in 15 pairwise communities and displayed a moderate difference (2-fold) in 5 pairwise communities compared to monospecies (**Fig 2B and 2C**). However, *C*. *difficile* had a large change in MIC (≥4-fold) in coculture with *D*. *piger* (metronidazole MIC increased from 6 μg/mL to greater than or equal to 24 μg/mL) (**Fig 2B**). In the remaining 4 pairwise communities, *C*. *difficile’s* growth was strongly inhibited in all concentrations and no MIC could be calculated (**Fig 2C**, indicated as “NG”). In sum, the MIC of *C*. *difficile* was infrequently impacted by pairwise interspecies interactions in the human gut community.

For select pairwise communities, we quantified *C*. *difficile* abundance using both OD600 multiplied by 16S rRNA gene sequencing relative abundance and count of colony-forming units (CFUs) on *C*. *difficile* selective agar plates to validate the OD600-based absolute abundance method (**S2A Fig**). Our results showed that CFU counting and the OD600-based absolute abundance method yielded the same MIC value in 11 out of 12 conditions (**S2B and S2C Fig**). In 1 condition, *C*. *difficile*’s growth was strongly inhibited below the MIC threshold for all concentrations. Therefore, an MIC could not be determined using the OD600-based method, although an MIC could be revealed using the CFU method. Overall, these trends demonstrate that the OD600-based absolute abundance method is a reliable way to measure *C*. *difficile* MIC, although the 2 methods can vary in sensitivity.

### Enhancement of *C*. *difficile* abundance in pairwise communities in the presence of low antibiotic concentrations

Pathogens may encounter antibiotic concentrations lower than the MIC during antibiotic treatments. If the pathogen has gained resistance to the antibiotic, the entire dose regimen could be subinhibitory. Alternatively, even if the target dose is greater than the MIC, sub-minimum inhibitory concentrations (subMICs) can occur at the beginning and end of dosing regimens and between daily dosages [27]. Therefore, we investigated the role of microbial interactions on *C*. *difficile’*s response to subinhibitory antibiotic concentrations (concentrations lower than *C*. *difficile*’s MIC or subMICs).

**Fig 3:**
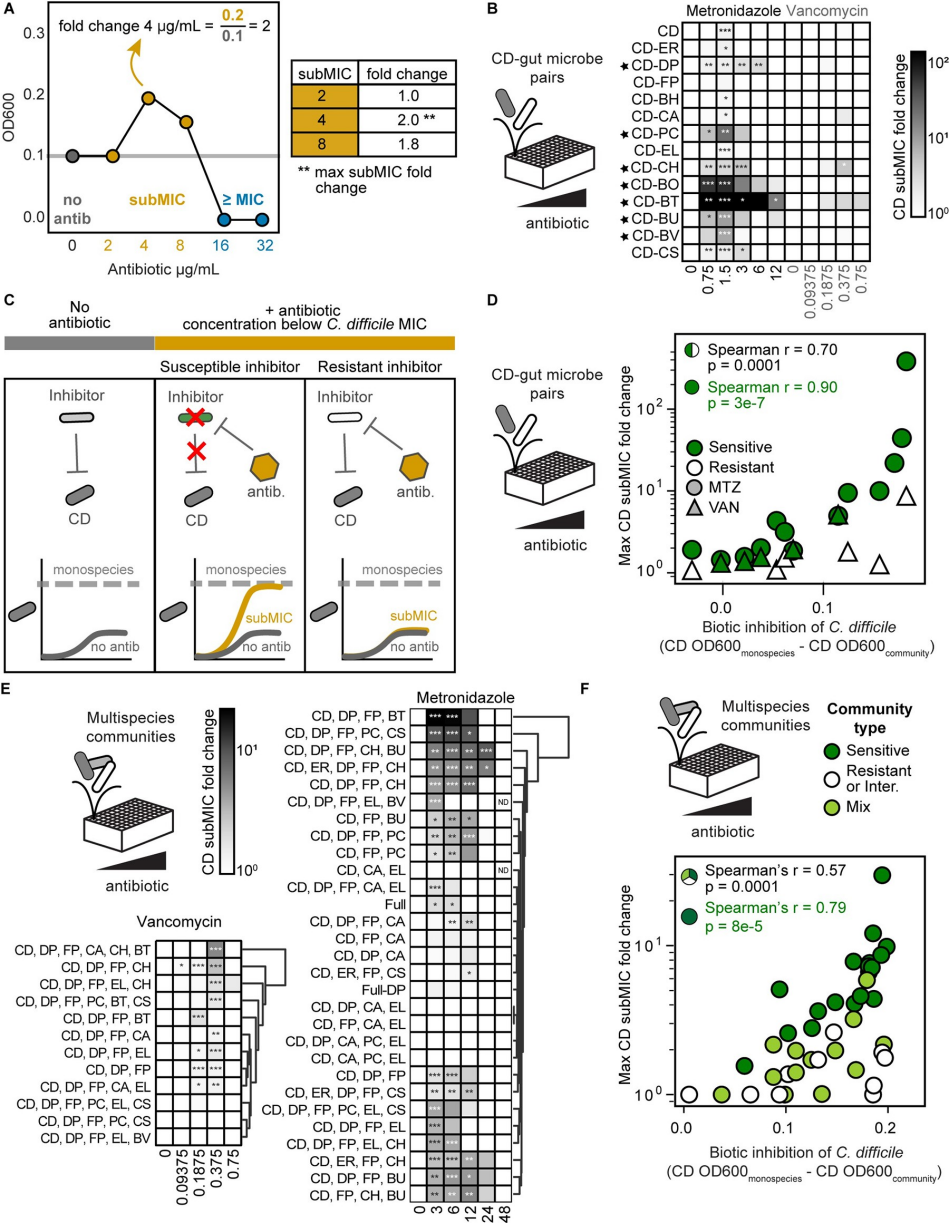
*C*. *difficile* growth is enhanced in communities with antibiotic-sensitive biotic inhibitors in the presence of antibiotic concentrations lower than the MIC. **(A)** Schematic of subMIC fold change metric using example data. The x-axis is semi-log scale. Table indicates subMIC fold changes for the 3 subMICs (2, 4, and 8 μg/mL) in this example. Maximum subMIC fold change (indicated by **) is defined as the largest fold change across all subMICs. **(B)** Heatmap of subMIC fold changes for *C*. *difficile* in pairwise communities at each concentration at a 48 h. Shading indicates subMIC fold change. SubMIC fold change is calculated as the average *C*. *difficile* absolute abundance at the subMIC divided by the average *C*. *difficile* absolute abundance in the absence of antibiotic, where the average is of *n* = 1 to *n* = 8 biological replicates. Asterisks indicate significant difference (*P < 0.05, **P < 0.01, ***P < 0.001) between *C*. *difficile* OD600 at subMIC and *C*. *difficile* OD600 in the absence of antibiotic according to an unpaired *t* test. Black stars indicate pairs with a maximum subMIC fold change significantly greater than the maximum subMIC fold change of *C*. *difficile* monospecies (*p* < 0.05 based on an unpaired *t* test). **(C)** Schematic of *C*. *difficile* growth in the presence of antibiotic sensitive and resistant inhibitors. **(D)** Scatterplot of maximum *C*. *difficile* subMIC fold change in pairwise communities as a function of the degree of biotic inhibition of *C*. *difficile* in pairwise communities for metronidazole (MTZ, circles) and vancomycin (VAN, triangles). Biotic inhibition of *C*. *difficile* (x-axis) is defined as the average *C*. *difficile* OD600 in monospecies minus the average *C*. *difficile* OD600 in the pairwise community in no antibiotic conditions. The maximum *C*. *difficile* subMIC fold change (y-axis) is the maximum of the *C*. *difficile* subMIC fold change across all subMICs at 48 h. The subMIC fold change is calculated as in panel B, with *n* = 1 to *n* = 8 biological replicates. Species are classified as sensitive if the species monospecies MIC was less than the monospecies MIC of *C*. *difficile* and resistant if the species monospecies MIC was greater than or equal to the monospecies MIC of *C*. *difficile*. Each point represents 1 pairwise community in metronidazole or vancomycin. The Spearman correlation is annotated for all data points (black) and for pairwise communities with sensitive gut microbes only (green). **(E)** Heatmap of subMIC fold changes for *C*. *difficile* in multispecies communities at each concentration of metronidazole and vancomycin at 48 h. Shading indicates subMIC fold change. SubMIC fold change is calculated as in panel B, with *n* = 1 to *n* = 4 biological replicates. Asterisks indicate significant difference (*P < 0.05, **P < 0.01, ***P < 0.001) between *C*. *difficile* OD600 at subMIC and *C*. *difficile* OD600 in the absence of antibiotic according to an unpaired *t* test. Communities clustered by subMIC fold change using Euclidean distance hierarchical clustering. “Full” indicates 14-member community, “Full-DP” indicates 13-member community containing all species except DP. **(F)** Scatterplot of maximum *C*. *difficile* subMIC fold change in multispecies communities as a function of the degree of biotic inhibition of *C*. *difficile* in multispecies communities for metronidazole (circles) and vancomycin (triangles). Biotic inhibition of *C*. *difficile* (x-axis) is calculated as in panel D. The maximum *C*. *difficile* subMIC fold change (y-axis) is calculated as in panel B, with *n* = 1 to *n* = 4 biological replicates. Community type is sensitive (“Sens.”) if all species excluding *C*. *difficile* are sensitive inhibitors. Community type is resistant or intermediate (“Resis. or Intermed.”) if all species excluding *C*. *difficile* are either intermediate-inhibitors or resistant-inhibitors. Community type is considered “mix” if the given community contains both sensitive inhibitors and resistant- or intermediate-inhibitors. Species are categorized as biotic inhibitors if the absolute abundance of *C*. *difficile* in the presence of this species was significantly lower than the absolute abundance of *C*. *difficile* in monospecies, in the absence of antibiotics, as determined by an unpaired *t* test. Species are classified as sensitive and resistant as in panel D. *C*. *aerofaciens* was classified as intermediate (see text). Each point represents 1 community in the presence of metronidazole or vancomycin. The Spearman correlation is annotated for all data points (black) and for sensitive communities only (green). The data underlying panels BDEF in this figure can be found in DOI: 10.5281/zenodo.7626486. MIC, minimum inhibitory concentration; subMIC, sub-minimum inhibitory concentration.

In many pairwise communities, *C*. *difficile’*s abundance was similar or reduced in the subMIC range than in the absence of antibiotic (**Fig 2B and 2C**). However, in a subset of pairwise communities, *C*. *difficile*’s abundance in the subMIC range was enhanced compared to the absence of antibiotic (**Fig 2B and 2C**). For example, *C*. *difficile* monospecies abundance was similar in the presence of 0.75 μg/mL metronidazole and the absence of antibiotic. However, in the presence of *B*. *thetaiotaomicron*, the abundance of *C*. *difficile* was significantly higher in the presence of 0.75 μg/mL metronidazole than in the absence of antibiotic (**Fig 2B**). To provide further insights, we computed the subMIC fold change at each subMIC, defined as the species absolute abundance at the given subMIC divided by the species absolute abundance in the absence of antibiotic (**Fig 3A**). Using this metric, *C*. *difficile*’s growth was enhanced for at least 1 subMIC in 1 pairwise community in the presence of vancomycin and 11 pairwise communities in the presence of metronidazole (**Figs 3B** and **S3A and S3B**). In 7 pairwise communities, the growth enhancement of *C*. *difficile* in the presence of metronidazole was significantly greater than the enhancement observed for the *C*. *difficile* monoculture in the presence of metronidazole (**Fig 3B**). In sum, our results illustrate that approximately half of the species altered *C*. *difficile*’s response to low concentrations of metronidazole.

In monoculture, *C*. *difficile*’s growth was moderately enhanced in the presence of a single subMIC (1.5 μg/mL metronidazole, subMIC fold change of 1.6, **S3A Fig**). Time-series OD600 measurements of *C*. *difficile* monoculture revealed that this moderate growth enhancement was due to a difference in growth phase in the presence and absence of the antibiotic at the measured time point (48 h) (**S3C and S3D Fig**). By contrast, *C*. *difficile*’s growth was enhanced in 2 pairwise communities in the presence of subMICs compared to no treatment over multiple time points that was not due to differences in growth phase (**S3E and S3F Fig**). Thus, while differences in monoculture growth phases yielded a modest enhancement of *C*. *difficile* in response to a single subMIC, a different mechanism determined the observed larger and sustained growth enhancements in pairwise communities.

We hypothesized the action of the antibiotic in certain pairwise communities relieved the biotic inhibition of *C*. *difficile*, which would in turn enhanced *C*. *difficile* growth, representing competitive release [28,29]. This competitive release would occur in pairwise communities with competitors (i.e., biotic inhibitors) with a higher susceptibility to the antibiotic than *C*. *difficile*. By contrast, if a biotic inhibitor displayed a higher resistance to the antibiotic than *C*. *difficile*, the degree of biotic inhibition would remain unchanged at subMICs, and thus, *C*. *difficile*’s growth would not change substantially compared to its growth in absence of the antibiotic (**Fig 3C**). Supporting this hypothesis, the maximum subMIC fold change of *C*. *difficile* displayed a strong positive correlation with the degree of biotic inhibition in the presence of the coculture partner (**Fig 3D**). In addition, the correlation was enhanced in pairwise communities containing gut microbes that were sensitive to the antibiotic (**Fig 3D**). In the presence of both individual antibiotics, the maximum subMIC fold change of *C*. *difficile* was significantly higher when paired with biotic inhibitors with higher sensitivity to the antibiotic than *C*. *difficile* (**S3G Fig**). In sum, antibiotic induced inhibition of susceptible biotic inhibitors enhanced *C*. *difficile*’s growth in response to subMICs. This suggests that *C*. *difficile*’s growth in the gut could be enhanced in the subMIC regime if patients harbor antibiotic-sensitive biotic inhibitors.

We investigated whether the *C*. *difficile* subMIC growth enhancements measured using the OD600-based absolute abundance method were due to increases in *C*. *difficile* abundance as opposed to other factors such as changes in cell morphology. To this end, we determined the subMIC fold changes of *C*. *difficile* in select pairwise communities that were characterized using CFU counting (**S2B and S2C Fig**). Our results showed that the CFU method and OD600-based absolute abundance method identified a significant subMIC fold change in the *C*. *difficile-B*. *thetaiotaomicron* and *C*. *difficile-D*. *piger* pairs in presence of metronidazole (**S2D Fig**). The methods also agreed in detecting no significant subMIC fold changes in 10 other pairwise communities in metronidazole or vancomycin (**S2D Fig**). However, the CFU method did not detect significant subMIC fold changes in 3 pairwise communities for which the OD600-based method detected significant fold changes (*C*. *difficile-C*. *hiranonis* vancomycin and metronidazole conditions and *C*. *difficile-C*. *scindens* metronidazole condition, **S2D Fig**). These discrepancies may be due to differences in sensitivities of the 2 methods, as the spot dilution CFU method (Methods) cannot distinguish small fold changes (average variation of technical replicates is 2-fold).

To determine if subMIC fold changes could be resolved using the lower sensitive CFU method, we increased the resolution of subinhibitory concentrations tested. The finer resolution may capture subMICs with larger fold changes that are large enough to be detectable by the CFU method. Additionally, the finer resolution may result in multiple subMICs with significant fold changes, increasing confidence in the results. We used the CFU method with a finer resolution of antibiotic concentrations to measure growth enhancement in the *C*. *difficile-B*. *vulgatus* pairwise community in the presence of metronidazole, which exhibited a significant *C*. *difficile* growth enhancement using the OD600-based method (**Fig 3B**). Our results demonstrated a significant *C*. *difficile* subMIC fold change at multiple concentrations in the pairwise community, while no significant fold changes were observed in the *C*. *difficile* monoculture (**S4 Fig**). This demonstrates that the observed *C*. *difficile* growth enhancement using the OD600-based method (**Fig 3B**) was consistent with CFU counting. Overall, our results demonstrate that *C*. *difficile* subMIC growth enhancements can also be detected by the CFU-based absolute abundance method.

### Enhancement of *C*. *difficile* abundance in multispecies communities in the presence of low antibiotic concentrations

We investigated if the OD600-based absolute abundance trends observed in pairwise communities persisted in multispecies communities that are more representative of human gut microbiota. The communities consisted of 2- and 3-member core communities (CC) predicted to display minimal biotic inhibition of *C*. *difficile* guided by a previously developed dynamic computational model of our system [4] (CC-2: *D*. *piger* and *Faecalibacterium prausnitzii*. CC-3: *D*. *piger*, *F*. *prausnitzii*, and *Eggerthella lenta*). In addition to this core community, we introduced at least 1 antibiotic-sensitive inhibitor, antibiotic-resistant inhibitor, or a combination of species in these 2 groups, creating 10 communities for metronidazole and 12 communities for vancomycin (3- to 6-members). To further explore the behavior of communities in response to antibiotic perturbations, we characterized the response of 3-, 4-, 5-, 13-, or 14-member communities (19 total) with no consistent core members to metronidazole.

In 6 of 29 communities characterized in the presence of metronidazole, *C*. *difficile*’s MIC was 4-fold greater than in monoculture, increasing from 6 μg/mL to 24 μg/mL. The remaining communities displayed moderate (2-fold, 14 communities) or no change (9 communities) (**S5A Fig, S1 Table**). In 4 of 12 communities examined in the presence of vancomycin, *C*. *difficile’s* growth was strongly inhibited at all concentrations and no MIC could be calculated (**S5B Fig, S2 Table,** indicated as “NG”). The remaining 8 communities displayed either a moderate increase in MIC (2-fold, 2 communities) or no change (6 communities) (**S5B Fig**, **S2 Table**). Consistent with the pairwise community data, *C*. *difficile’s* metronidazole MIC was altered in certain communities, but *C*. *difficile’s* vancomycin MIC was not substantially altered in any of the characterized communities.

We tested if the multispecies communities containing antibiotic-sensitive biotic inhibitors displayed an enhancement in *C*. *difficile*’s growth at subMICs. In response to metronidazole or vancomycin, *C*. *difficile’s* growth was enhanced in response to at least 1 subMIC in most communities (21 of 29 communities for metronidazole, 9 of 12 communities for vancomycin, **Figs 3E and S6**). Consistent with the pairwise community data, the degree of biotic inhibition of *C*. *difficile* was positively correlated with the magnitude of the maximum subMIC fold change (**Fig 3F**). In addition, the correlation was stronger for communities composed of only antibiotic-sensitive biotic inhibitors (**Fig 3F**). Consistent with these trends, the number of sensitive inhibitors in the community and the absolute abundance of sensitive inhibitors displayed a positive correlation with the maximum subMIC fold change (**S7A Fig**). Communities containing antibiotic-sensitive biotic inhibitors had a significantly higher average subMIC fold change than communities containing only resistant inhibitors or a mix of both inhibitor types (**S7B Fig**).

Communities containing a mix of antibiotic sensitive and resistant inhibitors, mirroring the makeup of the gut microbiota, displayed low subMIC fold changes similar in magnitude to resistant biotic inhibitor communities (**S7B Fig**). In communities with a non-zero abundance of resistant biotic inhibitors, the correlation between the growth enhancement of *C*. *difficile* and the number of sensitive inhibitors or abundance of sensitive inhibitors vanished (**S7A Fig**). These data suggest that resistant biotic inhibitors can suppress the *C*. *difficile* growth enhancement caused by sensitive biotic inhibitors. We analyzed if the growth enhancement of *C*. *difficile* in multispecies communities could be predicted based on the sum of the growth enhancements of *C*. *difficile* in pairwise communities. While the relationship displayed a moderate correlation, it was not additive (**S7C and S7D Fig**). This demonstrates that community effects, such as interspecies interactions between constituent community members and growth enhancement suppression by resistant inhibitors, play key roles in determining the magnitude of growth enhancement of *C*. *difficile* at subMICs. Overall, these results demonstrate that *C*. *difficile* growth enhancement at subMICs can occur in multispecies communities, suggesting this response may occur in the human gut microbiome.

### A dynamic ecological model representing pairwise interactions and monospecies antibiotic susceptibilities can capture the response of *C*. *difficile* to subMICs

We hypothesized that a model capturing the dynamics of species growth, interspecies interactions, and monospecies antibiotic susceptibilities could recapitulate the observed trends in *C*. *difficile* growth enhancement at subMICs. We assumed that inferred interspecies interactions in the absence of antibiotics contributed to the community response to antibiotics. We tested whether a model that neglects complex antibiotic-dependent interspecies interactions could predict the qualitative trends of *C*. *difficile* growth at subMICs.

**Fig 4:**
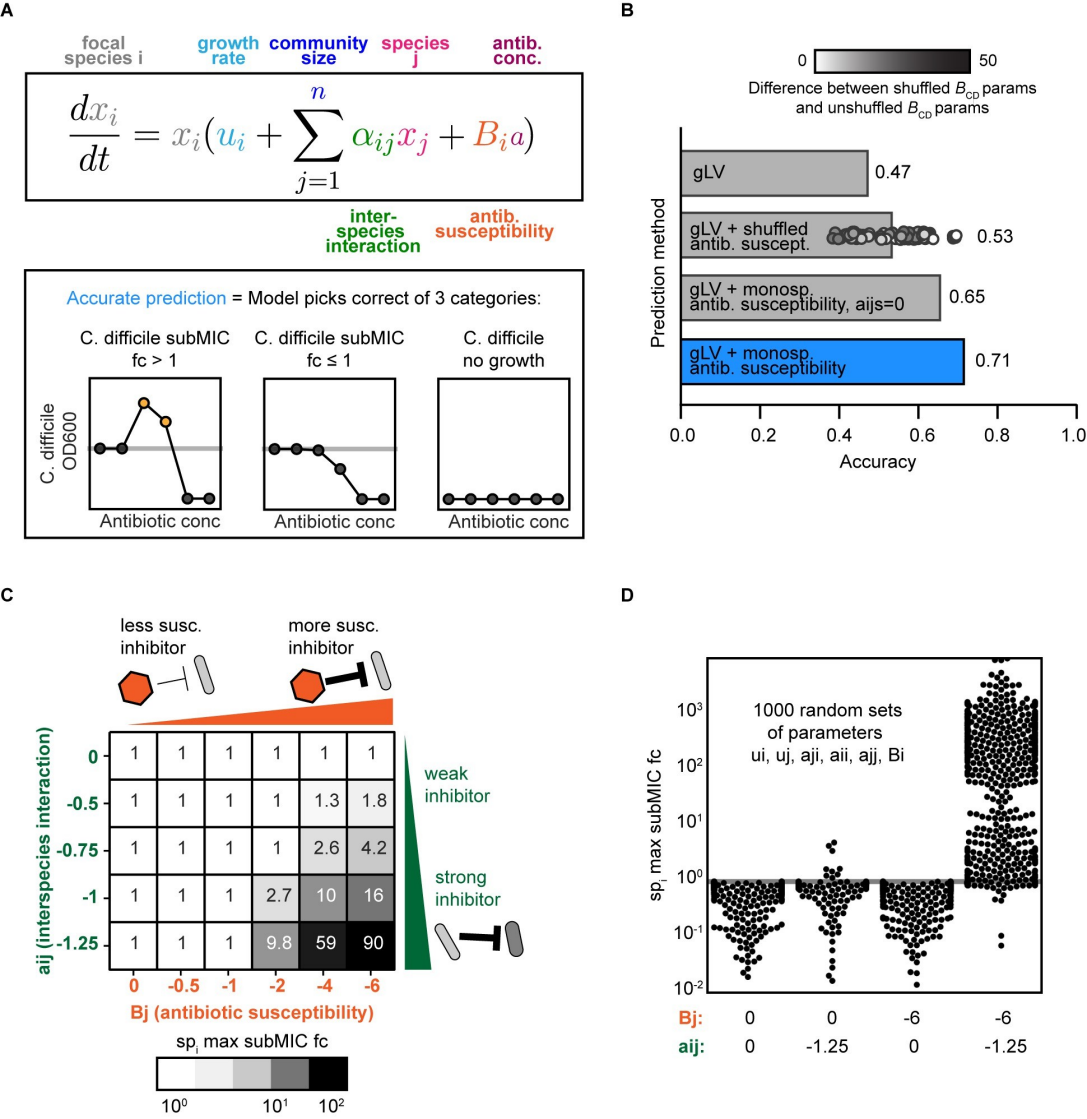
A modified generalized Lotka–Volterra model of community dynamics in response to antibiotics captures trends in antibiotic concentrations lower than the MIC in the presence of antibiotic sensitive biotic inhibitors. **(A)** Top: Schematic of the antibiotic expansion of the gLV model. Bottom: Schematic of accuracy metric used in panel B. (B) Qualitative accuracy of multiple models for pairwise and multispecies communities. Models include (1) standard gLV model lacking an antibiotic term; (2) gLV model with randomly shuffled antibiotic susceptibility parameters (average of 100 permutations), and 100 shuffled values shown as points, shaded according to the absolute value of the difference between the shuffled *C*. *difficile* antibiotic susceptibility parameter and the original *C*. *difficile* antibiotic susceptibility parameter; (3) gLV model with antibiotic susceptibility terms inferred from monospecies with all interaction parameters set to zero (a_ij_ = 0, where *i*! = *j*); (4) full gLV model with antibiotic susceptibility terms inferred from monospecies data. (C) Simulated maximum subMIC fold changes for a focal species in a pairwise community for 48 h for a representative parameter set. Growth rates of the 2 species are equal (u_i_ = u_j_ = 0.25), intraspecies interactions are equal (a_ii_ = a_jj_ = −0.8), and initial ODs are equal (x_i_(0) = x_j_(0) = 0.0022). The interspecies interaction coefficients a_ji_ are set to 0 and B_i_ is set to −2. (D) Simulated max subMIC fold change for a focal species in a pairwise community for 1,000 randomly sampled parameter sets. Parameters *B*_j_ and a_ij_ are set to constant values of 0 or −6 and 0 or −1.25, respectively. Other parameters were randomly sampled between upper and lower bounds. The lower and upper bounds on each parameter value are a_ji_ (−1.25, 1.25), growth rates (0, 1), intraspecies interactions (−1.25, 0), *B*_i_ (−6, 0). Gray horizontal line at y = 1 indicates no change in growth compared to the no antibiotic condition. The data and modeling scripts underlying panels BCD in this figure can be found in DOI: 10.5281/zenodo.7726490. MIC, minimum inhibitory concentration; subMIC, sub-minimum inhibitory concentration.

The gLV model is a system of coupled ordinary differential equations that captures individual species’ growth rate and pairwise interactions with each community member. We use an expansion of the generalized Lotka–Volterra model (gLV) that captures antibiotic perturbations [30] (**Fig 4A**). In this expanded model, the growth of each species is modified by an antibiotic term consisting of the concentration of antibiotic and the susceptibility of each species (B_i_) (**Methods**, **Fig 4A**).

The monospecies antibiotic susceptibility parameters were inferred from measurements of individual species growth in the presence of a range of antibiotic concentrations (2-fold dilutions) (Methods, **S1 Fig**). We used growth rate and interspecies interaction parameters that were previously inferred based on absolute abundance measurements of 159 monospecies and communities (2- to 14-member) in the absence of antibiotics [4].

We evaluated whether this model could qualitatively predict the trends in growth enhancement of *C*. *difficile* in the subMIC range (**S8 and S9 Figs**). *C*. *difficile*’s response to each antibiotic concentration was classified into 3 categories (*C*. *difficile* subMIC fold-change > 1, *C*. *difficile* subMIC fold-change ≤ 1, or no growth of *C*. *difficile*, **Fig 4A**). The expanded gLV model correctly predicts *C*. *difficile*’s qualitative response to 71% of antibiotic conditions (**Figs 4B** and **S10A**). We designed a set of null models to evaluate the contribution of different terms of the expanded gLV model to model performance. The full model displays higher accuracy than a null model that lacked antibiotic susceptibility terms or has randomly shuffled antibiotic susceptibility parameters (47% and 53% accuracy, **Fig 4C**). These data highlight that the fitted monospecies susceptibilities play a major role in the model’s predictive performance. The full model also outperforms a null model that lacks interspecies interaction terms (65%, **Fig 4C**), indicating that the inferred interspecies interactions contribute to the growth enhancement of *C*. *difficile* in response to subMICs. Taken together, these findings indicate that biotic inhibition and monospecies antibiotic susceptibility are major variables determining the growth of *C*. *difficile* in response to subMICs in microbial communities.

We explored model simulations to determine if the enhancement of *C*. *difficile* growth at subMICs required a sensitive biotic inhibitor in a wide variety of communities, beyond those that were experimentally characterized. To this end, we simulated 1,000 pairwise communities with a wide range of growth rates, interspecies interactions, and antibiotic susceptibilities. When paired with a sensitive biotic inhibitor, growth is enhanced at subMICs, consistent with the trends observed in our experiments (**Fig 4D**). By contrast, growth enhancements are not observed in simulated communities that lack an antibiotic-sensitive biotic inhibitor of the focal species (**Fig 4D**). This trend is also present in larger communities (**S10B Fig**). The focal species’ growth enhancement at subMICs increases with biotic inhibition and antibiotic susceptibility of the inhibitor species (**Fig 4E**). Overall, these model simulations suggest that the antibiotic sensitive inhibitor trend we observe with *C*. *difficile* in human gut communities is generalizable to other species and communities with variable richness, interaction networks, and antibiotic susceptibilities.

### *D*. *piger* substantially increases *C*. *difficile*’s minimum inhibitory concentration to metronidazole

Changes in antibiotic susceptibility of a pathogen due to significant microbial interactions could reduce the efficacy of antibiotic treatments. In the presence of *D*. *piger*, we observed a substantial increase in *C*. *difficile* MIC, in addition to a moderate growth enhancement of *C*. *difficile* at subMICs (**Fig 2B**). Consistent with the proposed antibiotic-sensitive biotic inhibition mechanism, *D*. *piger* was a weak biotic inhibitor of *C*. *difficile* and was more sensitive to metronidazole than *C*. *difficile* (**Figs 2A and 3C**). However, the substantial increase in *C*. *difficile* MIC in the presence versus absence of *D*. *piger* (≥24 μg/mL compared to 6 μg/mL) was not explained by the proposed antibiotic-sensitive biotic inhibition mechanism and the expanded gLV antibiotic model failed to predict this trend (**S8A Fig**). While competitive interactions (i.e., resource competition or biological warfare) contributed to the observed subMIC growth response, the mechanism of increased metronidazole MIC in the presence of *D*. *piger* was unknown.

**Fig 5:**
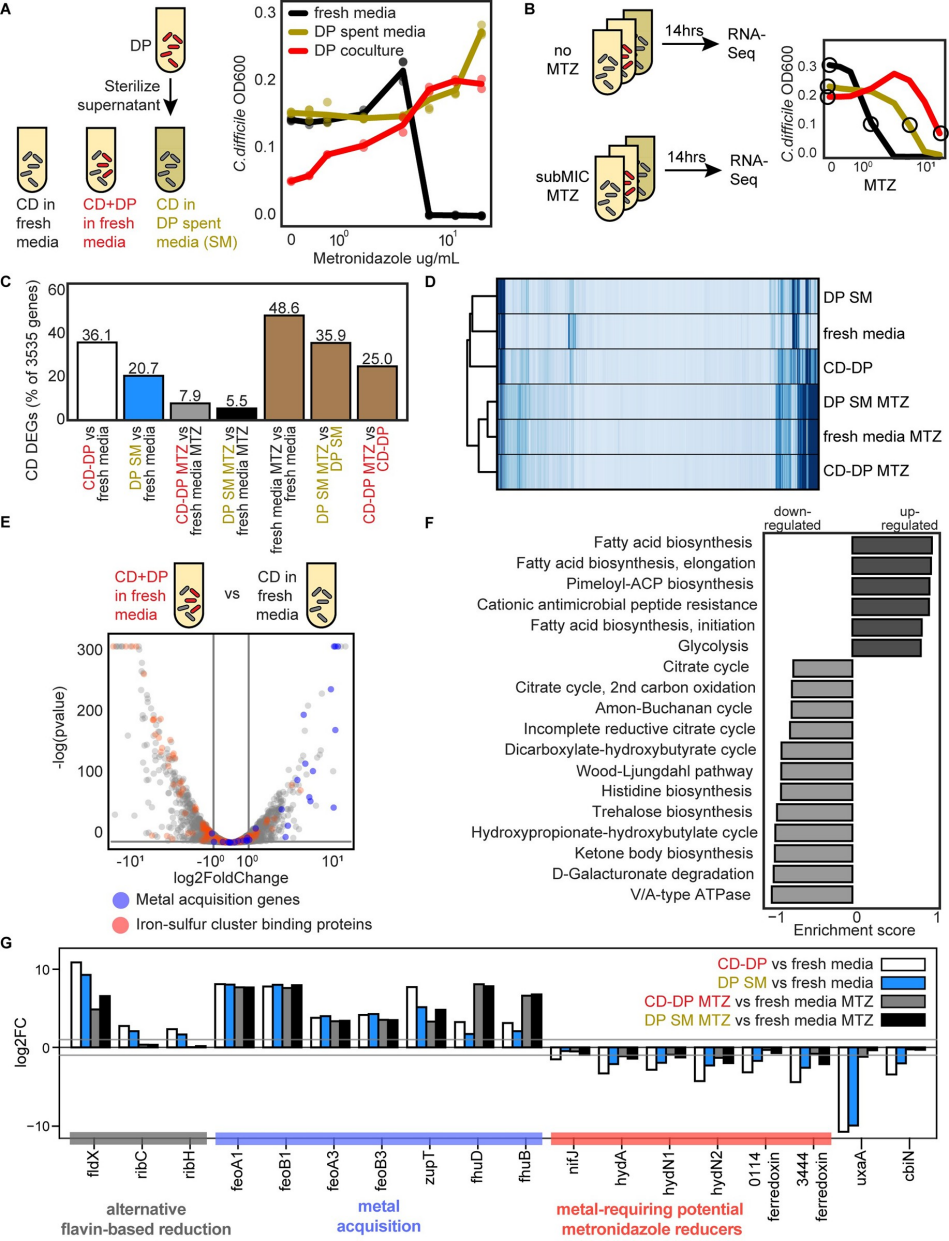
*D*. *piger* increases the metronidazole MIC of *C*. *difficile* and induces metal starvation genome-wide transcriptional response. **(A)** Abundance of *C*. *difficile* at 41 h in response to different metronidazole concentrations. The OD600 value for *C*. *difficile* in the presence of *D*. *piger* is calculated OD600 (OD600 multiplied by relative abundance from 16S rRNA gene sequencing). The x-axis is semi-log scale. Data points represent biological replicates. Lines indicate average of *n* = 2 to *n* = 4 biological replicates. **(B)** Schematic of genome-wide transcriptional profiling experiment. The x-axis is semi-log scale of metronidazole (MTZ) concentration. Lines indicate average *C*. *difficile* OD600 at 14 h for *n* = 4 biological replicates. Circles indicate conditions sampled for RNA-Seq. **(C)** *C*. *difficile* DEGs between culture conditions. DEGs are defined as genes that displayed greater than 2-fold change and a *p*-value less than 0.05. **(D)** Clustered heatmap of RPKM for each gene (rows) and in each sample (columns) for *C*. *difficile*. Each column represents the average of *n* = 2 biological replicates. Hierarchical clustering was performed based on Euclidean distance using the single linkage method of the Python SciPy clustering package. **(E)** Volcano plot of log transformed transcriptional fold changes for *C*. *difficile* in the presence of *D*. *piger*. Gray vertical lines indicate 2-fold change and the gray horizontal line indicates the statistical significance threshold (*p* = 0.05). Blue indicates genes annotated to be involved in metal import. Red indicates genes predicted to contain iron-sulfur clusters by MetalPredator [31]. **(F)** Enriched gene sets in *C*. *difficile* grown in the presence of *D*. *piger* compared to *C*. *difficile* grown in fresh media. All gene sets with significant enrichment scores from GSEA are shown. Gene sets are defined using modules from the KEGG. **(G)** Bar plot of the log transformed fold changes of a set of genes across different conditions. Gray horizontal lines indicate 2-fold change. The data underlying panels AB in this figure can be found in DOI: 10.5281/zenodo.7626486 and the data underlying panels CDEFG in this figure can be found in DOI 10.5281/zenodo.7049035 and 10.5281/zenodo.7049027 and S3 and S4 Tables. DEG, differentially expressed gene; GSEA, Gene Set Enrichment Analysis; KEGG, Kyoto Encyclopedia of Genes and Genomes; MIC, minimum inhibitory concentration; RPKM, reads per kilobase million.

We investigated the robustness of this trend across different environmental conditions and for different *C*. *difficile* isolates. *C*. *difficile* displayed an increased MIC in coculture with *D*. *piger* and in monoculture in *D*. *piger*’s spent media (≥8-fold increase in MIC) (**Fig 5A**). These data indicate that the increase in *C*. *difficile* metronidazole MIC was not dependent on cell-to-cell contact with *D*. *piger* or prior exposure of *D*. *piger* to metronidazole. *C*. *difficile* displayed a higher metronidazole MIC in *D*. *piger* spent media harvested at late exponential phase/early stationary phase than in spent media harvested at earlier time points in exponential phase (**S11A and S11B Fig**). Further, multiple *C*. *difficile* clinical isolates had an increased MIC in *D*. *piger* spent media (**S11C Fig**). Finally, *C*. *difficile* exhibited a higher metronidazole MIC in coculture with *D*. *piger* in a different chemically defined growth medium (**S11D Fig**). These data demonstrate that the protective effect of *D*. *piger* on *C*. *difficile’s* response to metronidazole was robust to variations in *C*. *difficile*’s strain background, media composition, and the growth phase of *D*. *piger*.

*D*. *piger* could alter *C*. *difficile’*s metronidazole MIC via a direct chemical interaction with metronidazole which reduces its efficacy (e.g., degradation or sequestration) or an indirect effect that modifies the activities of *C*. *difficile*’s intracellular networks which in turn yields an increase in metronidazole MIC. To test for a direct chemical interaction, we incubated metronidazole in either *D*. *piger* spent media or fresh media and characterized *C*. *difficile*’s growth response to each of these conditions in fresh media. *C*. *difficile*’s metronidazole MIC was equal in these conditions, indicating that compounds present in *D*. *piger*’s spent media do not directly interact with metronidazole to reduce its activity (**S11E Fig**).

### *C*. *difficile* displays a genome-wide transcriptional signature of metal limitation in response to *D*. *piger*

We considered indirect effects of *D*. *piger* on *C*. *difficile*’s intracellular network activities which in turn could alter *C*. *difficile*’s response to metronidazole. We first considered resource competition as a possible indirect effect because resource competition is a common mechanism driving interspecies interactions in microbial communities. *C*. *difficile*’s abundance was not substantially lower in *D*. *piger*’s spent media than monoculture (**Fig 5A**), suggesting that resource competition was not a major driver of the increased MIC. In addition, supplementing concentrated fresh media into the *D*. *piger* spent media, which would alleviate any resource competition, did not alter *C*. *difficile’s* metronidazole MIC (**S11F Fig**). Therefore, these data suggest that *D*. *piger* did not affect the metronidazole MIC of *C*. *difficile* via resource competition.

We considered if *D*. *piger* affected the metronidazole MIC of *C*. *difficile* by altering patterns in gene expression. We performed genome-wide transcriptomic profiling of *C*. *difficile* in late exponential phase in the following environments: (1) monoculture in fresh media; (2) monoculture in *D*. *piger* spent media; and (3) coculture with *D*. *piger*. Each of these cultures was exposed to a subMIC of metronidazole that reduced growth by approximately 50% or no treatment was applied (**Fig 5B**). The antibiotic concentration was chosen to allow sufficient growth for transcriptional profiling.

*C*. *difficile*’s gene expression profile in *D*. *piger* conditions (i.e., *D*. *piger* spent media and coculture with *D*. *piger*) was significantly altered compared to *C*. *difficile* monoculture (**Tables 1** and S3). Differentially expressed genes (DEGs) were defined as genes with >2-fold change and a *p*-value less than 0.05. In the absence of antibiotics, 36% and 21% of *C*. *difficile*’s 3,535 genes were differentially expressed in the *D*. *piger* coculture and spent media, respectively, compared to the *C*. *difficile* monoculture in fresh media (**Fig 5C**, white and blue bars). Of these genes, 72 in the *D*. *piger* coculture and 47 in the *D*. *piger* spent media exhibited >8-fold change, demonstrating that the presence of *D*. *piger* had a large impact on *C*. *difficile*’s gene expression profile. In the presence of metronidazole, a smaller percentage of genes were differentially expressed between the *D*. *piger* and fresh media conditions (8% and 6%, **Fig 5C**, gray and black bars). Large shifts in *C*. *difficile* gene expression occurred due to the addition of metronidazole, with 25% to 49% of genes differentially expressed (**Fig 5C**, brown bars). The 3 metronidazole conditions clustered closely together, indicating that the antibiotic induced similar changes in gene expression regardless of the media condition or ecological context (**Fig 5D**).

To provide deeper insights into specific genes that varied across conditions, we analyzed the genes with the largest expression changes in *D*. *piger* conditions. Many of the genes up-regulated in *D*. *piger* and *D*. *piger* antibiotic conditions compared to fresh media had annotated roles in metal acquisition (blue points in **Figs 5E** and **S12**). To acquire iron, *C*. *difficile* uses iron permeases to import free ferrous iron into the cell [32,33]. We observed >10-fold up-regulation in 2 ferrous transporters (*feoAB1*, *feoAB3*) in *D*. *piger* and *D*. *piger* metronidazole conditions (**Table 1**, **Fig 5G**). Alternative transporters were also up-regulated, such as uptake genes for ferrichrome siderophores (*fhuB*, *fhuD)* and catecholate siderophores (*CDR20291_1545–1548*, orthologs of *yclNOPQ* in CD630) as well as a zinc transporter gene (*zupT*). These genes (*feo*, *fhu*, *ycl*, and *zupT*) are regulated by the iron regulator *fur* [32] and have been shown to be up-regulated in response to iron starvation and zinc starvation [34–36], suggesting that metal limitation in *D*. *piger* conditions was responsible for the observed changes. Consistent with the metal limitation signature, ferrous transporters that were not up-regulated (*feoAB2*) have been documented as unresponsive to iron limitation [32,34].

**Table 1:** *C*. *difficile* gene expression in *D*. *piger* conditions compared to fresh media for genes with >16-fold differential expression.

			*D*. *piger* coculture vs. fresh media		*D*. *piger* spent media vs. fresh media	
**Locus_tag**	**Gene name**		**log2 FC**	**padj**	**log2 FC**	**padj**
CDR20291_1925	fldX	flavodoxin	10.86	0.0E+00	9.27	0.0E+00
CDR20291_2391	none	hypothetical protein	9.54	0.0E+00	8.57	9.0E-197
CDR20291_0517	none	putative membrane protein	8.73	0.0E+00	8.87	0.0E+00
CDR20291_0516	none	putative cation transporting ATPase	8.10	0.0E+00	8.36	0.0E+00
CDR20291_1327	feoA1	putative ferrous iron transport protein A	8.09	9.0E-167	8.03	3.0E-140
CDR20291_1328	feoB1	ferrous iron transport protein B	7.79	0.0E+00	8.00	0.0E+00
CDR20291_0946	zupT	zinc transporter	7.71	1.0E-51	5.14	3.0E-20
CDR20291_1329	none	putative exported protein	7.60	1.0E-105	7.49	1.0E-81
CDR20291_1326	none	putative ferrous iron transport protein	7.12	2.0E-227	6.96	2.0E-157
CDR20291_2009	none	putative Na(+)/H(+) antiporter	5.46	5.7E-221	3.93	1.0E-87
CDR20291_2825	none	ABC transporter, ATP-binding protein	5.30	1.6E-136	3.81	5.0E-51
CDR20291_2826	none	putative ABC transporter, permease protein	5.14	3.4E-130	3.58	4.0E-45
CDR20291_2827	none	hypothetical protein	4.91	7.4E-126	3.52	1.0E-44
CDR20291_0455	none	putative membrane protein	4.84	2.0E-54	1.94	5.0E-06
CDR20291_1545	none	putative iron compound ABC transporter, permease protein	4.66	9.0E-106	3.59	3.0E-46
CDR20291_1546	none	putative iron compound ABC transporter, permease protein	4.52	5.0E-61	3.40	4.0E-28
CDR20291_1547	none	putative iron compound ABC transporter, ATP-binding protein	4.46	4.0E-67	3.03	2.0E-23
CDR20291_0687	plfB	formate acetyltransferase	4.38	5.0E-203	2.51	5.0E-46
CDR20291_1021	acpP	acyl carrier protein	4.30	9.0E-101	2.00	9.0E-16
CDR20291_1548	none	putative iron compound ABC transporter, substrate-binding protein	4.23	8.0E-117	2.77	3.0E-28
CDR20291_2437	none	putative sugar transporter, substrate-binding lipoprotein	4.20	1.1E-165	2.16	3.0E-28
CDR20291_3135	feoB3	putative ferrous iron transport protein B	4.14	1.9E-189	4.26	2.0E-148
CDR20291_2824	none	ABC transporter, substrate-binding protein	4.11	9.9E-129	2.83	4.0E-40
CDR20291_1022	fabF	3-oxoacyl-[acyl-carrier-protein] synthase II	4.00	1.0E-121	1.58	2.0E-16
CDR20291_0363	none	Radical SAM-superfamily protein	−4.00	2.0E-134	−3.26	9.0E-71
CDR20291_0368	hadB	subunit of oxygen-sensitive 2-hydroxyisocaproyl-CoA dehydratase	−4.01	3.0E-183	−1.72	5.0E-25
CDR20291_2064	gabT	4-aminobutyrate aminotransferase	−4.04	3.0E-89	−3.07	4.0E-41
CDR20291_0364	none	putative membrane protein	−4.09	6.0E-164	−3.50	2.0E-78
CDR20291_3178	fdhD	formate dehydrogenase accessory protein	−4.09	8.0E-127	−2.45	4.0E-37
CDR20291_0523	cotJC1	putative spore-coat protein	−4.11	5.0E-107	−1.18	2.0E-09
CDR20291_0755	rbr	rubrerythrin	−4.11	2.0E-155	−2.40	2.0E-29
CDR20291_2227	abfH	NAD-dependent 4-hydroxybutyrate dehydrogenase	−4.12	3.9E-181	−2.00	4.0E-30
CDR20291_2509	none	putative membrane protein	−4.14	1.8E-164	−0.67	2.0E-04
CDR20291_0731	crt1	3-hydroxybutyryl-CoA dehydratase (crotonase)	−4.20	1.0E-08	−2.81	1.0E-05
CDR20291_2794	ntpI	V-type sodium ATP synthase subunit I	−4.27	3.2E-174	−1.79	8.0E-27
CDR20291_0177	none	putative oxidoreductase, NAD/FAD binding subunit	−4.29	1.0E-147	−3.29	2.0E-66
CDR20291_1478	none	hypothetical protein	−4.30	8.0E-122	−2.80	2.0E-39
CDR20291_3176	hydN2	electron transport protein	−4.31	2.7E-160	−2.32	4.0E-41
CDR20291_2566	ctfB	butyrate—acetoacetate CoA-transferase subunit B	−4.32	5.2E-11	−2.31	2.0E-05
CDR20291_0011	none	putative translation elongation factor	−4.33	2.0E-135	−1.72	1.0E-16
CDR20291_1511	none	hypothetical protein	−4.33	5.0E-128	−1.88	7.0E-22
CDR20291_0175	none	putative oxidoreductase, acetyl-CoA synthase subunit	−4.39	2.0E-183	−3.02	1.0E-59
CDR20291_2416	none	conserved hypothetical protein	−4.41	5.7E-102	−3.53	5.0E-59
CDR20291_2787	ntpD	V-type sodium ATP synthase subunit D	−4.41	3.9E-158	−2.18	1.0E-31
CDR20291_2228	abfT	4-hydroxybutyrate CoA transferase	−4.41	5.6E-189	−2.08	1.0E-32
CDR20291_0176	none	putative oxidoreductase, electron transfer subunit	−4.42	1.0E-181	−3.37	6.0E-61
CDR20291_3444	none	ferredoxin	−4.45	7.0E-182	−2.61	4.0E-25
CDR20291_2132	asrA	anaerobic sulfite reductase subunit A	−4.51	1.7E-10	−2.81	3.0E-06
CDR20291_1905	none	putative transcriptional regulator	−4.51	4.1E-52	−1.29	3.0E-07
CDR20291_2788	ntpB	V-type sodium ATP synthase subunit B	−4.56	5.8E-199	−1.91	1.0E-26
CDR20291_2800	adhE	aldehyde-alcohol dehydrogenase	−4.69	9.4E-15	−3.76	1.0E-09
CDR20291_0521	none	hypothetical protein	−4.71	6.0E-93	−1.15	5.0E-07
CDR20291_3177	none	hypothetical protein	−4.71	5.1E-59	−2.74	2.0E-25
CDR20291_2492	none	adp-ribosyltransferase binding component	−4.75	3.0E-208	−1.78	2.0E-20
CDR20291_2789	ntpA	V-type sodium ATP synthase subunit A	−4.75	1.4E-213	−2.15	3.0E-36
CDR20291_2491	none	cdta (adp-ribosyltransferase enzymatic component)	−4.79	9.4E-219	−1.66	5.0E-20
CDR20291_2792	ntpE	V-type sodium ATP synthase subunit E	−4.80	6.0E-145	−1.81	7.0E-23
CDR20291_1008	etfB3	electron transfer flavoprotein beta-subunit	−4.81	2.0E-92	−4.13	5.0E-60
CDR20291_0522	cotJB1	putative spore-coat protein	−4.83	4.0E-107	−1.38	3.0E-09
CDR20291_0728	none	putative hydroxymethylglutaryl-CoA lyase	−4.83	7.0E-14	−3.85	2.0E-10
CDR20291_2793	ntpK	V-type sodium ATP synthase subunit K	−4.91	1.2E-184	−1.78	4.0E-24
CDR20291_2790	ntpG	V-type sodium ATP synthase subunit G	−4.93	8.4E-144	−1.92	2.0E-21
CDR20291_2230	abfD	gamma-aminobutyrate metabolism dehydratase/isomerase	−4.96	4.5E-250	−2.15	4.0E-33
CDR20291_0709	none	conserved hypothetical protein	−4.99	2.0E-251	−3.38	1.0E-65
CDR20291_1007	none	conserved hypothetical protein	−5.03	2.0E-118	−4.47	1.0E-69
CDR20291_2229	none	conserved hypothetical protein	−5.09	9.9E-189	−2.74	3.0E-27
CDR20291_2791	ntpC	V-type sodium ATP synthase subunit C	−5.10	2.4E-183	−2.03	2.0E-27
CDR20291_0706	none	putative membrane protein	−5.20	9.0E-267	−3.43	4.0E-68
CDR20291_0191	none	putative membrane protein	−5.26	3.0E-254	−4.21	4.0E-127
CDR20291_0708	none	putative amidohydrolase	−5.32	2.0E-226	−3.72	2.0E-63
CDR20291_0707	none	putative membrane protein	−5.64	1.0E-286	−3.77	3.0E-87
CDR20291_0760	none	putative membrane protein	−5.72	3.0E-291	−4.08	2.0E-113
CDR20291_3092	none	conserved hypothetical protein	−6.15	0.0E+00	−4.44	4.0E-77
CDR20291_1689	none	hypothetical protein	−6.48	1.0E-39	−4.14	6.0E-31
CDR20291_2074	hcp	hydroxylamine reductase	−6.92	0.0E+00	−0.87	7.0E-06
CDR20291_2417	none	conserved hypothetical protein	−7.27	0.0E+00	−7.54	0.0E+00
CDR20291_1690	none	conserved hypothetical protein	−7.84	2.0E-158	−3.50	5.0E-68
CDR20291_2075	none	iron-sulfur binding protein	−8.29	0.0E+00	−1.62	7.0E-14
CDR20291_2419	none	putative aminotransferase	−9.21	0.0E+00	−8.46	0.0E+00
CDR20291_2418	none	putative membrane protein	−9.43	0.0E+00	−8.94	0.0E+00
CDR20291_2766	kdgT	2-keto-3-deoxygluconate permease	−10.13	0.0E+00	−9.66	0.0E+00
CDR20291_2768	uxaA	putative altronate hydrolase	−10.81	0.0E+00	−10.01	0.0E+00
CDR20291_1692	none	putative pyridine nucleotide-disulfide oxidoreductase	−11.08	0.0E+00	−2.96	9.0E-67
CDR20291_2767	uxaA’	altronate hydrolase (N-terminus)	−11.44	3.4E-41	−15.26	3.0E-21
CDR20291_1691	none	putative nitrite and sulfite reductase subunit	−12.01	0.0E+00	−3.97	9.0E-67

In addition to genes directly related to metal transport, many of the DEGs in *C*. *difficile* were linked to iron metabolism. Flavodoxin (*fldX*) was the most up-regulated gene in the *D*. *piger* conditions (**Table 1, Fig 5G**). Flavodoxin can replace iron-requiring electron transfer proteins (such as ferredoxin) in iron-limiting conditions [37]. Flavodoxin uses a nonmetal cofactor, flavin, whose biosynthesis genes (*ribACH*) were up-regulated in *D*. *piger* conditions (**Table 1**). Previous studies have shown that flavodoxin was regulated by *fur* and de-repressed in iron-limiting and zinc-limiting conditions [32,34–36], and riboflavin biosynthesis genes were up-regulated in iron-limiting conditions [35]. Iron-containing proteins have been shown to be down-regulated in iron-limited conditions [35]. Using a bioinformatic analysis, we found that 9% of *C*. *difficile’s* down-regulated genes (62 of 678 genes) in the *D*. *piger* coculture condition were predicted to contain iron-sulfur clusters (red points in **Fig 5E**, **S4 Table**). In sum, genes for the metal-independent flavodoxin were up-regulated, whereas transcripts for iron-requiring proteins were down-regulated, consistent with a metal-limited environment for *C*. *difficile* created by *D*. *piger*.

To identify other biological pathways that were significantly altered in *D*. *piger* conditions, we performed a gene set enrichment analysis (GSEA) using Kyoto Encyclopedia of Genes and Genomes (KEGG) modules (**Figs 5F** and **S12**). Many of the identified pathways could be connected to metals. For example, many of the enzymes in the down-regulated Wood–Ljungdahl pathway have iron-sulfur clusters, namely carbon monoxide dehydrogenase (*cooCS*, *CDR20291_0653*, *CDR20291_0655*) and formate dehydrogenase H (*fdhF*) [38–40]. In addition, the [NiFe] hydrogenase (*hydAN1N2*) in the Wood–Ljungdahl pathway contains a nickel-iron cluster. These enzymes have been shown to be down-regulated under iron-limited conditions [35]. Pathways for cationic antimicrobial peptide resistance and fatty acid biosynthesis were up-regulated, which has been previously attributed to iron limitation [35]. Similarly, the down-regulation of pathways for V-type ATPases, D-galacturonate degradation, and trehalose biosynthesis has been connected to iron limitation (**Fig 5F**) [35]. *D*. *piger* also affected the expression of *C*. *difficile* toxins (*tcdA*, *tcdB*, and binary toxin genes *CDR20291_2491* and *CDR20291_2492*) that were down-regulated between 4- and 27-fold in the *D*. *piger* conditions (**S3 Table**).

To provide further insights into the contribution of iron limitation on the patterns of gene expression in *C*. *difficile*, we evaluated the relationship between gene expression changes in *C*. *difficile* in the presence of *D*. *piger* and gene expression changes in *C*. *difficile* in iron-limited media previously characterized in a separate study [35]. For all DEGs, we compared the log2 fold changes between *C*. *difficile* in the *D*. *piger* coculture and *C*. *difficile* in monoculture in the absence of metronidazole with the log2 fold changes observed between *C*. *difficile* in iron-limited and iron-rich media in the previous study. These fold changes displayed an informative relationship and were qualitatively consistent for 89% of genes between the 2 studies (**S13A Fig**, Pearson r = 0.61, *p* = 8*10^−54^). Similarly, the fold changes of *C*. *difficile* cultured in *D*. *piger* spent media showed 87% qualitative agreement with the fold changes in the *C*. *difficile* iron-limitation study (**S13B Fig**). The informative relationship between these datasets suggests that the shifts in gene expression for the majority of *C*. *difficile*’s genes in the *D*. *piger* conditions can be explained by metal limitation.

Overall, these data suggest that *D*. *piger* created a metal-limited environment for *C*. *difficile*, as metal acquisition and alternative genes involved in flavin metabolism were up-regulated, whereas transcripts for metal-requiring proteins were down-regulated (**Fig 5G**). These trends were observed in both *D*. *piger* coculture and *D*. *piger* spent media and were also consistent with the trends in the *D*. *piger* conditions with metronidazole (**Fig 5G**).

### Differentially expressed genes in *C*. *difficile* in the presence of *D*. *piger* are linked to metronidazole resistance

Since the majority of *C*. *difficile’s* DEGs could be explained by metal limitation, we investigated the connection between metal limitation and *C*. *difficile* metronidazole MIC. To identify potential connections, we compared the set of DEGs in the presence of *D*. *piger* to genes previously shown to play a role in metronidazole resistance in *C*. *difficile*.

In 2 studies of metronidazole-resistant *C*. *difficile* mutants, iron-related genes were implicated in metronidazole resistance. In a study of *C*. *difficile* strain ATCC 700057, multiple mutants with increased metronidazole resistance acquired a truncation in *feoB1*, which resulted in reduced intracellular iron and a shift to flavodoxin [41]. Similarly, in a metronidazole-resistant mutant of a NAP1 strain, iron-uptake genes were down-regulated and a shift to flavodoxin was observed [42]. In each of these studies, the proposed mechanism of metronidazole resistance was attributed to down-regulation of enzymes predicted to reduce metronidazole to its active form. In the *D*. *piger* conditions, enzymes hypothesized to reduce metronidazole were also down-regulated, namely ferredoxin genes (*fdxA*, *CDR20291_0114*, *CDR20291_3444*), pyruvate-ferredoxin/flavodoxin oxidoreductase (PFOR) (*nifJ*), and hydrogenases (*hydA*, *hydN1*, *hydN2*) (**Table 1, Fig 5G**) [20,21]. Down-regulation of these enzymes in *D*. *piger* conditions may reduce the rate of conversion of metronidazole into its active form, thus increasing the tolerance of *C*. *difficile*. The down-regulated enzymes are each predicted to contain iron clusters (**S4 Table**), and hydrogenases are known to contain nickel cofactors [39], suggesting that their down-regulation and the subsequent decrease in metronidazole susceptibility could be attributed to metal limitation.

This mechanism of resistance has been proposed in other species beyond *C*. *difficile*. For example, *Bacteroides fragilis* metronidazole-resistant mutants displayed reduced PFOR expression [21]. Therefore, if this mechanism was responsible for the increase in *C*. *difficile*’s metronidazole MIC by *D*. *piger*, *Bacteroides* species should also display a higher MIC. Indeed, *B*. *thetaiotaomicron* displayed a higher metronidazole MIC in *D*. *piger* spent media compared to fresh media (**S14 Fig**).

We also identified that *cbiN*, a putative cobalt transporter that was down-regulated in the *D*. *piger* conditions (**Table 1, Fig 5G**) and in iron-limited media [35] has been previously implicated in metronidazole resistance. A single SNP present in *cbiN* distinguished a metronidazole-resistant R010 isolate of *C*. *difficile* from a metronidazole sensitive R010 isolate isolated from the same patient [43]. In our data, *cbiN* was down-regulated by 10-fold in the *D*. *piger* coculture and 4-fold in *D*. *piger* spent media.

Another enzyme that was down-regulated in the *D*. *piger* conditions, altronate hydrolase (*uxaA*), has potential connections with metronidazole resistance. Notably, altronate hydrolase was substantially down-regulated in the coculture with *D*. *piger*, where the magnitude of this decrease in transcript abundance was the second largest in the transcriptome (>1,000-fold reduction, **Table 1, Fig 5G**). Altronate hydrolase catalyzes the dehydration of the six-carbon altronate as part of galacturonate degradation [44]. One of the 17 mutations that distinguished a metronidazole-resistant NAP1 *C*. *difficile* strain from the metronidazole sensitive *C*. *difficile* reference strain occurred in the altronate hydrolase gene. The mutation in altronate hydrolase was one of 3 frameshift mutations in the mutant and likely rendered altronate hydrolase nonfunctional [45]. Studies in *E*. *coli* have demonstrated that this enzyme requires iron or manganese for its catalytic activity [46], consistent with its strong down-regulation in metal-limited media [35].

The mutations in *cbiN* and *uxaA* in metronidazole-resistant *C*. *difficile* isolates suggests that the observed down-regulation of *cbiN* and *uxaA* in response to metal limitation may contribute to *C*. *difficile’s* increased metronidazole MIC. These genes are potential links between metal limitation and metronidazole susceptibility, in addition to the down-regulation of metal-containing oxidoreductases, hydrogenases, and ferredoxins predicted to convert metronidazole into its active form.

### Hydrogen sulfide production by *D*. *piger* promotes metal sequestration

The global changes in *C*. *difficile*’s gene expression profile suggest that *D*. *piger* created a metal-limited environment. We hypothesized that these metal limitations could have been caused by hydrogen sulfide produced by *D*. *piger* [47]. In *D*. *piger* cultures, we observed a characteristic black precipitate (ferrous sulfide) that forms when iron combines with produced hydrogen sulfide [48]. Other divalent metals can precipitate with sulfide as well [49,50] and may be precipitating in addition to ferrous sulfide in the presence of *D*. *piger*.

**Fig 6:**
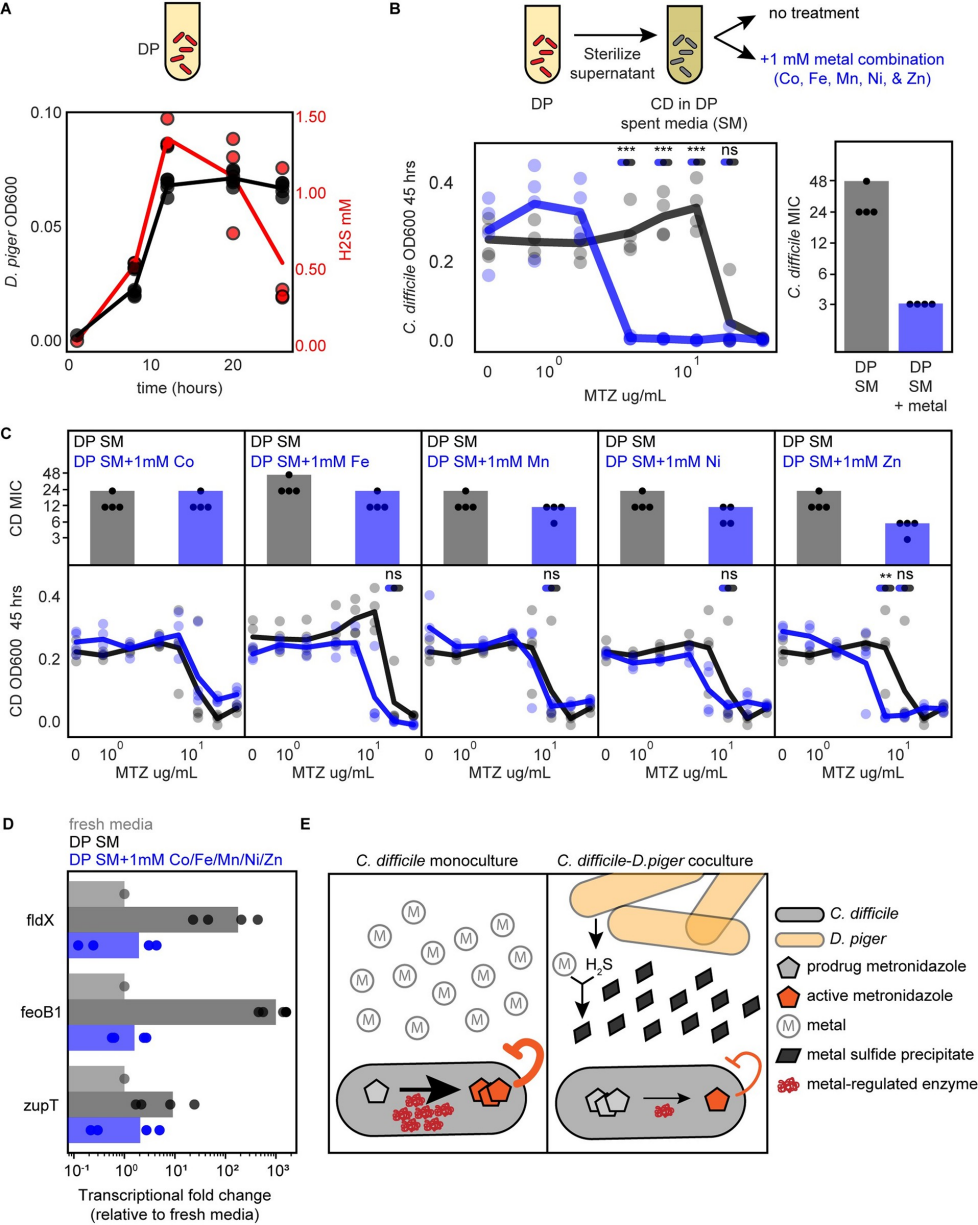
Supplementation of *D*. *piger* spent media with metals eliminates the protective effect on *C*. *difficile* in the presence of metronidazole. **(A)** Line plot of absolute abundance (black) and hydrogen sulfide production (red) of *D*. *piger* in monoculture. Data points represent biological replicates. Each biological replicate is the average of 2 technical replicates. Line indicates average of *n* = 4 biological replicates. **(B)** Line plot and bar plot of *C*. *difficile* metronidazole (MTZ) susceptibility in *D*. *piger* spent media (SM) with and without metal supplementation at 48 h. Metal supplementation condition contained 1 mM of Co, Mn, Ni, Zn, and Fe. The x-axis is semi-log scale (line plot). Data points represent biological replicates. Lines indicate average of *n* = 4 biological replicates. Asterisks indicate significant difference between conditions with and without metal supplementation (*P < 0.05, **P < 0.01, ***P < 0.001) according to an unpaired *t* test, “ns” indicates not significant. Statistical significance was performed at the lower of the 2 MICs and concentrations between the MICs of the 2 conditions. Bar plot displays MIC of data shown in line plot. Data points represent the MIC of *n* = 4 biological replicates. Bar represents the MIC determined based on average OD600 of *n* = 4 biological replicates. **(C)** Line plots and bar plots of *C*. *difficile* antibiotic susceptibility in *D*. *piger* spent media (SM) with and without supplementation of individual metals at 48 h. Each x-axis is semi-log scale. Data points represent biological replicates. Lines indicate the average of *n* = 4 biological replicates. Bar plot displays MIC of data shown in line plot. Data points represent MIC of *n* = 4 biological replicates. Bar represents the MIC determined based on an average OD600 of *n* = 4 biological replicates. Statistical significance as described in panel B. For metals with a change in MIC between the 2 conditions, statistical significance was tested at the lower of the 2 MICs and any concentrations between the MICs of the 2 conditions. **(D)** Bar plot of relative gene expression of 3 genes in *C*. *difficile* grown in fresh media, *D*. *piger* spent media (DP SM), and *D*. *piger* spent media with metal supplementation as detected by qRT-PCR. Fold change was calculated using the 2^−ΔΔCt^ method (Methods). Data points represent biological replicates, with each point calculated as the average of 3 technical replicates. Bar indicates the average of *n* = 4 biological replicates. Statistical significance was determined based on ΔCt values (S15 Fig). **(E)** Schematic of proposed mechanism for *D*. *piger* alteration of *C*. *difficile* metronidazole susceptibility. (Left) In monoculture, metals are available in the environment, and metal containing enzymes in *C*. *difficile* are expressed and reduce the prodrug metronidazole to its active form. (Right) In coculture with *D*. *piger*, hydrogen sulfide produced by *D*. *piger* sequesters metals, which forms metal sulfide precipitates. In response to metal limitation, the expression of metal binding proteins in *C*. *difficile* that reduce the conversion of metronidazole from prodrug to its active form is reduced. This in turn reduces the rate of conversion of metronidazole from its inactive to active form and increases the tolerance of *C*. *difficile*. The data underlying panels ABCD in this figure can be found in DOI: 10.5281/zenodo.7626486. MIC, minimum inhibitory concentration.

To estimate how much metal is precipitated by *D*. *piger* produced sulfide, we quantified the amount of sulfide in a monoculture of *D*. *piger* over time (Methods). The amount of sulfide peaked in late exponential phase at 1.4 mM (**Fig 6A**). Hydrogen sulfide is volatile and escapes during growth. Therefore, the total amount of produced hydrogen sulfide was likely higher than the measured concentration. While *C*. *difficile* also produces a small amount of hydrogen sulfide, the amount of hydrogen sulfide in the *C*. *difficile* and *D*. *piger* coculture was similar to the amount in the *D*. *piger* monoculture (**S15A Fig**). The produced hydrogen sulfide in the *D*. *piger* spent media and *D*. *piger* coculture (>1.4 mM) was in excess of the total divalent metal concentration in the media (1.2 mM iron and micromolar concentrations of other metals, **S15B Fig**). Therefore, the produced hydrogen sulfide could precipitate all divalent metals in the media, creating a metal-limited environment for *C*. *difficile*. This would explain the metal-limited signature in *C*. *difficile’s* gene expression profile. Supporting this hypothesis, iron depletion due to the precipitation with excess sulfide has been previously shown to induce Fur-regulated genes in *C*. *difficile* [51].

### Supplementation of *D*. *piger* spent media with combination of metals eliminates the protective effect of *C*. *difficile* from metronidazole

Based on the hypothesis that metal precipitation by hydrogen sulfide leads to an increase in *C*. *difficile*’s metronidazole MIC, we tested whether removing hydrogen sulfide from *D*. *piger* spent media eliminates this protective effect. The majority of hydrogen sulfide was eliminated from *D*. *piger* spent media by purging with nitrogen gas for 15 min (**S16A Fig**). We visually observed that nitrogen-purged spent media formed less ferrous sulfide precipitate than the untreated spent media (**S16B Fig**). Consistent with the proposed mechanism, the metronidazole MIC of *C*. *difficile* was reduced in the nitrogen-purged spent media compared to the untreated spent media (**S16C and S16D Fig**). This suggests that hydrogen sulfide contributed to the increase in *C*. *difficile* metronidazole MIC via metal precipitation.

Additionally, we hypothesized that supplementing *D*. *piger* spent media with metals that have precipitated would reduce the protective effect on *C*. *difficile*’s metronidazole MIC. We characterized *C*. *difficile*’s metronidazole MIC in media supplemented with cobalt, iron, manganese, nickel, and zinc since multiple divalent metals can form sulfide precipitates [49,50] and limitation of multiple divalent metals can lead to similar gene expression changes in *C*. *difficile* [35,36]. We introduced these metals in high concentrations (millimolar range) into the *D*. *piger* spent media to account for unreacted hydrogen sulfide that could precipitate the supplemented metals. Our results showed that *C*. *difficile’s* metronidazole MIC was substantially reduced in *D*. *piger* spent media supplemented with the five-metal combination compared to *D*. *piger* spent media without this addition (MIC decreased from 48 μg/mL to 3 μg/mL, **Fig 6B**). By contrast, *C*. *difficile*’s antibiotic MIC was not altered in fresh media supplemented with the five-metal combination, demonstrating that this effect was dependent on environmental modification by *D*. *piger* (**S17A Fig**). Supplementation of *D*. *piger* spent media with individual metals revealed that none of the individual metals decreased metronidazole MIC as substantially as the combination of metals (**Fig 6C**).

Consistent with these results, the expression of 3 genes that indicate metal limitation (*fldX*, *feoB1*, and *zupT*) was significantly reduced in *C*. *difficile* cultured in *D*. *piger* spent media supplemented with the metal combination compared to the spent media without metal supplementation (**Figs 6D and S17B**). Overall, this data suggests that metal limitation in *D*. *piger* spent media caused a global shift in *C*. *difficile*’s transcriptome and substantially reduced metronidazole susceptibility. Notably, both of these effects were eliminated by metal supplementation. Combining our data together, we propose a biological mechanism for the effect of *D*. *piger* on *C*. *difficile*’s metronidazole susceptibility (**Fig 6E**). In this proposed mechanism, hydrogen sulfide produced by *D*. *piger* sequesters divalent metals in the media. This in turn creates a metal limited environment for *C*. *difficile*, which leads to down-regulation of enzymes requiring metal cofactors for their activities. The down-regulated genes include enzymes that perform redox reactions that reduce metronidazole to its active form. Therefore, in coculture with *D*. *piger*, the activated concentration of metronidazole is reduced, yielding a substantially higher antibiotic MIC as a consequence of a reduction in DNA damage.

## Discussion

A fundamental question is uncovering the role of biotic interactions in shaping antibiotic susceptibility in microbiomes. In particular, understanding how microbial interactions alter the antibiotic susceptibility of major human pathogens such as *C*. *difficile* could enable tailored antibiotic treatments informed by ecological context. This understanding could inform new microbiome interventions that selectively eradicate human pathogens while minimizing disruption of healthy gut microbiota and minimize the acquisition of antibiotic resistance. We investigated the contribution of interspecies interactions to the antibiotic susceptibility of a major human gut pathogen *C*. *difficile*. We observed 2 types of alterations in *C*. *difficile* antibiotic susceptibility: changes in *C*. *difficile* MIC and changes in *C*. *difficile* abundance at subinhibitory concentrations. Substantial changes in *C*. *difficile* MIC were rare in our conditions, only occurring in a small fraction of communities (**S18A Fig**). By contrast, enhancements in the abundance of *C*. *difficile* at subMICs were more frequently observed, occurring in 52% of characterized communities (**S18B Fig**). Our work demonstrates that pathogen growth can be altered by interspecies interactions across a wide range of antibiotic concentrations, which should be considered in the design of antibiotic treatments.

We observed a ≥4-fold increase in *C*. *difficile*’s MIC compared to monospecies in 4% of pairwise communities (**Fig 2**) and 16% of multispecies communities in response to single antibiotics (**S1 and S2 Tables**). This is qualitatively consistent with the small fraction of interspecies interactions shown to modify susceptibility in other microbial communities [12–15]. While we focused on antibiotics used to treat *C*. *difficile*, future work could apply our approach to study the impact of gut microbes on the response of *C*. *difficile* to antibiotics that are risk factors for initiating *C*. *difficile* infections such as clindamycin, cephalosporins, and fluoroquinolones [52].

The observed increase in *C*. *difficile* metronidazole MIC in the presence of specific gut may contribute to the ineffectiveness of metronidazole in treating *C*. *difficile* infections in the human colon. Due to the low achieved metronidazole concentration in the human colon [53], even modest increases in *C*. *difficile* metronidazole MIC could allow the pathogen to survive. Based on our results, future testing of *C*. *difficile* antibiotic susceptibility could include conditions of *C*. *difficile* cultured with physiologically relevant microbial communities. For example, *C*. *difficile* could be cultured with a panel of resuspended fecal samples from multiple donors with disparate human gut microbiome compositions. Antibiotic treatments could be scored by balancing minimization of the disruption of healthy gut microbiota with minimization of the variability of *C*. *difficile’s* MIC across donor samples.

We propose a mechanism wherein *D*. *piger* depletes bioavailable divalent metals in the environment, resulting in transcriptional down-regulation of enzymes in *C*. *difficile* requiring metal cofactors. These enzymes are proposed to reduce metronidazole to its active form. This implies that a lower expression level of these enzymes protects *C*. *difficile* from the action of the antibiotic (**Fig 6E**). Iron limitation has been shown to increase microbial resistance to antibiotics with proton motive force (PMF)-dependent uptake due to the decrease in iron-sulfur containing complexes crucial for generating the PMF [54]. Because metronidazole passively diffuses into the cell independent of PMF [55], iron limitation likely impacts not the initial uptake rate of metronidazole but instead the conversion rate from prodrug to activated inhibitor. Our results are supported by previous studies that have identified a link between diminished intracellular iron levels and *C*. *difficile* metronidazole resistance [41,42]. Interestingly, heme limitation has previously been shown to sensitize *C*. *difficile* to metronidazole [56]. This is in contrast to the metal limitation induced metronidazole protection we observed here, suggesting a complex relationship between heme and non-heme iron and metronidazole susceptibility.

In our metal supplementation experiments, the cocktail of 5 metals caused a substantial decrease in *C*. *difficile* MIC, whereas the addition of individual metals, including iron alone, did not have this effect. We hypothesize that multiple metal cofactors are required for the enzymatic activities that contribute to metronidazole reduction. While some enzymes proposed to contribute to metronidazole reduction, such as PFOR, only require iron cofactors, others such as hydrogenase require both iron and nickel cofactors, supporting this hypothesis [20,21,39,57]. An alternative hypothesis is that iron is the major cofactor needed by enzymes that reduce metronidazole, but both iron and non-iron divalent metals are involved in the transcriptional regulation of these enzymes due to cross-regulation of iron and other divalent metals. A cross-regulation of the zinc and iron starvation responses has been demonstrated in *Staphylococcus aureus* [58].

Our proposed mechanism of metronidazole protection has implications beyond *D*. *piger* and *C*. *difficile*. There are multiple commensal species in addition to *D*. *piger* that can produce hydrogen sulfide [59]. These species may increase *C*. *difficile* metronidazole MIC through a similar mechanism. Beyond *C*. *difficile*, metronidazole is a widely used antibiotic to treat anaerobic bacterial infections [19], suggesting hydrogen sulfide producing bacteria could protect other pathogens from the action of metronidazole via divalent metal sequestration (**S14 Fig**).

Estimates of sulfide concentration in the colon (10^−3^ M range [60,61]) are higher than estimates of bioavailable iron (approximately 10^−3^ M based on fecal concentrations, with 30% estimated to be present in a bioavailable form [62,63]). The excess of sulfide suggests physiological concentrations of iron may be precipitated by sulfide in the colon. However, during *C*. *difficile* infection, iron concentrations vary significantly as damage of epithelial cells can release heme into the environment [64], leading to an increase in available iron. However, metal sequestration by the host immune system [65] could simultaneously reduce available iron. Thus, the effects of sulfide on bioavailable metal pools in mammalian gut infections are unknown. Future studies could investigate the effect of hydrogen sulfide on *C*. *difficile* metronidazole susceptibility in an infection model in the murine gut. If the mechanism persists in in vivo, reduction of dietary protein could be considered during metronidazole treatment, as lower dietary protein has been shown to reduce hydrogen sulfide production by gut microbiota [60,66].

We observed alterations in *C*. *difficile’s* subMIC response in approximately half of the characterized communities (**Fig 3B and 3E**). Our CFU experiments demonstrated that the growth enhancement in a subset of conditions could be explained by changes in *C*. *difficile* abundance (**S2D and S4 Figs**). This does not exclude the possibility that some fraction of the observed *C*. *difficile* growth enhancements based on the OD600 method could be impacted by changes in cell morphology. Nevertheless, the relationship between the degree of biotic inhibition by susceptible competitors and focal strain growth enhancements at subinhibitory antibiotic concentrations is consistent in our computational model. This model does not capture changes in cell morphology, yet is able to recapitulate the overall trends in *C*. *difficile* growth enhancements (**Fig 4**).

Our observed growth enhancement of *C*. *difficile* at subMICs is an example of the ecological principle of competitive release, where resistant species expand after removal of antibiotic-sensitive competitors [28]. Competitive release has been previously observed in a 10-member model community of a lung infection due to cystic fibrosis [29] and a 2-member and 4-member brewery community [14]. Here, we systematically studied competitive release in both pairwise interactions and multispecies communities and its impact on the growth of a major human gut pathogen *C*. *difficile*. We demonstrated that the magnitude of the focal strain’s growth enhancement depends on the degree of biotic inhibition (**Fig 3D and 3F**). In addition, we showed that resistant inhibitors can suppress the growth enhancement caused by antibiotic sensitive biotic inhibitors (**Figs 3D, 3F and S7A and S7B**). Additionally, our computational model identified general ecological principles driving subMIC growth enhancements, which can be applied to other antibiotic treated communities.

Our findings suggest that growth enhancement of pathogens could occur in high richness communities such as the human gut microbiome in the presence of subinhibitory concentrations of antibiotics in the absence of resistant inhibitors. Metronidazole and vancomycin concentrations in the colon are estimated based on fecal concentrations. Therefore, it is unknown whether subinhibitory concentrations of these antibiotics occur in the colon. Based on fecal concentrations, it has been suggested that *C*. *difficile* may encounter subinhibitory concentrations during metronidazole treatment [67]. To avoid growth enhancement of *C*. *difficile* or other pathogens at subinhibitory concentrations, antibiotic treatments could be combined with bacterial therapeutics consisting of cocktails of resistant biotic inhibitors to suppress pathogen growth enhancement. This benefit of resistant commensal species has been suggested previously [68]. Our data demonstrates that for vancomycin treatment, *Bacteroides thetaiotaomicron* and *Prevotella copri* are good candidates for such a cocktail as they are resistant biotic inhibitors that inhibit *C*. *difficile* in communities across all antibiotic concentrations (**S5A Fig**). Use of narrow spectrum antibiotics or selective bacteriophages [69] that specifically inhibit a pathogen could also reduce the risk of pathogen growth enhancement at subMICs by minimizing disruption of the biotic competitors.

Overall, we found 2 classes of interactions that yield enhanced *C*. *difficile* growth in the presence of antibiotics. These results suggest that the discovery of interactions that sensitize *C*. *difficile* to antibiotics may be challenging. Therefore, future ecologically informed therapeutic strategies could exploit the strong biotic competition of *C*. *difficile* by diverse human gut species [4] that are resistant to the antibiotics as opposed to interactions that sensitize *C*. *difficile* to antibiotics.

## Methods

### Strain and culturing information

Information on strains used in this study is found in S5 Table. Single use glycerol stocks for each strain were created as described previously [70]. All cultures were grown in anaerobic Basal Broth (ABB, Oxoid) in an anaerobic chamber with an atmosphere of 2.5 ± 0.5% H_2_, 15 ± 1% CO_2_ and balance N_2_ (Coy Lab products). Starter cultures were prepared by inoculating 100 μL of single use glycerol into 5 mL of ABB. *D*. *piger* starter cultures were supplemented with 28 mM sodium lactate (Sigma-Aldrich) and 2.7 mM magnesium sulfate (Sigma-Aldrich). *E*. *rectale* starter cultures were supplemented with 33 mM sodium acetate (Sigma-Aldrich). To ensure organisms were at similar growth stages at experimental set up, starter cultures were inoculated either 16 or 41 h prior, depending on the organism’s growth rate (see S5 Table). Cultures were incubated at 37°C with no shaking.

### Antibiotic titrations

Antibiotic susceptibility was determined following the Clinical and Laboratory Standards Institute protocol for antibiotic susceptibility of monospecies anaerobic organisms [26], with modifications to apply the method to multispecies communities. The protocol was modified to use broth microdilution method for all conditions, because some communities contained Bacteroides species for which broth microdilution method is recommended. The broth microdilution protocol was modified to use ABB media in order to support growth of all members of the community. Lastly, the protocol was modified to determine species absolute abundance as the product of optical density and relative abundance from 16S rRNA sequencing because our conditions contained multiple species.

Metronidazole (Sigma-Aldrich M1547) and vancomycin (Sigma-Aldrich V1130) stocks of 1 mg/mL were made in water, filter sterilized with 0.2 μM filters, and stored at −20°C as single use aliquots. Stocks were diluted in 2-fold dilution series at 10× the desired concentration. Cultures were inoculated into ABB in 96 deep well plates. Monospecies were inoculated with a starting OD600 of 0.0022. Pairs were inoculated with a starting OD600 of 0.00022 for *C*. *difficile* and 0.00198 for other species. Multispecies communities were inoculated with a total OD600 of 0.0066 with evenness of 1. Immediately after inoculation, the 10× antibiotic dilution series was diluted 1:10 into the communities. The plates were covered with gas-permeable seals (Breathe-Easy) and incubated at 37°C with no shaking.

After 48 h, cultures were mixed by pipetting and 200 μL aliquots were removed for sequencing and measuring OD600. Sequencing aliquots were spun down at 1,739 *g* for 15 min after which the supernatant was removed and the pellets were stored at −80°C. OD600 was measured for 2 dilutions of each sample and the dilution in the linear range of the instrument (Tecan Infinite Pro 200) was selected, and 16S rRNA sequencing was performed to determine relative abundances as described below. Species absolute abundance was calculated by multiplying the relative abundance of each species by the community OD600. The MIC was defined as the lowest concentration for which all concentrations greater to and equal restrict organism growth to significantly less (*p* < 0.05) than 0.05 OD600 or 5,000 CFU/mL as determined by a one-sample *t* test.

### Determination of community composition

DNA extraction, library preparation, and sequencing were performed as described previously [4]. Briefly, cell pellets were genome extracted using the Qiagen DNEasy protocol with gram-positive lysozyme pretreatment modified for 96-well plates. Genomic DNA was normalized to 2 ng/μL in water and the 16S v3-v4 region was amplified using dual-indexed primers arrayed in 96-well plates. Samples were cleaned with DNA Clean and Concentrator kit (Zymo) and sequenced on an Illumina MiSeq.

Sequencing data was analyzed as described previously [4]. Briefly, reads were demultiplexed with Basespace FastQ Generation, paired ends were merged with PEAR v0.90 [71], and mapped to a custom database of our species using the mothur v1.40.5 command classify.seqs with the Wang method with bootstrap cutoff value of 60% [72]. Relative abundance of an organism was calculated by dividing the number of reads mapped to that organism by the total number of reads for that sample. Absolute abundance was calculated by multiplying the relative abundance by the OD600 of that sample. Samples were removed from further analysis if >1% of the reads were mapped to species not expected to be in the sample (indicating contamination).

### *C*. *difficile* colony-forming unit counting

*C*. *difficile* selective plates were created by autoclaving *C*. *difficile* agar (Oxoid CM0601) and adding horse blood (Lampire 7233401, 70 mL/1L media), norfloxacin (Santa Cruz 215586, 120 μg/mL), moxalactam (Santa Cruz 250419, 320 μg/mL), and erythromycin (Santa Cruz 204742, 100 μg/mL) after media cooled to 55°C. After 48 h of incubation, cultures were mixed and diluted into PBS. Four dilutions of each sample were spotted on *C*. *difficile* selective agar plates, with 2 technical replicates per sample. Plates were incubated at 37°C for 48 h at which point colonies were counted in the dilution spot containing between 5 and 50 colonies. The CFU/mL for each sample was calculated as the average of the 2 technical replicates times the dilution factor. The lower limit of detection for the assay was 5,000 CFU/mL.

### Generalized Lotka–Volterra model with antibiotic perturbation

The antibiotic perturbation extension of the generalized Lotka–Volterra model [30] is a set of *N*-coupled first-order ordinary differential equations (Eq 1):

$$\frac{dX_{i}}{dt}=X_{i}\left(r_{i}+\sum_{j=1}^{N}\alpha_{ij}X_{j}+B_{i}a\right)$$

where *N* is the number of species, *X*_*i*_ is the abundance of species *i*, *r*_*i*_ is the basal growth rate of species *i*, *α*_*ij*_ is the growth modification of species *i* by species *j*, and *X*_*j*_ is the abundance of species *j*. The parameter *α*_*ij*_ is constrained to be negative when *i*  =  *j*, representing intraspecies competition. In the antibiotic term, *B*_*i*_ is the sensitivity of species *i* to antibiotic, and *a* is the concentration of the antibiotic. We modify the model to make the antibiotic concentration modified constant over time.

Values for growth rates parameters *r*_*i*_ and interaction parameters *a*_*ij*_ come from our previous work [4]. Values for antibiotic sensitivity parameters *B*_*i*_ were inferred in this study from time-series measurements of monospecies antibiotic titrations using a custom python script. The equation used to infer *B*_*i*_ from monospecies data was Eq 1, with *α*_*ij*_ = 0 for all j! = i. The minimize function of the Scipy optimize package was used to determine the optimal B_i_ for each species x_i_, that resulted in the lowest cost between the antibiotic gLV model and the time-series measurements across all the antibiotic concentrations in the titration. The antibiotic concentrations were scaled so that the concentrations range between 0 and 1. The cost in the optimization was computed by simulating the species abundance in each antibiotic condition with an ODE solver and summing the mean-squared error between the abundance of the species in the simulation and the abundance of the species in the data at each time point in each antibiotic concentration.

### Development of null computational models

Null model 1 (“gLV”) is the generalized Lotka–Volterra model. This is the same as the generalized Lotka–Volterra model with antibiotic perturbation (Eq 1) without the antibiotic term. This can also be thought of as the generalized Lotka–Volterra with antibiotic perturbation with all species antibiotic susceptibility set to zero (not susceptible). Null model 1 is Eq 1 with *B*_*i*_ = 0 for all species.

Null model 2 (“gLV + shuffled antib. suscept.”) is the generalized Lotka–Volterra model with antibiotic perturbation (Eq 1) with a shuffled set of antibiotic susceptibility parameters. For metronidazole conditions, the 14 metronidazole susceptibility parameters were shuffled and for the vancomycin conditions, the 14 vancomycin susceptibility parameters were shuffled (the shuffled *B*_*i*_ for species i is equal to the unshuffled *B* of any one of the 14 species). The accuracy of Null model 2 was averaged across 1,000 sets of shuffled parameters. The distance between the shuffled B for *C*. *difficile* and the unshuffled B for *C*. *difficile* was calculated as the absolute abundance of the difference between shuffled *B*_CD_ and unshuffled *B*_CD_.

Null model 3 (“gLV + monosp. Antib. Susceptibility, aijs = 0”) is the generalized Lotka–Volterra model with antibiotic perturbation (Eq 1) with no interspecies interactions. Null model 3 is Eq 1 with *α*_*ij*_ = 0 for all j! = i.

### Spent media preparation

*D*. *piger* starter culture was incubated for 48 h. The culture was then diluted into ABB to an OD600 of 0.0022. The cultures were incubated at 37°C with no shaking. Unless otherwise indicated, cultures were incubated for 12 h. After incubation, cultures were spun down at 1,739 *g* for 15 min. Supernatant was removed and filter sterilized with Steriflip 0.2 μM filters.

### Metronidazole incubation experiment

*D*. *piger* spent media was prepared following the procedure described above. An aliquot of metronidazole was incubated in *D*. *piger* spent media or rich media (ABB) at 37°C. After 12 h of incubation, the incubated metronidazole was diluted into a *C*. *difficile* culture in fresh media (ABB) following the antibiotic titration method described above.

### Iron-sulfur cluster prediction

The protein FASTA sequence from the *C*. *difficile* R20291 reference genome (GenBank assembly accession GCF_000027105.1) was run on the Metal Predator web-server [31].

### Metal quantification and supplementation

The amount of metal in fresh media (Anaerobic Basal Broth) was analyzed via Inductively Coupled Plasma Mass Spectroscopy by the Wisconsin State Lab of Hygiene.

In metal supplementation experiments, supernatants were supplemented with iron (II) sulfate (Alfa Aesar), manganese (II) sulfate monohydrate (Sigma-Aldrich), nickel sulfate (II) hexahydrate (Sigma-Aldrich), zinc (II) sulfate heptahydrate (Alfa Aesar), and/or cobalt (II) chloride hexahydrate (Sigma-Aldrich). Metal compounds were prepared as 100× stocks in water and filter sterilized with 0.2 μM filters.

### Hydrogen sulfide quantification

Hydrogen sulfide was quantified using the Cline Assay. Immediately before performing the assay, sodium sulfide (Alfa Aesar) was added to nitrogen purged water into a sealed vial. The stock was then diluted into 1% zinc acetate to 1 mM and further diluted into 1% zinc acetate to desired concentrations for a standard curve. Samples were removed from the anaerobic chamber and immediately diluted 5-fold into 1% zinc acetate. Cline reagent was prepared in advance (1.6 g N,N-dimethyl-p-phenylenediamine sulfate (Acros Organics), 2.4 g iron (III) chloride (Spectrum Chemical), 50 mL concentrated hydrochloric acid, 50 mL water, stored in the dark) of which 3 μL was added to the standards and samples. Samples were mixed by pipetting and incubated for 20 min in the dark before measuring absorbance at 670 nm. If the samples contained cells, after incubation the samples were spun down at 1,739 *g* for 10 min and the supernatant was transferred to a new plate to measure OD670.

### Whole-genome transcriptomics

*D*. *piger* spent media was prepared following the procedure described above. *C*. *difficile* and *D*. *piger* starter cultures were incubated for 48 h. *C*. *difficile* monoculture and CD-DP coculture conditions were inoculated from starter cultures into 96 deep well plates. For monoculture conditions, *C*. *difficile* was inoculated to an OD600 of 0.0022. For CD-DP coculture, *C*. *difficile* was inoculated to an OD600 of 0.0022 and *D*. *piger* was inoculated to an OD600 of 0.0198. Immediately after inoculation, antibiotics were added as a 1:10 dilution of 10× antibiotic stocks. The plates were covered with gas-permeable seals (BreathEasy) and were incubated at 37°C with no shaking. After 14 h, 800 μL of RNAprotect (Qiagen) was added to 400 μL of culture after which the cultures were mixed by pipetting and then incubated for 5 min at room temperature. Cultures were then centrifuged at room temperature for 10 min at 1,739 *g* and supernatant was carefully removed. Pellets were stored at −80°C.

RNA was extracted using acidic phenol bead-beating method. Pellets were resuspended in 500 μL 2× Buffer B (200 mM sodium chloride, 20 mM ethylenediaminetetraacetic acid) and transferred to 2 mL microcentrifuge tubes containing 500 μL Phenol:Chloroform:IAA (125:24:1, pH 4.5) and 210 μL 20% sodium dodecyl sulfate and were bead-beated with acid washed beads (Sigma G1277) for 3 min. All solutions were RNAse-free. Samples were centrifuged at 4°C for 5 min at 8,000 rpm, and 600 μL of the upper aqueous phase was added to 60 μL 3 M sodium acetate and 660 μL cold isopropanol and chilled on ice for 5 min before freezing for 5 min at −80°C. Samples were centrifuged at 4°C for 15 min at 18,200 g, the supernatant was decanted, and the pellet was washed with cold 100% ethanol. The pellets were dried in a biosafety cabinet for 10 min and then resuspended in 100 μL RNAse-free water. Samples were purified using RNeasy Mini Kit (Qiagen) and genomic DNA was removed using RNAse-Free DNase Set (Qiagen). Two replicates of each condition were sent to GENEWIZ (New Jersey, United States of America) for sequencing. GENEWIZ depleted rRNA with Ribozero rRNA Removal Kit (Illumina) before cDNA library preparation using NEBNext Ultra RNA Library Prep (NEB). GENEWIZ sequenced the libraries on Illumina HiSeq. Data was de-multiplexed using Illumina’s bcl2fastq 2.17 software, where one mismatch was allowed for index sequence identification.

The data was quality checked using FastQC. The BBDuk, BBSplit, and BBMap tools from BBTools suite were used to trim adapters, deplete rRNA, and map the remaining mRNA reads to the reference genomes (*C*. *difficile*: GenBank assembly accession GCF_000027105.1. *D*. *piger*: Genbank assembly accession GCA_000156375.1). FeatureCounts was used to map reads to features on the genome. Reads per kilobase million (RPKM) values were computed using a custom python script. The DESeq2 Bioconductor library v4.0.3 was used in R v4.0.4 to quantify differential gene expression using a negative binomial generalized linear models with apeglm shrinkage estimator. When calculating RPKM of *C*. *difficile* genes in the *C*. *difficile*-*D*. *piger* coculture, the “reads mapped” in the denominator was the number of reads mapped to the *C*. *difficile* genome. Similarly, when quantifying differential gene expression for *C*. *difficile* genes in the *C*. *difficile*-*D*. *piger* coculture, only reads mapped to the *C*. *difficile* genome were provided to DeSeq2.

### Real-time quantitative reverse transcription PCR (qRT-PCR)

*C*. *difficile* cultures were prepared the same as the transcriptomic samples and were incubated for the same duration (14 h). RNA was extracted using the same method as the transcriptomic samples and was purified using RNeasy Mini Kit (Qiagen). Genomic DNA was removed using RNAse-Free DNase Set (Qiagen). We performed cDNA synthesis with 1 ug of total RNA using the iScript Select cDNA Synthesis Kit (Bio-rad). We performed qRT-PCR (Bio-rad CFX Connect) with SsoAdvanced Universal SYBR Green SuperMix (Bio-rad). We used previously published primers for the *fldX*, *feoB1*, *zupT*, and *rrs* genes [32,73]. We computed the fold changes using the 2^−ΔΔCt^ method [74], where:

$$\Delta \Delta C_{t}=({Ct}_{sample,targ}-{Ct}_{sample,rrs})-({Ct}_{control,targ}-{Ct}_{control,rrs})$$

where “targ” refers to target gene fldX, feoB1, or zupT and *rrs* is the reference gene [73]. Sample refers to media type. Fresh media was used as control condition for Ct_control_. Each Ct value was the average of 3 technical replicates.

### Gene set enrichment analysis

GSEA was performed using the GSEA method of the ClusterProfiler R package (v4.2.2) [75]. KEGG modules for *C*. *difficile* R20291 (KEGG T number T00998) were used as gene sets and were supplied as a user defined annotation with the TERM2GENE field. The analysis was run with the log2FCs calculated by DeSeq2. The *p*-value cutoff used was 0.05 and the minimum gene set size used was 3.

## Supporting information

S1 Fig Monoculture growth responses of *C. difficile* and other commensal human gut microbes in the presence of metronidazole or vancomycin. **(A, B)** Line plots of monospecies OD600 across antibiotic concentrations for metronidazole (MTZ) and vancomycin (VAN) at 48 h. Each x-axis is semi-log scale. Colored data points indicate biological replicates. Colored lines indicate the average of *n* = 2 to *n* = 4 biological replicates. Bold number indicates MIC of data. Black lines indicate model fit. **(C)** Table of scaled antibiotic susceptibility parameter B for each species. The data underlying panels AB in this figure can be found in DOI: 10.5281/zenodo.7626486.
(TIF)

S2 Fig Characterization of *C. difficile* minimal inhibitory concentration by colony forming unit counting or 16S rRNA gene sequencing absolute abundance method. **(A)** Schematic of 2 methods for determining absolute abundance: calculated absorbance at 600 nm (OD600) or CFU counting. **(B, C)** Double axis line plots of absolute abundance at 48 h of *C*. *difficile* in the presence of (B) metronidazole (MTZ) and (C) vancomycin (VAN). Left axis (black) is calculated OD600 (OD600 multiplied by relative abundance from 16S sequencing) and right axis (red) is CFU per mL. The x-axis is semi-log scale. Black points indicate biological replicates. Red points indicate biological replicates, each the average of 2 technical replicates. Lines represent the mean of *n* = 4 biological replicates. Bold number indicates the MIC based on calculated OD600 (black) or CFU (red). “NG” indicates no growth above the MIC threshold for any conditions, and no MIC was able to be determined. Blue outlines indicate conditions for which the MIC was consistent between the 2 methods. Data collected on a separate day from Fig 2. (D) Heatmaps of *C*. *difficile* subMIC fold change in data from panels BC. Black indicates significant fold change (greater than 1) in at least 1 concentration, white indicates no significant fold change at any concentration. Significance was determined using unpaired *t* test between the abundance of *C*. *difficile* at a given subMIC and the abundance of *C*. *difficile* in the absence of antibiotic, *P* < 0.05. *C*. *difficile* abundance determined by CFU counting (left heatmap) or calculated OD600 method (right heatmap). The data underlying panels BCD in this figure can be found in DOI: 10.5281/zenodo.7626486.
(TIF)

S3 Fig Characterizing the growth response of pairwise human gut communities including *C. difficile* at antibiotic concentrations lower than the minimum inhibitory concentration (subMIC fold change). **(A, B)** Line plots of subMIC fold change for pairwise communities in metronidazole (MTZ) or vancomycin (VAN) at 48 h. SubMIC fold change is calculated as the species absolute abundance at the subMIC divided by the species absolute abundance in the absence of the antibiotic. Black horizontal line at y = 1 indicates no change in growth compared to the no antibiotic condition. Each x-axis is semi-log scale. Data points indicate biological replicates. Line represents mean of *n* = 3 to *n* = 8 biological replicates in panel A and *n* = 1 to *n* = 8 biological replicates in panel B. (C) Line plots of *C*. *difficile* monospecies over time. (Top) *C*. *difficile* OD600 at 0 μg/mL and 1.5 μg/mL metronidazole. (Bottom) *C*. *difficile* subMIC fold change μg/mL metronidazole. Gray horizontal line at y = 1 indicates no change in growth compared to no antibiotic condition. SubMIC fold change calculated as in panels AB. Points represent biological replicates. Line represents average of *n* = 4 biological replicates. Statistical significance analyzed for all time points where *C*. *difficile* OD600 was greater in the presence of antibiotic than the absence of antibiotic (“ns” *P* > 0.05, according to an unpaired *t* test). (D) Microscopy images of select *C*. *difficile* conditions from panel C: no antibiotic at 24 and 48 h and 1.5 μg/mL metronidazole at 48 h. Exponential phase *C*. *difficile* cells in presence of subMIC of metronidazole do not show morphological difference from exponential phase *C*. *difficile* cells grown in absence of antibiotic. (E) Line plots of *C*. *difficile* in pair with *Bacteroides thetaiotaomicron* over time. (Top) *C*. *difficile* OD600 in the presence of 0 μg/mL or 1.5 μg/mL metronidazole. (Bottom) *C*. *difficile* subMIC fold change in the presence of 1.5 μg/mL metronidazole. Gray horizontal line at y = 1 indicates no change in growth compared to no antibiotic condition. SubMIC fold change calculated as in panels AB. Points represent biological replicates. Line represents average of *n* = 4 biological replicates. Statistical significance analyzed for all time points where *C*. *difficile* OD600 was greater in the presence of antibiotic than the absence of antibiotic (“ns” *P* > 0.05, according to an unpaired *t* test). (F) Line plots of *C*. *difficile* in pair with *Clostridium hiranonis* over time. (Top) *C*. *difficile* OD600 in the presence of 0 μg/mL or 0.1875 μg/mL vancomycin. (Bottom) *C*. *difficile* subMIC fold change in the presence of 0.1875 μg/mL vancomycin. Gray horizontal line at y = 1 indicates no change in growth compared to the no antibiotic condition. SubMIC fold change calculated as in panels AB. Points represent biological replicates. Line represents average of *n* = 4 biological replicates. Statistical significance was analyzed for all time points where *C*. *difficile* OD600 was greater in the presence of antibiotic than the absence of antibiotic (*P < 0.05, **P < 0.01, ***P < 0.001, ns *P* > 0.05, according to an unpaired *t* test). (G) Box plot of maximum subMIC fold change for *C*. *difficile* in pairwise communities at 48 h. The maximum subMIC fold change is the maximum of the average subMIC fold change across all subMICs. The average subMIC fold change was calculated for each subMIC concentration by taking the average *C*. *difficile* absolute abundance at the subMIC divided by the average *C*. *difficile* absolute abundance in the no antibiotic condition (*n* = 1 to *n* = 4 biological replicates). *C*. *difficile* did not grow at any concentrations of vancomycin in CD-BO, CD-BT, CD-BU, and CD-PC pairs so these communities were not included in the analysis. Each data point represents 1 pairwise community. Gray horizontal line at y = 1 indicates no change in growth compared to the no antibiotic condition. Species are categorized as sensitive inhibitors if (1) the absolute abundance of *C*. *difficile* in a given pairwise community was significantly lower than the monoculture *C*. *difficile* abundance, in the absence of antibiotics, as determined by an unpaired *t* test; and (2) if the monospecies MIC was less than the monospecies MIC of *C*. *difficile*. Asterisks indicate significant difference (*P < 0.05, **P < 0.01, ***P < 0.001) according to a one-sided Mann–Whitney U test. The data underlying panels ABCEFG in this figure can be found in DOI: 10.5281/zenodo.7626486.
(TIF)

S4 Fig Absolute abundance of *C. difficile* in coculture with *B. vulgatus* using colony forming unit counting and 16S rRNA gene sequencing methods in the presence of metronidazole. Line plot of *C*. *difficile* abundance in presence of metronidazole in (black) monoculture or in (brown) coculture with *B*. *vulgatus*. Data points indicate biological replicates. Lines indicate the average of *n* = 4 biological replicates. Asterisks indicate significance between *C*. *difficile* CFU/mL at given concentration and *C*. *difficile* CFU/mL in the absence of antibiotic (*P < 0.05, **P < 0.01, ***P < 0.001, ns P > 0.05). The data underlying this figure can be found in DOI: 10.5281/zenodo.7626486.
(TIF)

S5 Fig Multispecies human gut communities in the presence of metronidazole or vancomycin. **(A, B)** Line plots of absolute abundance across A metronidazole (MTZ) and B vancomycin (VAN) concentrations at 48 h. Each x-axis is semi-log scale. Y-axis is calculated OD600 (OD600 multiplied by relative abundance based on 16S sequencing). Data points indicate biological replicates. Lines indicate the average of *n* = 1 to *n* = 4 biological replicates in panel A and *n* = 3 to *n* = 4 biological replicates in panel B. Color indicates species, see Fig 1C. Red borders indicate communities with ≥4-fold change in *C*. *difficile* MIC (see S1 Table). The data underlying all panels in this figure can be found in DOI: 10.5281/zenodo.7626486.
(TIF)

S6 Fig Growth response of *C. difficile* in multispecies communities in the presence of metronidazole or vancomycin. **(A, B)** Line plots of subMIC fold change in metronidazole (MTZ) or vancomycin (VAN) at 48 h. SubMIC fold change is calculated as the species absolute abundance in a given subMIC divided by the species absolute abundance in the absence of the antibiotic. Black horizontal line at y = 1 indicates no change in growth compared to no antibiotic condition. Each x-axis is semi-log scale. Data points indicate biological replicates. Lines indicate the average of *n* = 1 to *n* = 4 biological replicates in panel A and *n* = 3 to *n* = 4 biological replicates in panel B. Color indicates species, see Fig 1C. The data underlying all panels in this figure can be found in DOI: 10.5281/zenodo.7626486.
(TIF)

S7 Fig Analysis of the growth response of *C. difficile* in antibiotic concentrations lower than the minimum inhibitory concentration in multispecies communities in the presence of metronidazole or vancomycin. **(A)** Scatterplots of maximum *C*. *difficile* subMIC fold change in multispecies communities at 48 h as a function of the number (left) or abundance (right) of sensitive biotic inhibitors for both metronidazole and vancomycin. The x-axis represents the number of sensitive biotic inhibitors in a given community in the beginning of the experiment (left) or summed absolute abundance of sensitive biotic inhibitors in a given community at 48 h in the absence of the antibiotic (right). The average subMIC fold change was calculated for each subMIC concentration by computing the average *C*. *difficile* absolute abundance in a given subMIC divided by the average *C*. *difficile* absolute abundance in the absence of the antibiotic, average of *n* = 1 to *n* = 4 biological replicates. The maximum subMIC fold change (y-axis) is the maximum of the average subMIC fold change across all subMICs. The sensitive and resistant inhibitors are determined as in Fig 3F. Red points indicate communities where the sum of absolute abundance of resistant biotic inhibitors at 48 h was greater than 0.05 OD600. Spearman correlation annotated for all data points (black) and for only communities with resistant inhibitors >0.05 OD600 (red). **(B)** Box plot of the maximum subMIC fold change for *C*. *difficile* in multispecies communities. The maximum subMIC fold change (y-axis) is calculated as in panel A. Each data point represents 1 community. Community type (x-axis) determined as in Fig 3F. Gray horizontal line at y = 1 indicates no change in growth compared to the no antibiotic condition. Asterisks indicate a significant difference (*P < 0.05, **P < 0.01, ***P < 0.001) according to a one-sided Mann–Whitney U test. **(C)** Schematic of subMIC difference metric. The x-axis is semi-log scale. Gray horizontal line at y = 1 indicates no change in growth compared to the no antibiotic condition. **(D)** Scatterplots comparing the predicted value of the *C*. *difficile* subMIC difference in a given community (x-axis) with average experimental value of the *C*. *difficile* subMIC difference in the same community (y-axis). The predicted value was calculated by summing the *C*. *difficile* subMIC differences of each CD-SpX pairwise community for all SpX. SubMIC differences greater than 0 have subMIC fold changes greater than 1. SubMIC differences less than 0 have subMIC fold changes less than 1. Each point represents a given community at given concentration of metronidazole or vancomycin. Number of sensitive inhibitors (color) was determined as in Fig 3F. Black line indicates best fit linear regression for all data points. Gray y = x line represents a perfect prediction of community subMIC difference. Annotated Pearson correlation values are based on all data points. Annotated percentages indicate the percentage of total data points in a given quadrant. The data underlying panels ABD in this figure can be found in DOI: 10.5281/zenodo.7626486.
(TIF)

S8 Fig Predicting the growth of human gut microbes in pairwise communities using a modified generalized Lotka–Volterra ecological model. **(A, B)** Line plots of model prediction of absolute abundance of *C*. *difficile* and gut microbe pairwise communities at 48 h in presence of A metronidazole (MTZ) or B vancomycin (VAN). Each x-axis is semi-log scale. Y-axis is calculated OD600 (OD600 multiplied by relative abundance from 16S sequencing). Points indicate experimental data. Lines indicate model simulations. Color indicates species, see Fig 1C. Bold number is the sum of squared errors for *C*. *difficile* (square of the difference between model OD600 and the average experimental OD600, summed across all concentrations). The data underlying all panels in this figure can be found in DOI: 10.5281/zenodo.7626486.
(TIF)

S9 Fig Predicting the growth of human gut microbes in multispecies communities using a modified generalized Lotka–Volterra ecological model. **(A, B)** Line plots of model prediction of absolute abundance of species in multispecies communities at 48 h in the presence of A metronidazole (MTZ) or B vancomycin (VAN). Each x-axis is semi-log scale. The y-axis denotes calculated OD600 (OD600 multiplied by relative abundance from 16S sequencing). Data points indicate experimental data. Lines indicate model simulations. Color indicates species, see Fig 1C. Bold number is sum of squared errors for *C*. *difficile* (square of difference between model OD600 and average experimental OD600, summed across all concentrations). The data underlying all panels in this figure can be found in DOI: 10.5281/zenodo.7626486.
(TIF)

S10 Fig Predictions of the growth responses of *C. difficile* in the presence of antibiotic concentrations lower than the minimum inhibitory concentration using the modified generalized Lotka–Volterra model. **(A)** Antibiotic gLV model prediction of pairwise and multispecies community data in the presence of each antibiotic concentration. These predictions are summarized in the blue “gLV + monospecies antib susceptibility” bar in Fig 4B. **(B)** Simulated maximum subMIC fold change for a focal species cocultured with a 4 other species for 1,000 randomly generated parameter sets. All parameters were randomized between a set of bounds. Bounds: aji (−1.25, 1.25), growth rates (0, 1), intraspecies interactions (−1.25, 0), and antibiotic susceptibility (−6, 0). Color of the data point indicates the abundance of focal species in the community in the absence of antibiotics (where light colored data points indicate inhibition of a focal species). A community is classified as containing a susceptible inhibitor if for any non-focal species j, B_j_ < −0.1 and a_focal,j_ < −0.1. Gray horizontal line at y = 1 indicates no change in growth compared to the no antibiotic condition. The data and modeling scripts underlying all panels in this figure can be found in DOI: 10.5281/zenodo.7726490.
(TIF)

S11 Fig Robust protection of *C. difficile* from metronidazole in the spent media of *D. piger*. **(A)** Line plot of the growth curve of *D*. *piger*. Colored data points indicate the time at which 5 separate *D*. *piger* spent media samples were harvested. Data points represent biological replicates. The line represents the mean of *n* = 5 biological replicates. **(B)** Line plot of *C*. *difficile* abundance at 41 h in 5 different *D*. *piger* spent medias in the presence of metronidazole (MTZ). The x-axis is semi-log scale. Data points indicate biological replicates. Line represents the mean of *n* = 4 biological replicates. **(C)** Line plot of the abundance of 4 clinical *C*. *difficile* isolates in the presence of metronidazole. Each x-axis is semi-log scale. The y-axis denotes the integral of *C*. *difficile* OD600 from 0 to 48 h. Data points indicate biological replicates. The line represents the mean of *n* = 3 biological replicates. Asterisks indicate a significant difference (*P < 0.05, **P < 0.01, ***P < 0.001) according to an unpaired *t* test. **(D)** Line plot of *C*. *difficile* abundance at 48 h in a rich chemically defined media (“DM38”) in the presence of metronidazole. The x-axis is semi-log scale. For the *D*. *piger* coculture condition, OD600 denotes calculated OD600 (OD600 multiplied by relative abundance from 16S rRNA gene sequencing). Data points indicate biological replicates. The line represents the mean of *n* = 6 (fresh media) or *n* = 3 (DP SM) biological replicates. Asterisks indicate a significant difference (*P < 0.05, **P < 0.01, ***P < 0.001) according to an unpaired *t* test. **(E)** Line plot of *C*. *difficile* abundance at 48 h in fresh media in the presence of metronidazole that was incubated in fresh media or *D*. *piger* spent media. The x-axis is semi-log scale. Data points indicate biological replicates. Line represents the mean of *n* = 4 biological replicates. No significant difference (“ns”, *p* > 0.05) according to an unpaired *t* test. **(F)** Line plot of *C*. *difficile* abundance at 48 h in fresh media, *D*. *piger* spent media, or *D*. *piger* spent media with a fresh media spike. The x-axis is semi-log scale. Data points indicate biological replicates. The line represents the mean of *n* = 4 biological replicates. Asterisks indicate significant difference (*P < 0.05, **P < 0.01, ***P < 0.001, “ns” *P* > 0.05) according to an unpaired *t* test. The data underlying panels ABD in this figure can be found in DOI: 10.5281/zenodo.7626486.
(TIF)

S12 Fig Changes in transcriptional abundance of metal-related genes or genes in key metabolic pathways in C. difficile. **(A–E)** Left: Schematic of 2 conditions being compared. Middle: Volcano plot of the fold change in *C*. *difficile* transcript abundance. Gray vertical lines indicate 2-fold change (1 in log2) and gray horizontal line indicates statistical significance (*p* = 0.05). Blue indicates genes annotated to be involved in metal import. Red indicates genes predicted to contain iron-sulfur clusters by MetalPredator. Right: Enriched KEGG modules in *C*. *difficile*. All KEGG pathways with significant enrichment scores from Gene Set Enrichment Analysis (GSEA) are shown. The data underlying all panels in this figure can be found in S3 and S4 Tables.
(TIF)

S13 Fig Relationship between the change in transcriptional abundance of *C. difficile* in coculture with *D. piger* versus fresh media and the change in transcriptional abundance in the presence of low versus high iron media in Berges and colleagues. **(A, B)** Scatterplots to evaluate the patterns in gene expression for gene orthologs between CDR20291 (this study) and CD630Δerm (Berges and colleagues [35]). Genes only shown if differentially expressed in both studies. X-axis: log2 fold change between low iron (0.2 μM) and high iron (15 μM) media in Berges and colleagues [35]. Y-axis: log2 fold change between *C*. *difficile* in coculture with *D*. *piger* (panel A) or *C*. *difficile* in *D*. *piger* spent media (SM) (panel B) and *C*. *difficile* in fresh media in this study. Each data point indicates a gene. Percentages indicate percentage of genes in each quadrant. Black line indicates best fit linear regression for all data points. Gray line is y = x. The data underlying all panels in this figure can be found in S3 Table.
(TIF)

S14 Fig *B. thetaiotaomicron* displays a higher minimum inhibitory concentration in supernatant of *D. piger* than in fresh media in the presence of metronidazole. Line plot of *B*. *thetaiotaomicron* abundance at 48 h in fresh media or in 75% *D*. *piger* spent media, 25% fresh media. The x-axis is semi-log scale. Data points represent biological replicates. Lines indicate the average of *n* = 3 biological replicates. The data underlying this figure can be found in DOI: 10.5281/zenodo.7626486.
(TIF)

S15 Fig Concentrations of hydrogen sulfide and divalent transition metals. **(A)** Bar plot of the amount of hydrogen sulfide in multiple conditions after 10 h of incubation. “Blank” represents a fresh media control. The y-axis denotes the percentage of the amount of hydrogen sulfide in the *D*. *piger* monoculture. Data points represent the mean of *n* = 2 technical replicates. Bar represents the mean of *n* = 8 biological replicates. Asterisks indicate significant difference (*P < 0.05, **P < 0.01, ***P < 0.001, “ns” *P* > 0.05) according to an unpaired *t* test. **(B)** Bar plot of the concentration of divalent transition metals in fresh media. Points represent the mean of *n* = 2 technical replicates. Bar represents the mean of *n* = 3 biological replicates. The data underlying all panels in this figure can be found in DOI: 10.5281/zenodo.7626486.
(TIF)

S16 Fig Nitrogen purge of *D. piger* spent media reduces hydrogen sulfide concentration, metal precipitates, and *C. difficile* metronidazole minimum inhibitory concentration. **(A)** Bar plot of hydrogen sulfide concentration in different medias. Data points represent *n* = 4 biological replicates. Bar represents the average of biological replicates. **(B)** Image of *D*. *piger* spent media 2 h after nitrogen purge (right) or no treatment (left). Precipitation of a black ferrous sulfide turns the media a darker color. **(C)** Line plot of *C*. *difficile* OD600 at 41 h in different medias in the presence of metronidazole. The x-axis is semi-log scale. Data points represent biological replicates. Lines indicate the average of *n* = 4 to *n* = 8 biological replicates. **(D)** Bar plot displaying MIC of data shown in C. Data points represent the MIC of *n* = 4 to *n* = 8 biological replicates. Bar represents the MIC determined based on an average OD600 of *n* = 4 to *n* = 8 biological replicates. The data underlying panels ACD in this figure can be found in DOI: 10.5281/zenodo.7626486.
(TIF)

S17 Fig Supplementation of fresh media with metals does not substantially alter *C. difficile*’s minimal inhibitory concentration to metronidazole. **(A)** Line plots and bar plots of *C*. *difficile* metronidazole susceptibility in fresh media with and without metal supplementation. (Top) Bar plots of *C*. *difficile* metronidazole MIC in untreated fresh media (gray) and media with 1 mM metal supplementation (blue). In the metal combination condition (“Combo”), all 5 metals were supplemented at 1 mM. Data points represent the MIC of *n* = 4 biological replicates. Bar represents the MIC determined based on an average OD600 of *n* = 4 biological replicates. (Bottom) Line plots of *C*. *difficile* OD600 at 45 h in untreated fresh media (gray) or media supplemented with 1 mM metal (blue) in the presence of metronidazole (MTZ). Each x-axis is semi-log scale. Data points represent biological replicates. Lines indicate the average of *n* = 4 biological replicates. **(B)** Bar plot of delta Ct (ΔCt) values of 3 genes in *C*. *difficile* grown in fresh media, *D*. *piger* spent media (DP SM), or *D*. *piger* spent media with metal supplementation based on qRT-PCR. Data points represent biological replicates, with each point calculated as the average of 3 technical replicates. Bar indicates the average of *n* = 4 biological replicates. Asterisks indicate significant difference (*P < 0.05, **P < 0.01, ***P < 0.001, “ns” *P* > 0.05) according to an unpaired *t* test. The data underlying all panels in this figure can be found in DOI: 10.5281/zenodo.7626486.
(TIF)

S18 Fig Summary of *C. difficile*’s antibiotic response in both pairwise and multispecies communities. **(A)** Pie charts of the change in *C*. *difficile* MIC in pairwise and multispecies communities compared to monoculture. **(B)** Pie charts of the growth enhancement of *C*. *difficile* in pairwise and multispecies communities. Growth enhancement is defined as a maximum subMIC fold change that is greater than 1 and is greater than the maximum subMIC fold change in *C*. *difficile* monoculture for that antibiotic (i.e., greater than 1.61 for metronidazole). The data underlying all panels in this figure can be found in DOI: 10.5281/zenodo.7626486.
(TIF)

S1 Table *C. difficile* metronidazole MICs in multispecies communities. MICs in μg/mL. *C*. *difficile* monoculture MIC from same experimental day given for comparison, as monoculture MIC can vary due to preculture conditions and glycerol stock batches.
(XLSX)

S2 Table *C. difficile* vancomycin MICs in multispecies communities. MICs in μg/mL. *C*. *difficile* monoculture MIC from same experimental day given for comparison, as monoculture MIC can vary due to preculture conditions and glycerol stock batches. “No growth” represents no growth of *C*. *difficile* across all concentrations and therefore no MIC could be determined.
(XLSX)

S3 Table *C. difficile* gene expression changes between conditions. (XLSX)

S4 Table *C. difficile* proteins predicted to contain iron-sulfur clusters by MetalPredator. (XLSX)

S5 Table Strain information. (XLSX)

## Abbreviations

CFU: colony-forming unit
DEG: differentially expressed gene
GSEA: Gene Set Enrichment Analysis
KEGG: Kyoto Encyclopedia of Genes and Genomes
MIC: minimum inhibitory concentration
PFOR: pyruvate ferredoxin/flavodoxin oxidoreductase
PMF: proton motive force
RPKM: reads per kilobase million
subMIC: sub-minimum inhibitory concentration
