# Supplementary figures and images for: Gut microbiota interspecies interactions shape the response of Clostridioides difficile to clinically relevant antibiotics
Source: PLoS Biol. 2023 May 11;21(5):e3002100. doi: 10.1371/journal.pbio.3002100 (PMC10174544; doi:10.1371/journal.pbio.3002100)

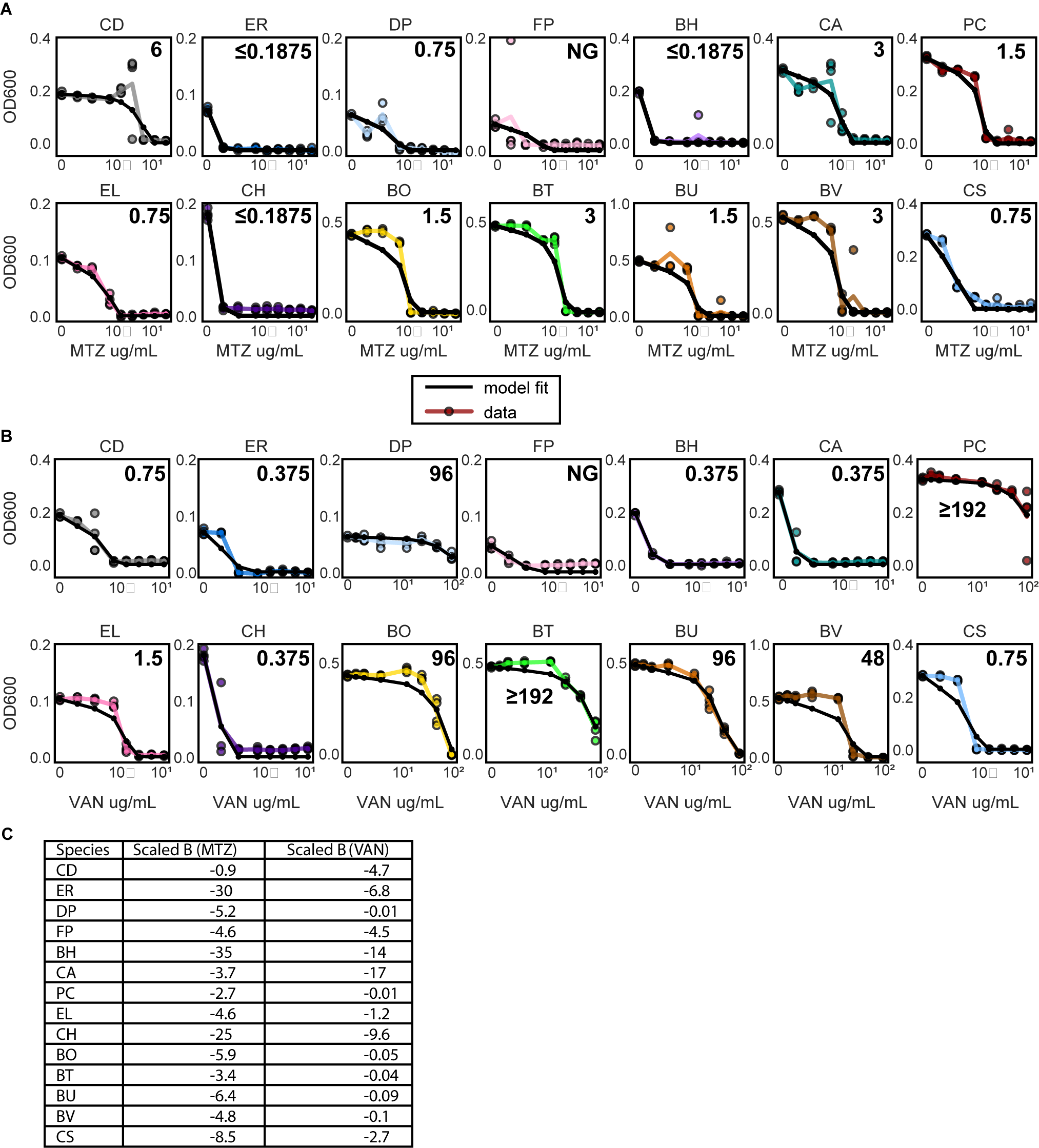

Supplement: S1 Fig — (A, B) Line plots of monospecies OD600 across antibiotic concentrations for metronidazole (MTZ) and vancomycin (VAN) at 48 h. Each x-axis is semi-log scale. Colored data points indicate biological replicates. Colored lines indicate the average of n = 2 to n = 4 biological replicates. Bold number indicates MIC of data. Black lines indicate model fit. (C) Table of scaled antibiotic susceptibility parameter B for each species. The data underlying panels AB in this figure can be found in DOI: 10.5281/zenodo.7626486. (TIF) [file pbio.3002100.s001.tif]

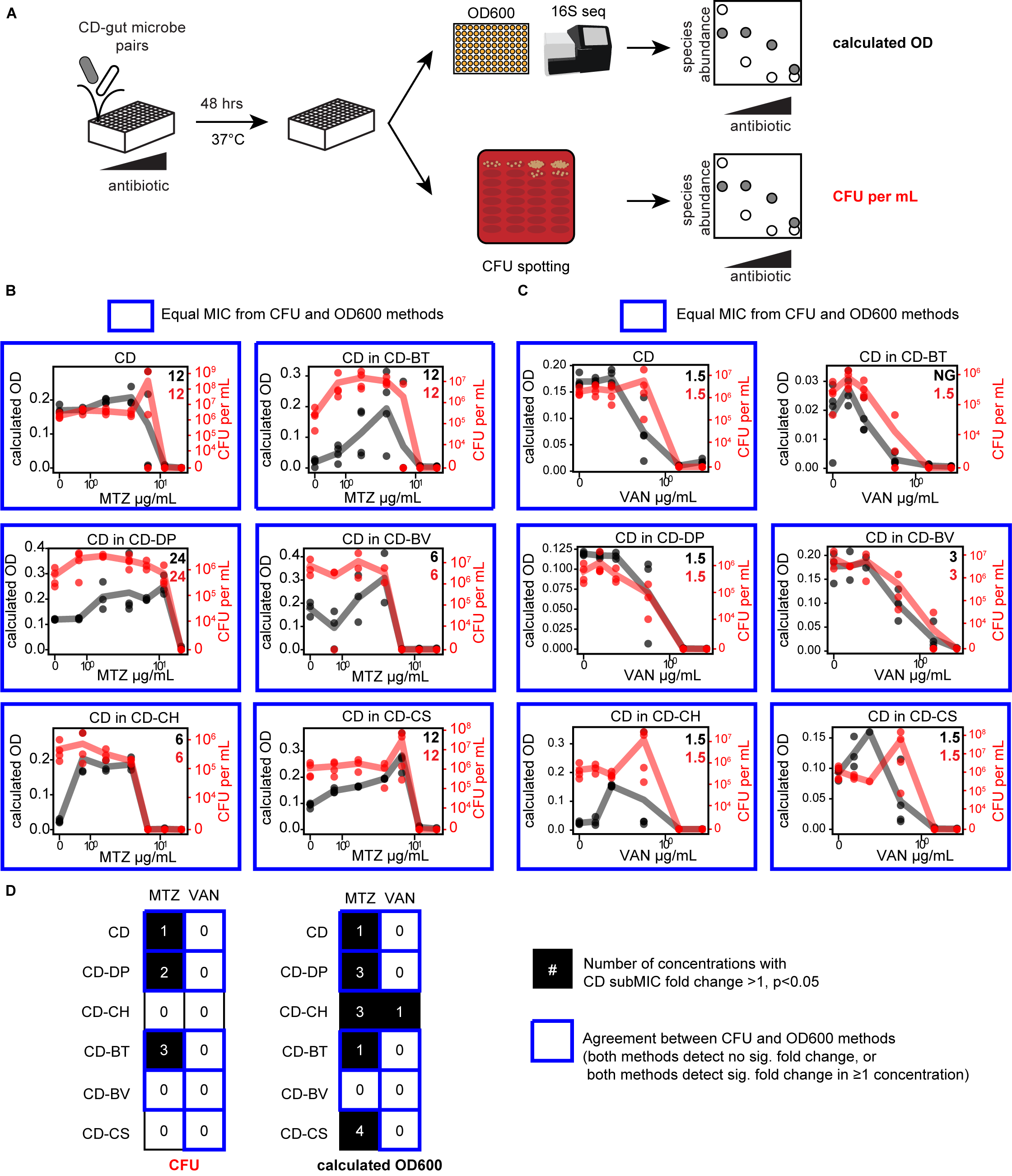

Supplement: S2 Fig — (A) Schematic of 2 methods for determining absolute abundance: calculated absorbance at 600 nm (OD600) or CFU counting. (B, C) Double axis line plots of absolute abundance at 48 h of C. difficile in the presence of (B) metronidazole (MTZ) and (C) vancomycin (VAN). Left axis (black) is calculated OD600 (OD600 multiplied by relative abundance from 16S sequencing) and right axis (red) is CFU per mL. The x-axis is semi-log scale. Black points indicate biological replicates. Red points indicate biological replicates, each the average of 2 technical replicates. Lines represent the mean of n = 4 biological replicates. Bold number indicates the MIC based on calculated OD600 (black) or CFU (red). “NG” indicates no growth above the MIC threshold for any conditions, and no MIC was able to be determined. Blue outlines indicate conditions for which the MIC was consistent between the 2 methods. Data collected on a separate day from Fig 2. (D) Heatmaps of C. difficile subMIC fold change in data from panels BC. Black indicates significant fold change (greater than 1) in at least 1 concentration, white indicates no significant fold change at any concentration. Significance was determined using unpaired t test between the abundance of C. difficile at a given subMIC and the abundance of C. difficile in the absence of antibiotic, P < 0.05. C. difficile abundance determined by CFU counting (left heatmap) or calculated OD600 method (right heatmap). The data underlying panels BCD in this figure can be found in DOI: 10.5281/zenodo.7626486. (TIF) [file pbio.3002100.s002.tif]

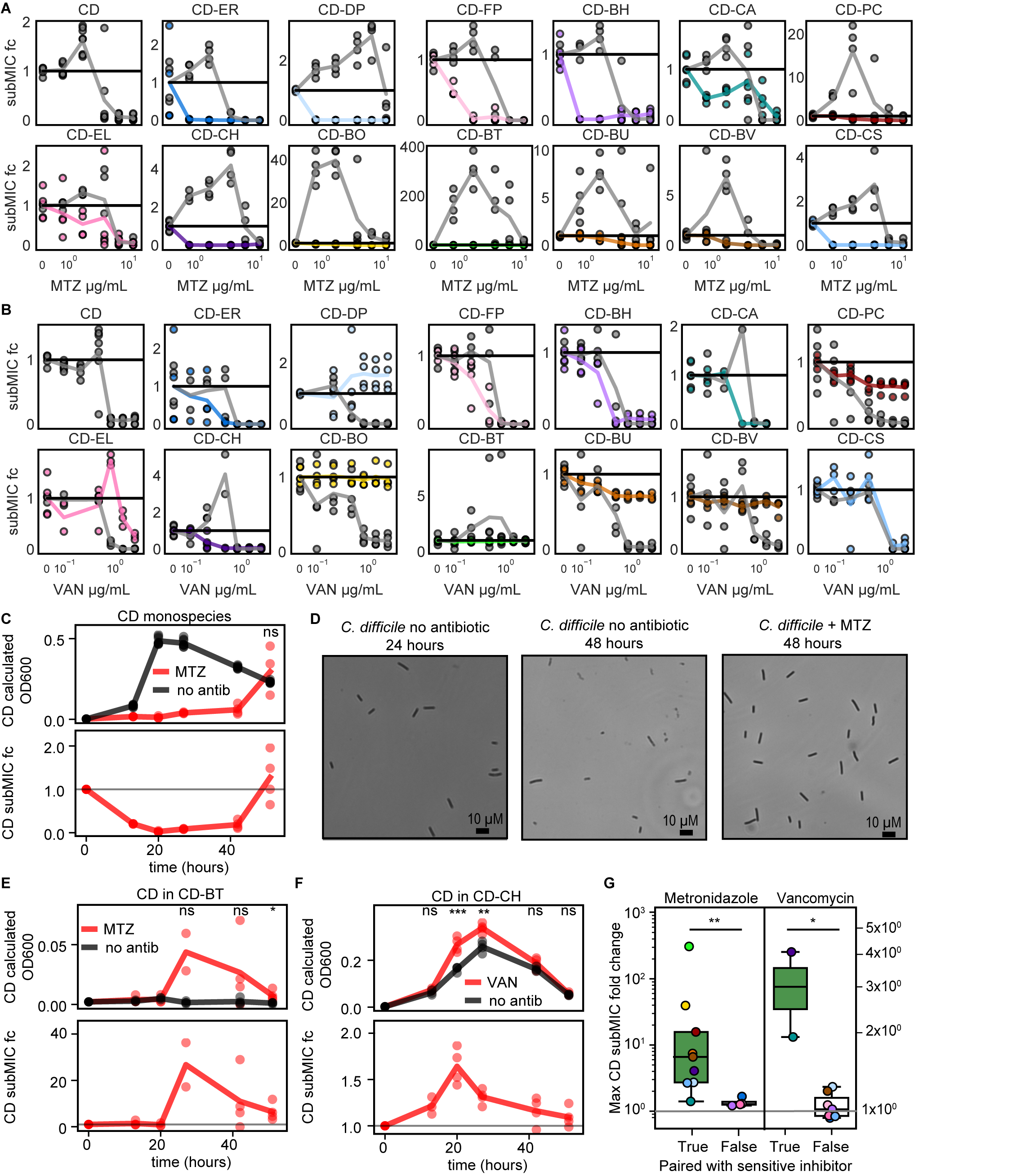

Supplement: S3 Fig — (A, B) Line plots of subMIC fold change for pairwise communities in metronidazole (MTZ) or vancomycin (VAN) at 48 h. SubMIC fold change is calculated as the species absolute abundance at the subMIC divided by the species absolute abundance in the absence of the antibiotic. Black horizontal line at y = 1 indicates no change in growth compared to the no antibiotic condition. Each x-axis is semi-log scale. Data points indicate biological replicates. Line represents mean of n = 3 to n = 8 biological replicates in panel A and n = 1 to n = 8 biological replicates in panel B. (C) Line plots of C. difficile monospecies over time. (Top) C. difficile OD600 at 0 μg/mL and 1.5 μg/mL metronidazole. (Bottom) C. difficile subMIC fold change μg/mL metronidazole. Gray horizontal line at y = 1 indicates no change in growth compared to no antibiotic condition. SubMIC fold change calculated as in panels AB. Points represent biological replicates. Line represents average of n = 4 biological replicates. Statistical significance analyzed for all time points where C. difficile OD600 was greater in the presence of antibiotic than the absence of antibiotic (“ns” P > 0.05, according to an unpaired t test). (D) Microscopy images of select C. difficile conditions from panel C: no antibiotic at 24 and 48 h and 1.5 μg/mL metronidazole at 48 h. Exponential phase C. difficile cells in presence of subMIC of metronidazole do not show morphological difference from exponential phase C. difficile cells grown in absence of antibiotic. (E) Line plots of C. difficile in pair with Bacteroides thetaiotaomicron over time. (Top) C. difficile OD600 in the presence of 0 μg/mL or 1.5 μg/mL metronidazole. (Bottom) C. difficile subMIC fold change in the presence of 1.5 μg/mL metronidazole. Gray horizontal line at y = 1 indicates no change in growth compared to no antibiotic condition. SubMIC fold change calculated as in panels AB. Points represent biological replicates. Line represents average of n = 4 biological r [file pbio.3002100.s003.tif]

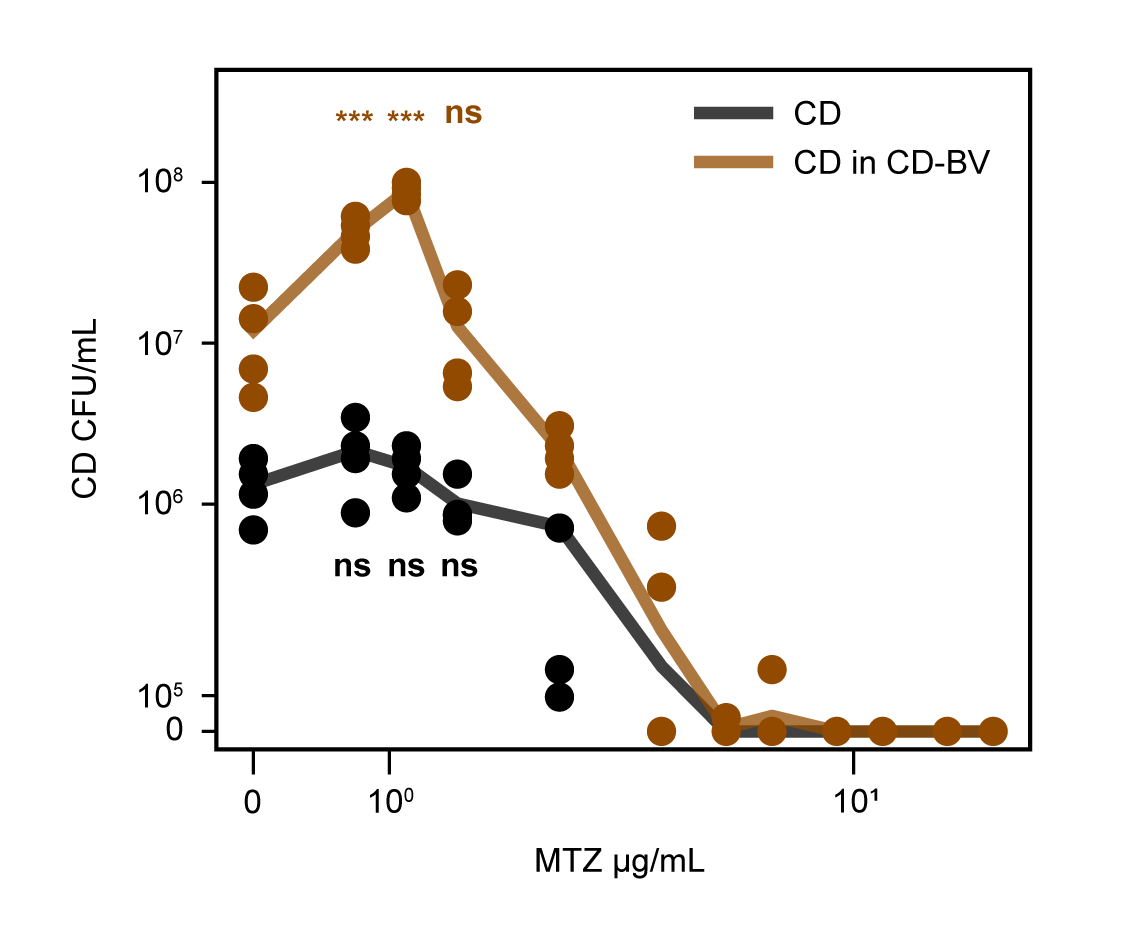

Supplement: S4 Fig — Line plot of C. difficile abundance in presence of metronidazole in (black) monoculture or in (brown) coculture with B. vulgatus. Data points indicate biological replicates. Lines indicate the average of n = 4 biological replicates. Asterisks indicate significance between C. difficile CFU/mL at given concentration and C. difficile CFU/mL in the absence of antibiotic (*P < 0.05, **P < 0.01, ***P < 0.001, ns P > 0.05). The data underlying this figure can be found in DOI: 10.5281/zenodo.7626486. (TIF) [file pbio.3002100.s004.tif]

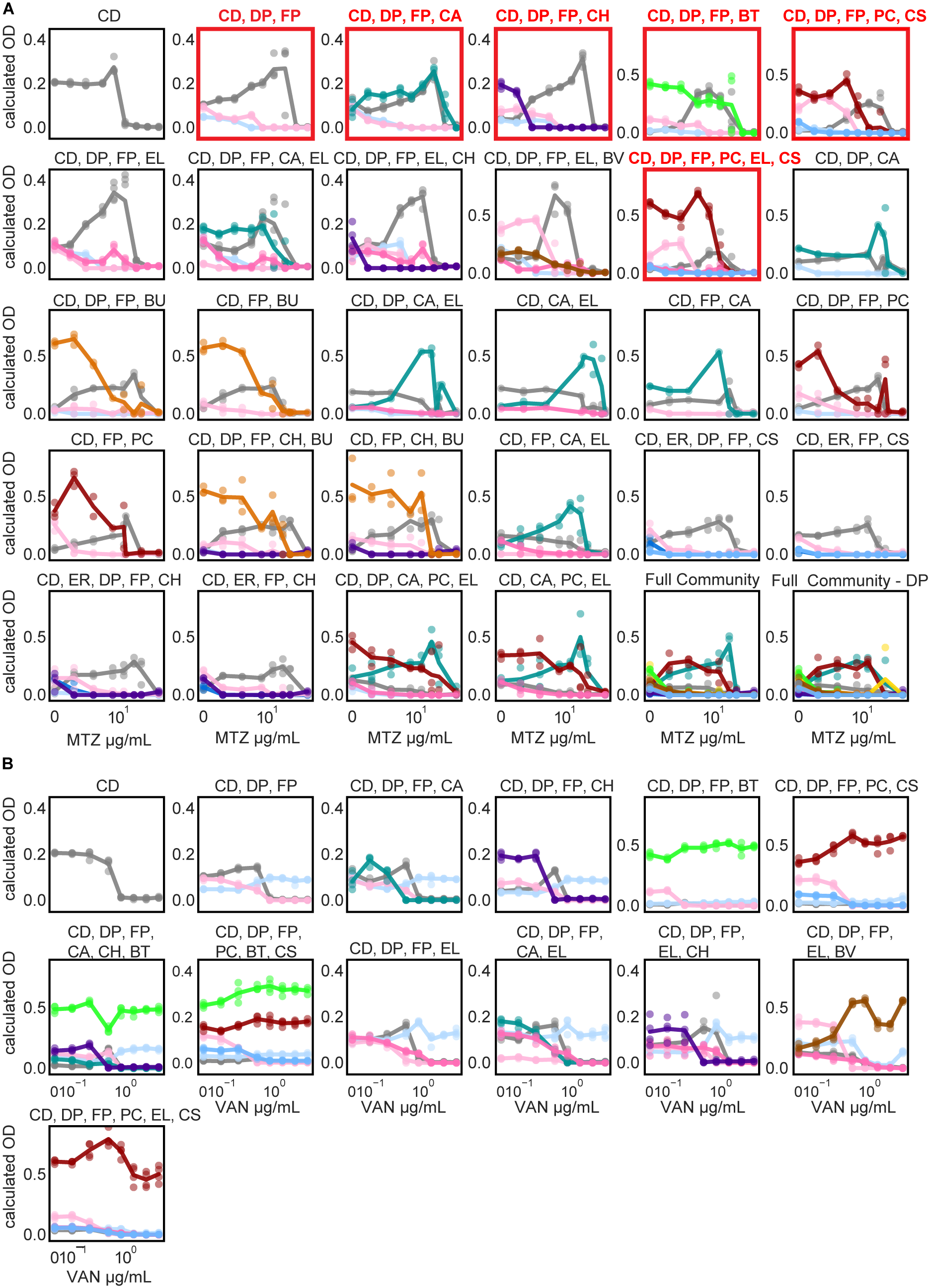

Supplement: S5 Fig — (A, B) Line plots of absolute abundance across A metronidazole (MTZ) and B vancomycin (VAN) concentrations at 48 h. Each x-axis is semi-log scale. Y-axis is calculated OD600 (OD600 multiplied by relative abundance based on 16S sequencing). Data points indicate biological replicates. Lines indicate the average of n = 1 to n = 4 biological replicates in panel A and n = 3 to n = 4 biological replicates in panel B. Color indicates species, see Fig 1C. Red borders indicate communities with ≥4-fold change in C. difficile MIC (see S1 Table). The data underlying all panels in this figure can be found in DOI: 10.5281/zenodo.7626486. (TIF) [file pbio.3002100.s005.tif]

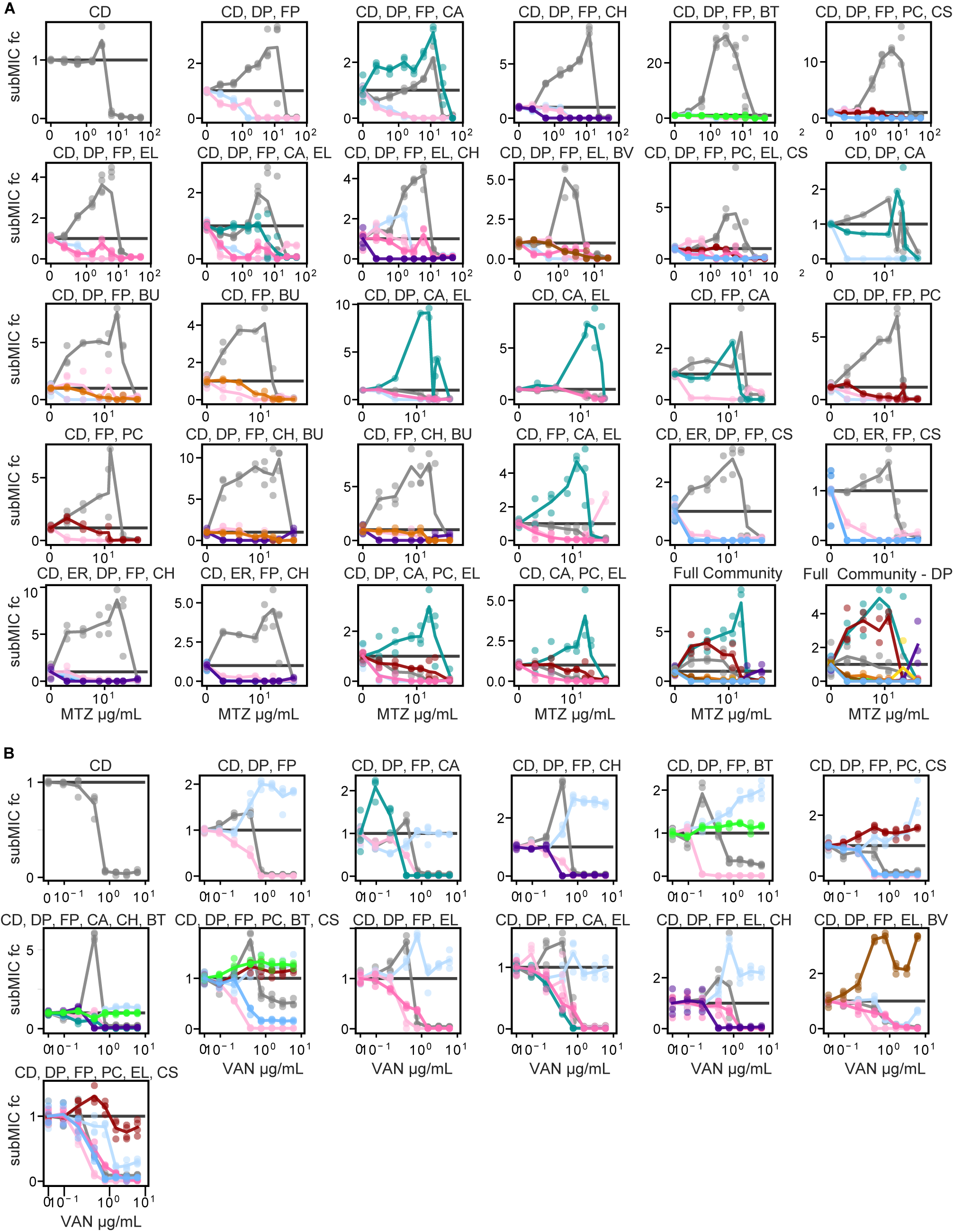

Supplement: S6 Fig — (A, B) Line plots of subMIC fold change in metronidazole (MTZ) or vancomycin (VAN) at 48 h. SubMIC fold change is calculated as the species absolute abundance in a given subMIC divided by the species absolute abundance in the absence of the antibiotic. Black horizontal line at y = 1 indicates no change in growth compared to no antibiotic condition. Each x-axis is semi-log scale. Data points indicate biological replicates. Lines indicate the average of n = 1 to n = 4 biological replicates in panel A and n = 3 to n = 4 biological replicates in panel B. Color indicates species, see Fig 1C. The data underlying all panels in this figure can be found in DOI: 10.5281/zenodo.7626486. (TIF) [file pbio.3002100.s006.tif]

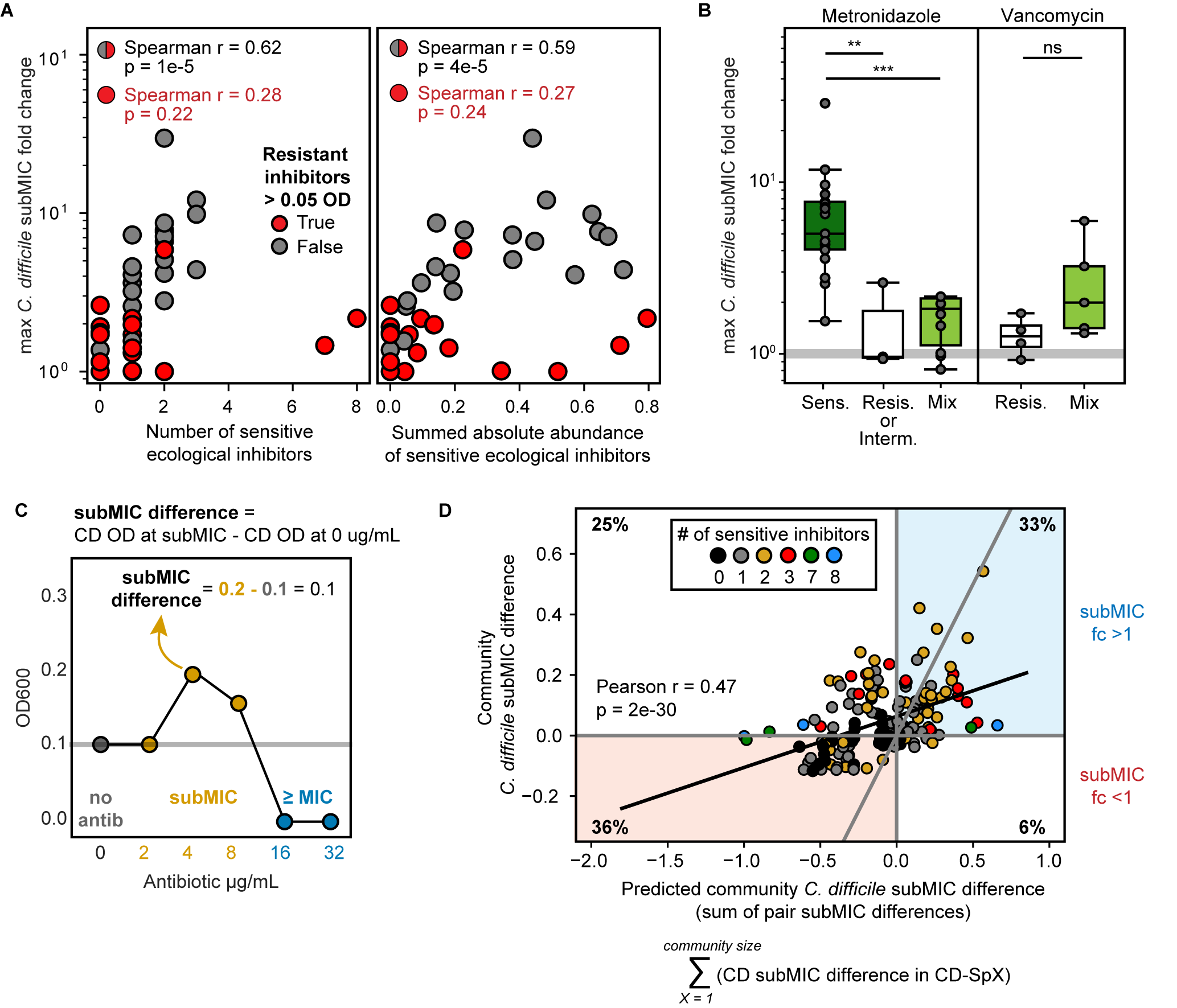

Supplement: S7 Fig — (A) Scatterplots of maximum C. difficile subMIC fold change in multispecies communities at 48 h as a function of the number (left) or abundance (right) of sensitive biotic inhibitors for both metronidazole and vancomycin. The x-axis represents the number of sensitive biotic inhibitors in a given community in the beginning of the experiment (left) or summed absolute abundance of sensitive biotic inhibitors in a given community at 48 h in the absence of the antibiotic (right). The average subMIC fold change was calculated for each subMIC concentration by computing the average C. difficile absolute abundance in a given subMIC divided by the average C. difficile absolute abundance in the absence of the antibiotic, average of n = 1 to n = 4 biological replicates. The maximum subMIC fold change (y-axis) is the maximum of the average subMIC fold change across all subMICs. The sensitive and resistant inhibitors are determined as in Fig 3F. Red points indicate communities where the sum of absolute abundance of resistant biotic inhibitors at 48 h was greater than 0.05 OD600. Spearman correlation annotated for all data points (black) and for only communities with resistant inhibitors >0.05 OD600 (red). (B) Box plot of the maximum subMIC fold change for C. difficile in multispecies communities. The maximum subMIC fold change (y-axis) is calculated as in panel A. Each data point represents 1 community. Community type (x-axis) determined as in Fig 3F. Gray horizontal line at y = 1 indicates no change in growth compared to the no antibiotic condition. Asterisks indicate a significant difference (*P < 0.05, **P < 0.01, ***P < 0.001) according to a one-sided Mann–Whitney U test. (C) Schematic of subMIC difference metric. The x-axis is semi-log scale. Gray horizontal line at y = 1 indicates no change in growth compared to the no antibiotic condition. (D) Scatterplots comparing the predicted value of the C. difficile subMIC difference in a given community (x-axis) with average experim [file pbio.3002100.s007.tif]

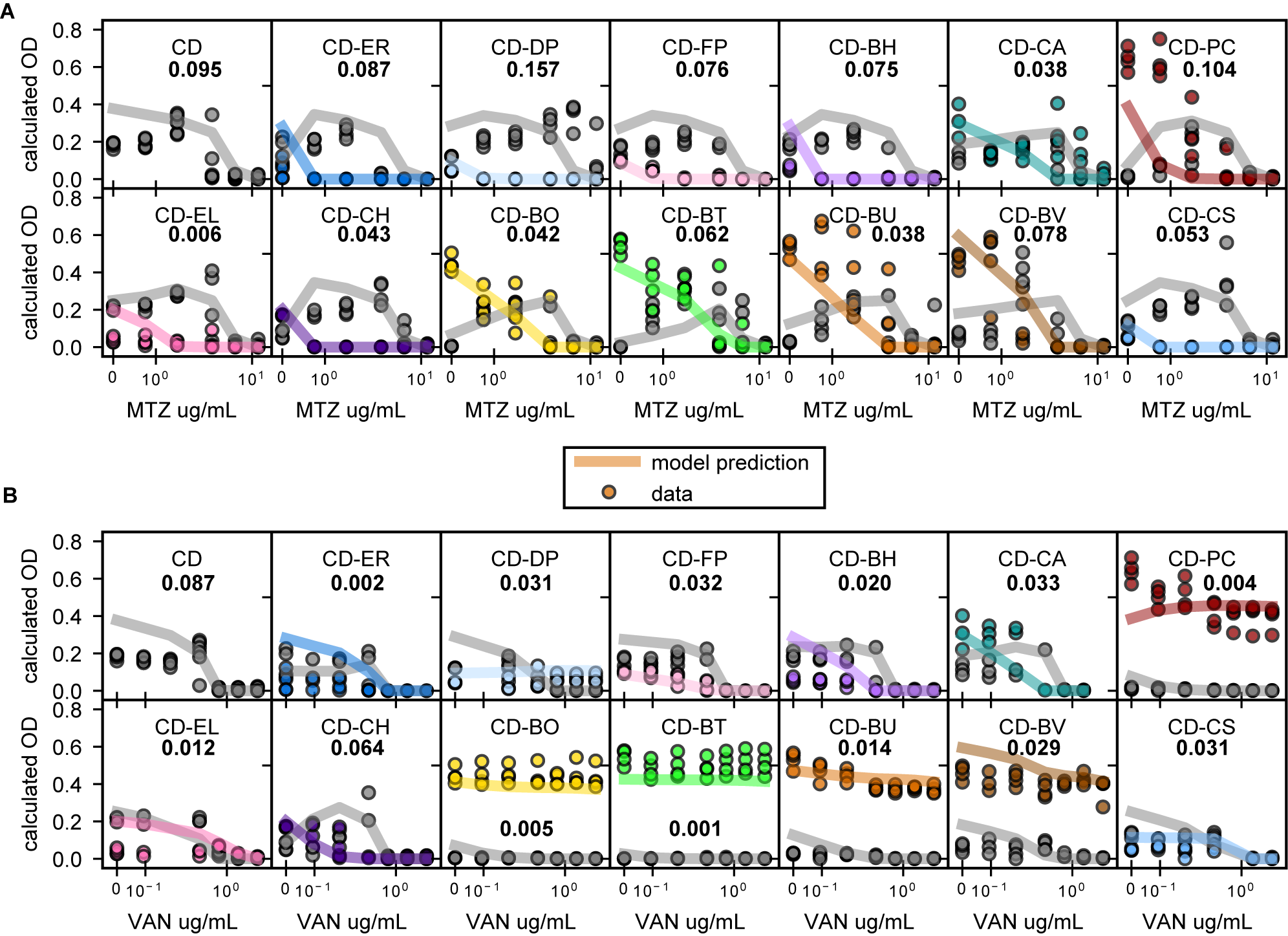

Supplement: S8 Fig — (A, B) Line plots of model prediction of absolute abundance of C. difficile and gut microbe pairwise communities at 48 h in presence of A metronidazole (MTZ) or B vancomycin (VAN). Each x-axis is semi-log scale. Y-axis is calculated OD600 (OD600 multiplied by relative abundance from 16S sequencing). Points indicate experimental data. Lines indicate model simulations. Color indicates species, see Fig 1C. Bold number is the sum of squared errors for C. difficile (square of the difference between model OD600 and the average experimental OD600, summed across all concentrations). The data underlying all panels in this figure can be found in DOI: 10.5281/zenodo.7626486. (TIF) [file pbio.3002100.s008.tif]

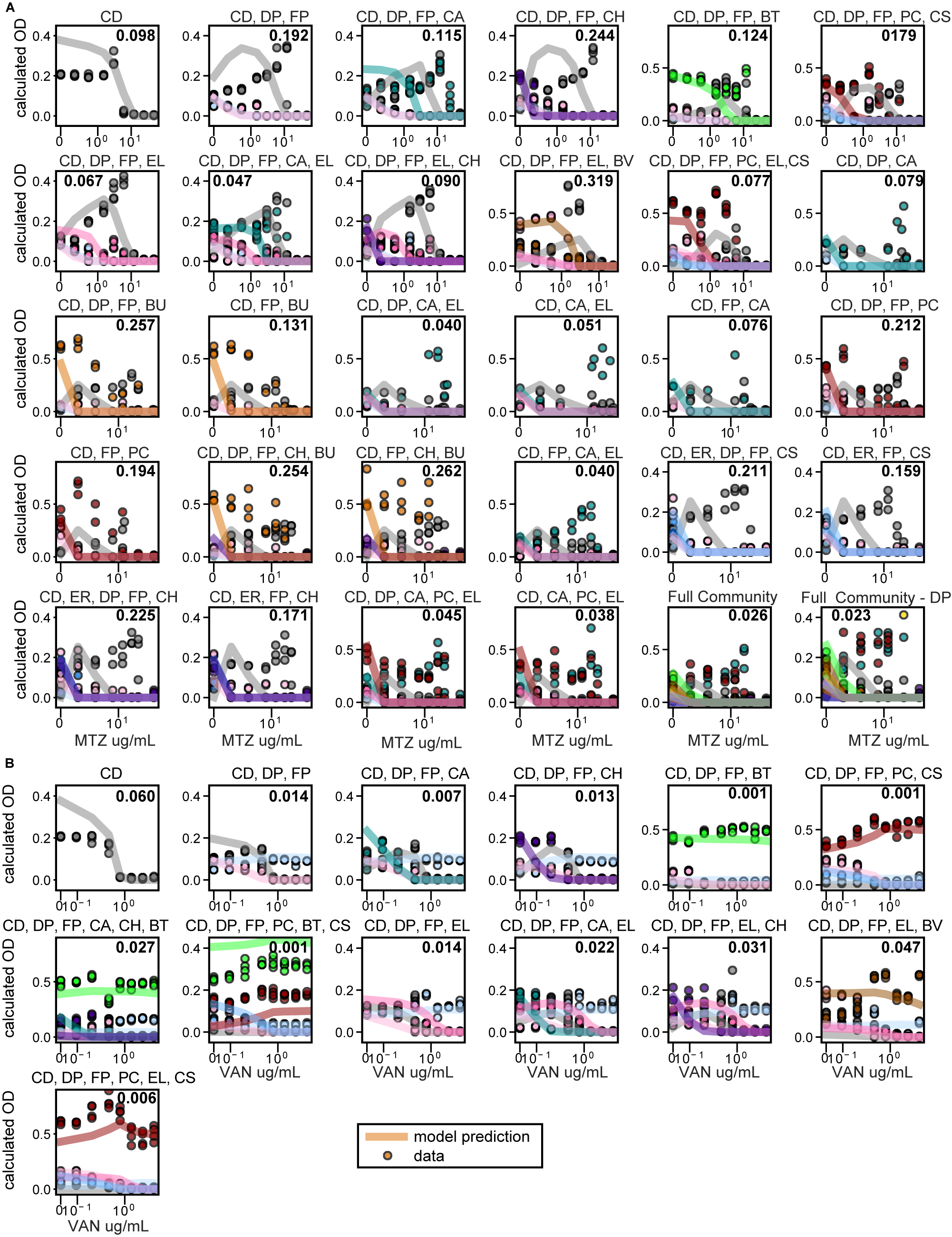

Supplement: S9 Fig — (A, B) Line plots of model prediction of absolute abundance of species in multispecies communities at 48 h in the presence of A metronidazole (MTZ) or B vancomycin (VAN). Each x-axis is semi-log scale. The y-axis denotes calculated OD600 (OD600 multiplied by relative abundance from 16S sequencing). Data points indicate experimental data. Lines indicate model simulations. Color indicates species, see Fig 1C. Bold number is sum of squared errors for C. difficile (square of difference between model OD600 and average experimental OD600, summed across all concentrations). The data underlying all panels in this figure can be found in DOI: 10.5281/zenodo.7626486. (TIF) [file pbio.3002100.s009.tif]

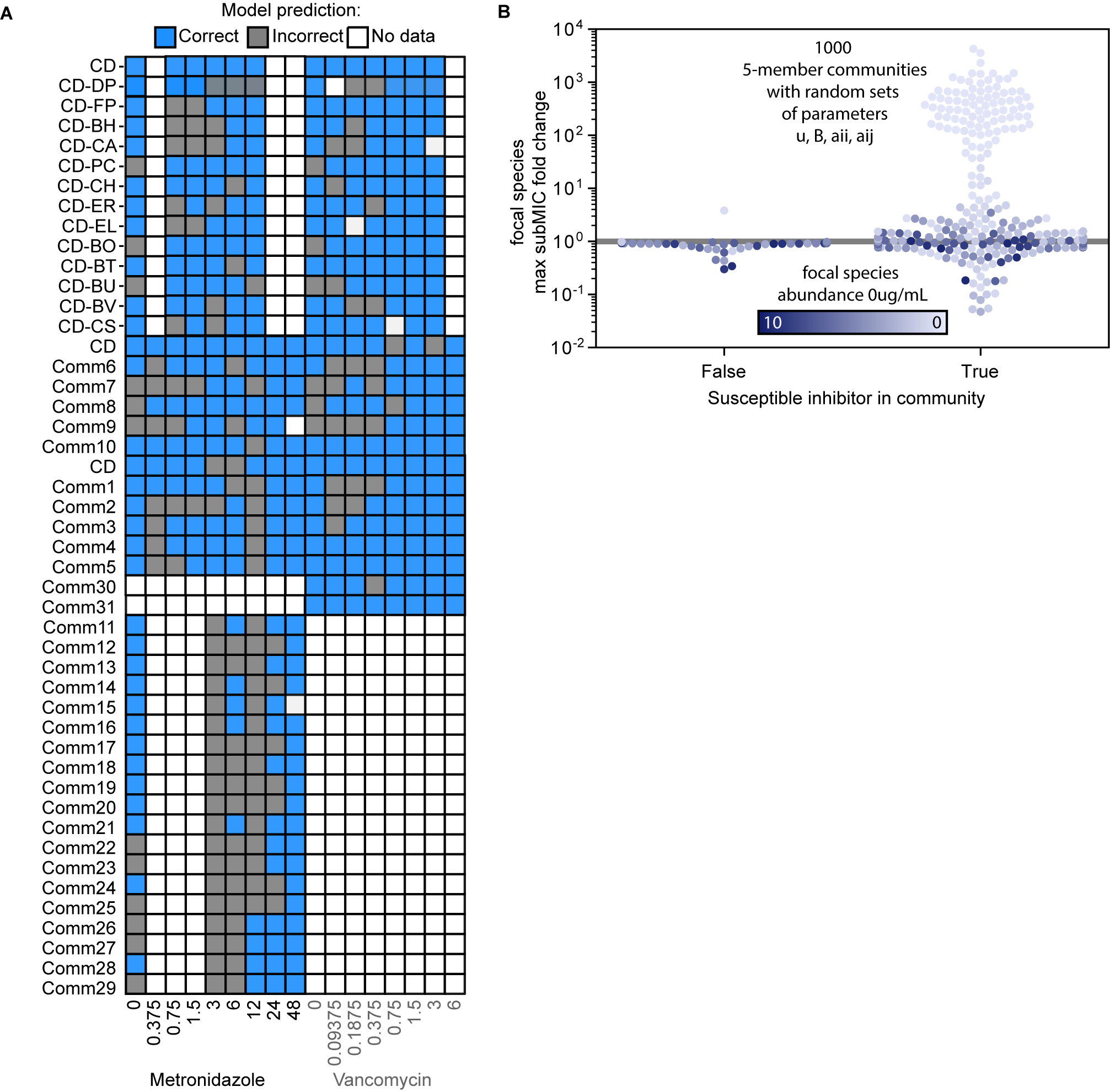

Supplement: S10 Fig — (A) Antibiotic gLV model prediction of pairwise and multispecies community data in the presence of each antibiotic concentration. These predictions are summarized in the blue “gLV + monospecies antib susceptibility” bar in Fig 4B. (B) Simulated maximum subMIC fold change for a focal species cocultured with a 4 other species for 1,000 randomly generated parameter sets. All parameters were randomized between a set of bounds. Bounds: aji (−1.25, 1.25), growth rates (0, 1), intraspecies interactions (−1.25, 0), and antibiotic susceptibility (−6, 0). Color of the data point indicates the abundance of focal species in the community in the absence of antibiotics (where light colored data points indicate inhibition of a focal species). A community is classified as containing a susceptible inhibitor if for any non-focal species j, Bj < −0.1 and afocal,j < −0.1. Gray horizontal line at y = 1 indicates no change in growth compared to the no antibiotic condition. The data and modeling scripts underlying all panels in this figure can be found in DOI: 10.5281/zenodo.7726490. (TIF) [file pbio.3002100.s010.tif]

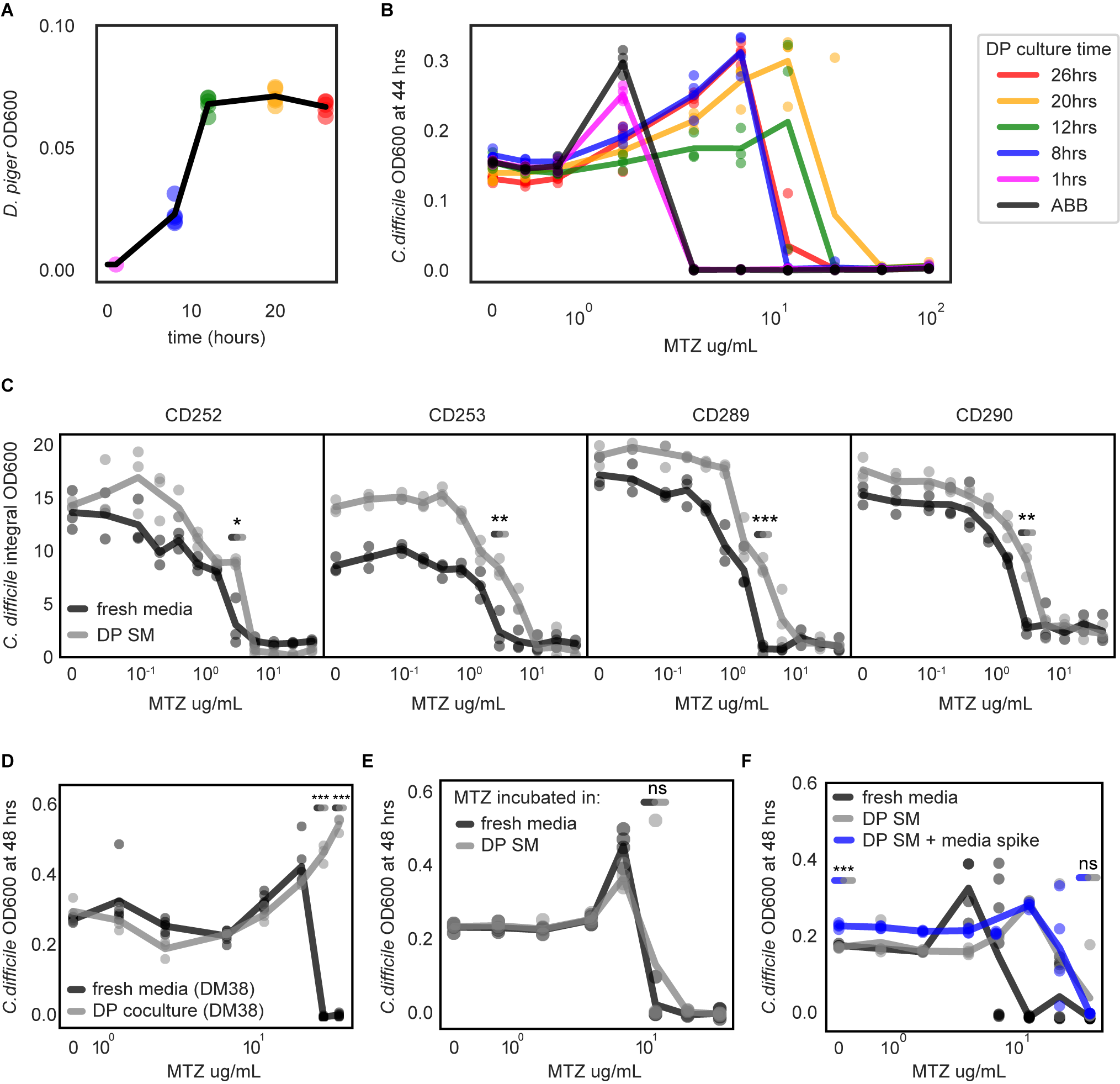

Supplement: S11 Fig — (A) Line plot of the growth curve of D. piger. Colored data points indicate the time at which 5 separate D. piger spent media samples were harvested. Data points represent biological replicates. The line represents the mean of n = 5 biological replicates. (B) Line plot of C. difficile abundance at 41 h in 5 different D. piger spent medias in the presence of metronidazole (MTZ). The x-axis is semi-log scale. Data points indicate biological replicates. Line represents the mean of n = 4 biological replicates. (C) Line plot of the abundance of 4 clinical C. difficile isolates in the presence of metronidazole. Each x-axis is semi-log scale. The y-axis denotes the integral of C. difficile OD600 from 0 to 48 h. Data points indicate biological replicates. The line represents the mean of n = 3 biological replicates. Asterisks indicate a significant difference (*P < 0.05, **P < 0.01, ***P < 0.001) according to an unpaired t test. (D) Line plot of C. difficile abundance at 48 h in a rich chemically defined media (“DM38”) in the presence of metronidazole. The x-axis is semi-log scale. For the D. piger coculture condition, OD600 denotes calculated OD600 (OD600 multiplied by relative abundance from 16S rRNA gene sequencing). Data points indicate biological replicates. The line represents the mean of n = 6 (fresh media) or n = 3 (DP SM) biological replicates. Asterisks indicate a significant difference (*P < 0.05, **P < 0.01, ***P < 0.001) according to an unpaired t test. (E) Line plot of C. difficile abundance at 48 h in fresh media in the presence of metronidazole that was incubated in fresh media or D. piger spent media. The x-axis is semi-log scale. Data points indicate biological replicates. Line represents the mean of n = 4 biological replicates. No significant difference (“ns”, p > 0.05) according to an unpaired t test. (F) Line plot of C. difficile abundance at 48 h in fresh media, D. piger spent media, or D. piger spent media with a fresh media spike. The x-axis is semi-l [file pbio.3002100.s011.tif]

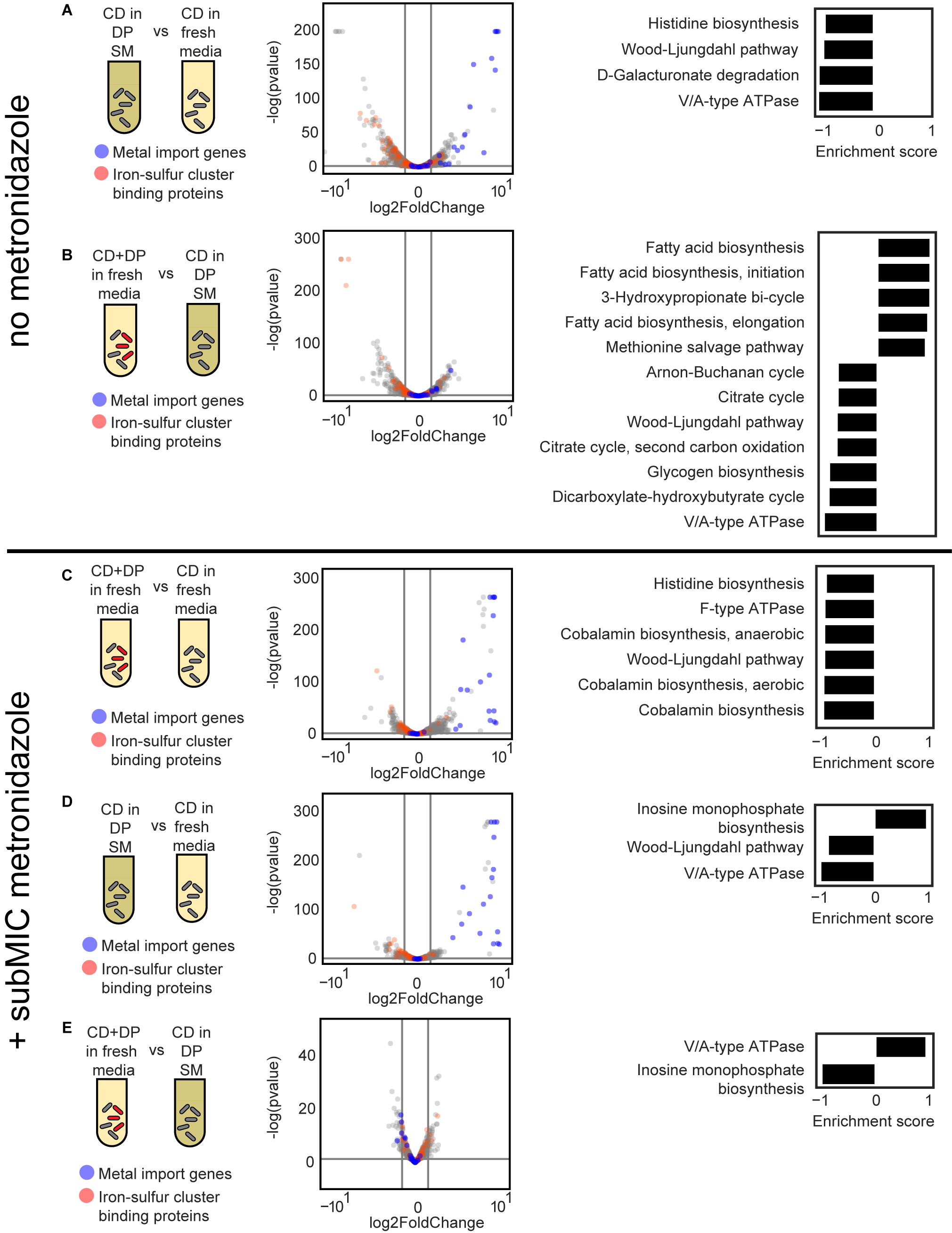

Supplement: S12 Fig — (A–E) Left: Schematic of 2 conditions being compared. Middle: Volcano plot of the fold change in C. difficile transcript abundance. Gray vertical lines indicate 2-fold change (1 in log2) and gray horizontal line indicates statistical significance (p = 0.05). Blue indicates genes annotated to be involved in metal import. Red indicates genes predicted to contain iron-sulfur clusters by MetalPredator. Right: Enriched KEGG modules in C. difficile. All KEGG pathways with significant enrichment scores from Gene Set Enrichment Analysis (GSEA) are shown. The data underlying all panels in this figure can be found in S3 and S4 Tables. (TIF) [file pbio.3002100.s012.tif]

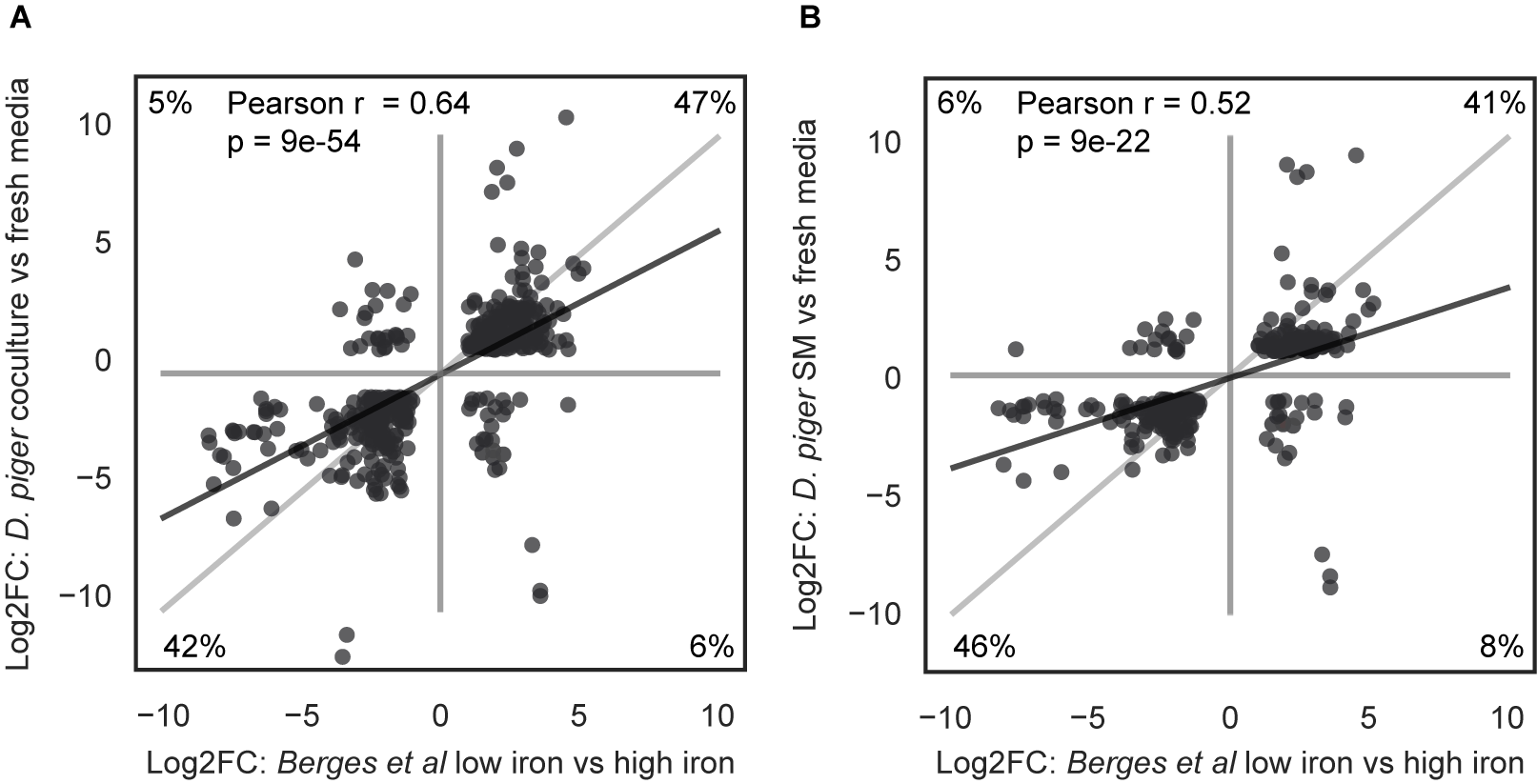

Supplement: S13 Fig — (A, B) Scatterplots to evaluate the patterns in gene expression for gene orthologs between CDR20291 (this study) and CD630Δerm (Berges and colleagues [35]). Genes only shown if differentially expressed in both studies. X-axis: log2 fold change between low iron (0.2 μM) and high iron (15 μM) media in Berges and colleagues [35]. Y-axis: log2 fold change between C. difficile in coculture with D. piger (panel A) or C. difficile in D. piger spent media (SM) (panel B) and C. difficile in fresh media in this study. Each data point indicates a gene. Percentages indicate percentage of genes in each quadrant. Black line indicates best fit linear regression for all data points. Gray line is y = x. The data underlying all panels in this figure can be found in S3 Table. (TIF) [file pbio.3002100.s013.tif]

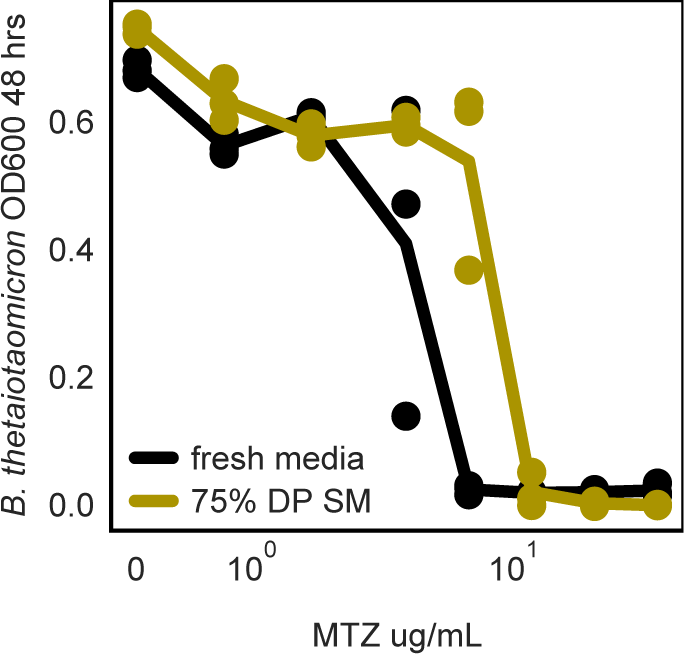

Supplement: S14 Fig — Line plot of B. thetaiotaomicron abundance at 48 h in fresh media or in 75% D. piger spent media, 25% fresh media. The x-axis is semi-log scale. Data points represent biological replicates. Lines indicate the average of n = 3 biological replicates. The data underlying this figure can be found in DOI: 10.5281/zenodo.7626486. (TIF) [file pbio.3002100.s014.tif]

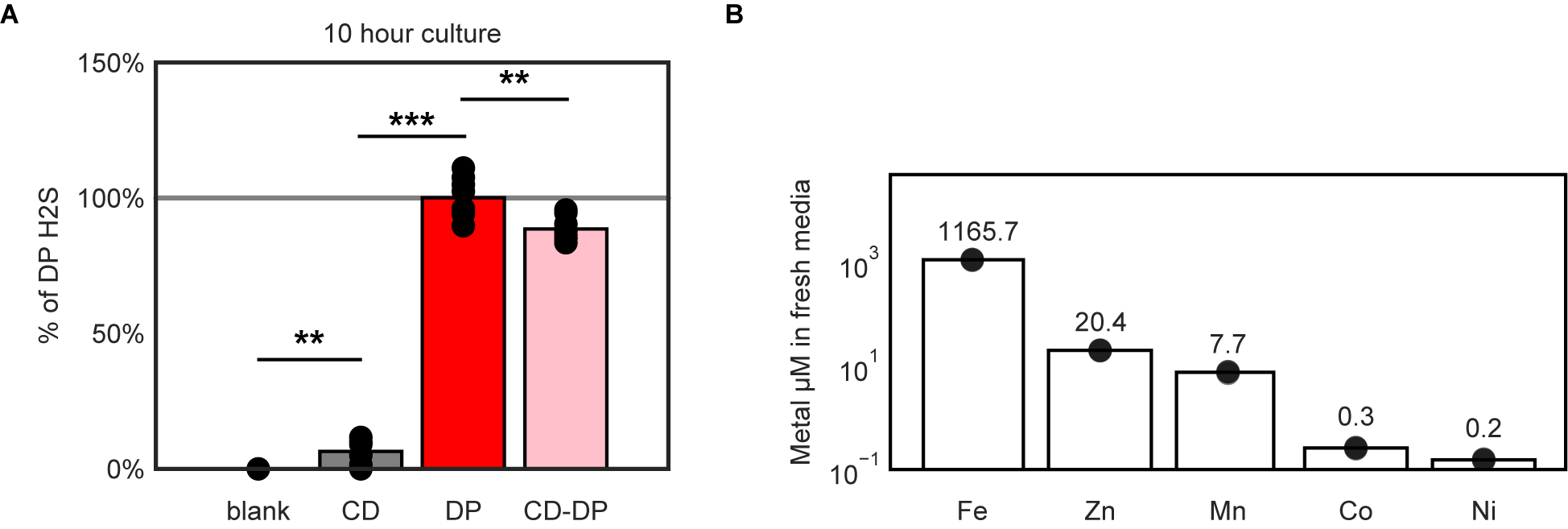

Supplement: S15 Fig — (A) Bar plot of the amount of hydrogen sulfide in multiple conditions after 10 h of incubation. “Blank” represents a fresh media control. The y-axis denotes the percentage of the amount of hydrogen sulfide in the D. piger monoculture. Data points represent the mean of n = 2 technical replicates. Bar represents the mean of n = 8 biological replicates. Asterisks indicate significant difference (*P < 0.05, **P < 0.01, ***P < 0.001, “ns” P > 0.05) according to an unpaired t test. (B) Bar plot of the concentration of divalent transition metals in fresh media. Points represent the mean of n = 2 technical replicates. Bar represents the mean of n = 3 biological replicates. The data underlying all panels in this figure can be found in DOI: 10.5281/zenodo.7626486. (TIF) [file pbio.3002100.s015.tif]

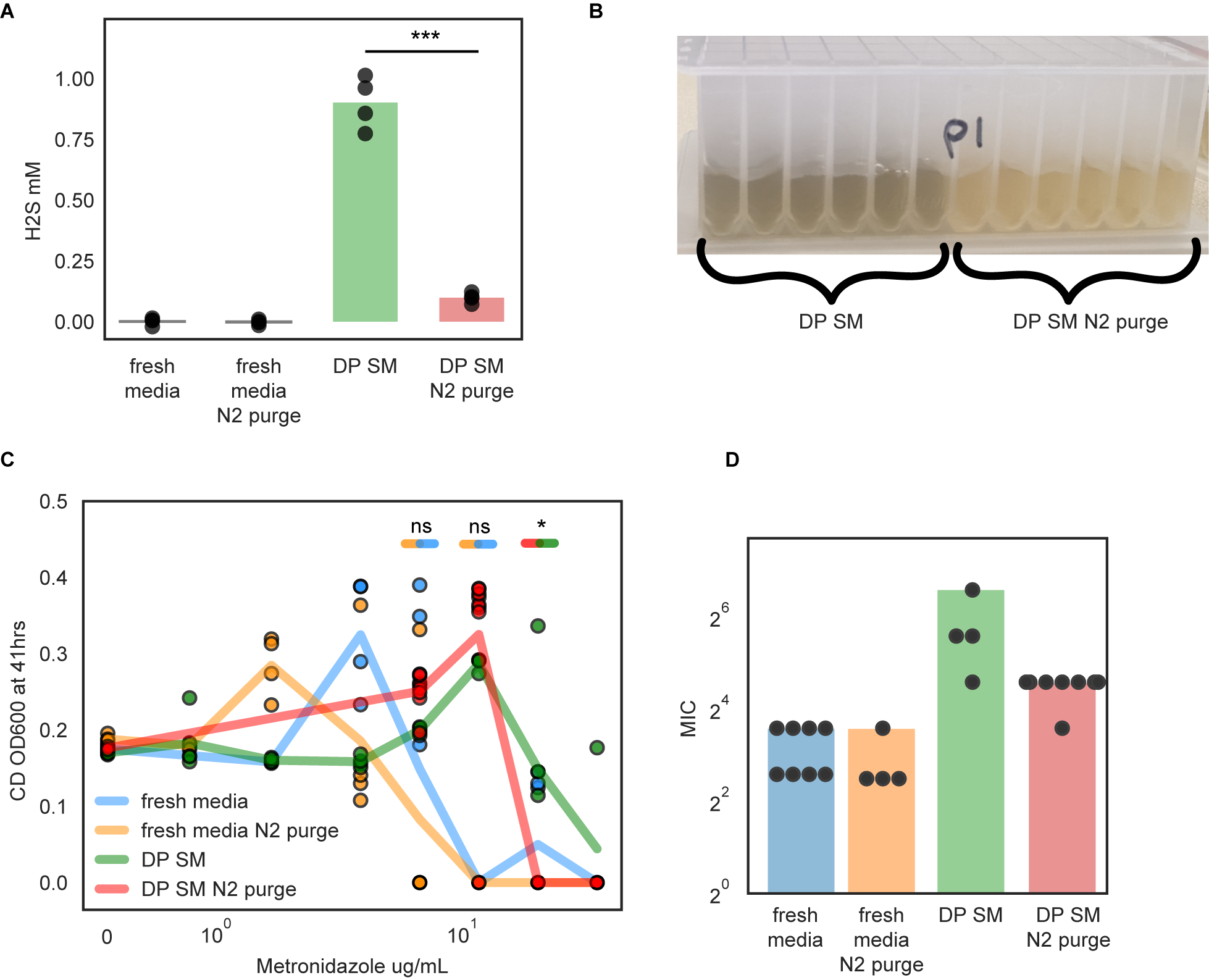

Supplement: S16 Fig — (A) Bar plot of hydrogen sulfide concentration in different medias. Data points represent n = 4 biological replicates. Bar represents the average of biological replicates. (B) Image of D. piger spent media 2 h after nitrogen purge (right) or no treatment (left). Precipitation of a black ferrous sulfide turns the media a darker color. (C) Line plot of C. difficile OD600 at 41 h in different medias in the presence of metronidazole. The x-axis is semi-log scale. Data points represent biological replicates. Lines indicate the average of n = 4 to n = 8 biological replicates. (D) Bar plot displaying MIC of data shown in C. Data points represent the MIC of n = 4 to n = 8 biological replicates. Bar represents the MIC determined based on an average OD600 of n = 4 to n = 8 biological replicates. The data underlying panels ACD in this figure can be found in DOI: 10.5281/zenodo.7626486. (TIF) [file pbio.3002100.s016.tif]

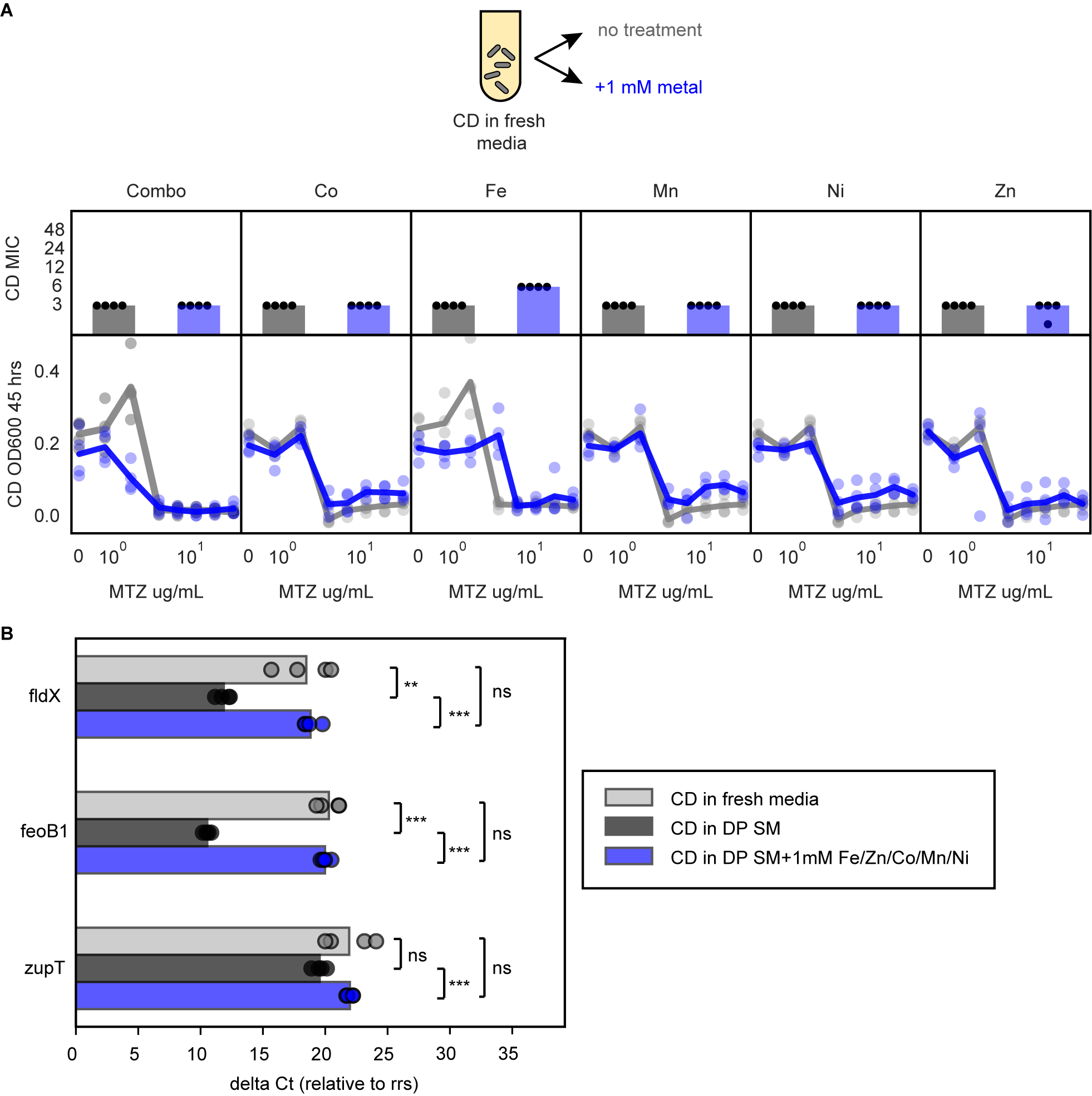

Supplement: S17 Fig — (A) Line plots and bar plots of C. difficile metronidazole susceptibility in fresh media with and without metal supplementation. (Top) Bar plots of C. difficile metronidazole MIC in untreated fresh media (gray) and media with 1 mM metal supplementation (blue). In the metal combination condition (“Combo”), all 5 metals were supplemented at 1 mM. Data points represent the MIC of n = 4 biological replicates. Bar represents the MIC determined based on an average OD600 of n = 4 biological replicates. (Bottom) Line plots of C. difficile OD600 at 45 h in untreated fresh media (gray) or media supplemented with 1 mM metal (blue) in the presence of metronidazole (MTZ). Each x-axis is semi-log scale. Data points represent biological replicates. Lines indicate the average of n = 4 biological replicates. (B) Bar plot of delta Ct (ΔCt) values of 3 genes in C. difficile grown in fresh media, D. piger spent media (DP SM), or D. piger spent media with metal supplementation based on qRT-PCR. Data points represent biological replicates, with each point calculated as the average of 3 technical replicates. Bar indicates the average of n = 4 biological replicates. Asterisks indicate significant difference (*P < 0.05, **P < 0.01, ***P < 0.001, “ns” P > 0.05) according to an unpaired t test. The data underlying all panels in this figure can be found in DOI: 10.5281/zenodo.7626486. (TIF) [file pbio.3002100.s017.tif]

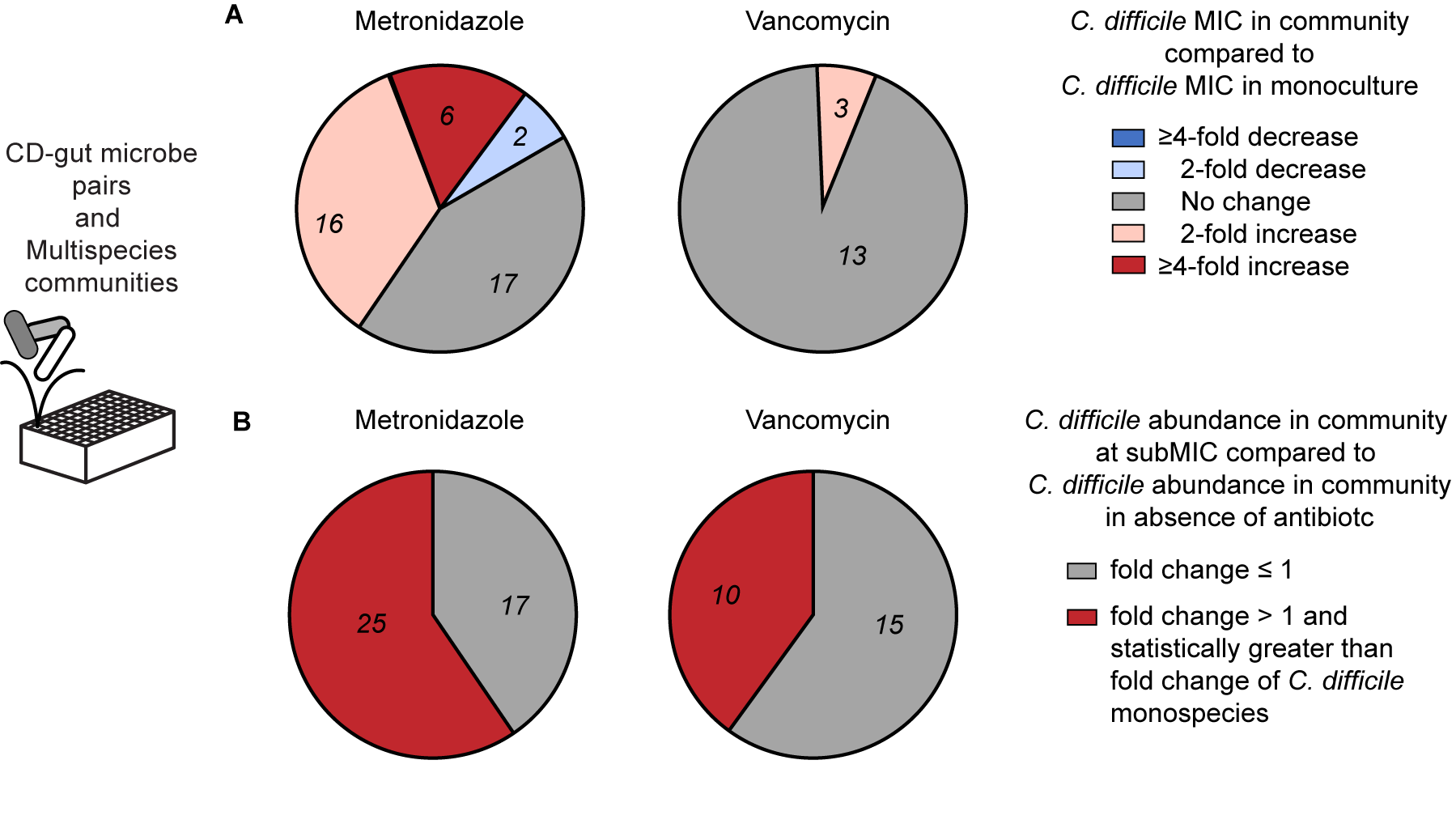

Supplement: S18 Fig — (A) Pie charts of the change in C. difficile MIC in pairwise and multispecies communities compared to monoculture. (B) Pie charts of the growth enhancement of C. difficile in pairwise and multispecies communities. Growth enhancement is defined as a maximum subMIC fold change that is greater than 1 and is greater than the maximum subMIC fold change in C. difficile monoculture for that antibiotic (i.e., greater than 1.61 for metronidazole). The data underlying all panels in this figure can be found in DOI: 10.5281/zenodo.7626486. (TIF) [file pbio.3002100.s018.tif]
